# Noncoding RNA-regulated gain-of-function of *STOX2* in Finnish pre-eclamptic families

**DOI:** 10.1038/srep32129

**Published:** 2016-08-24

**Authors:** Cees BM Oudejans, Ankie Poutsma, Omar J. Michel, Hari K. Thulluru, Joyce Mulders, Henri J. van de Vrugt, Erik A. Sistermans, Marie van Dijk

**Affiliations:** 1 Department of Clinical Chemistry, VU University Medical Center, Amsterdam, the Netherlands; 2 Department of Clinical Genetics, VU University Medical Center, Amsterdam, The Netherlands

## Abstract

The familial forms of early onset pre-eclampsia and related syndromes (HELLP) present with hypertension and proteinuria in the mother and growth restriction of the fetus. Genetically, these clinically similar entities are caused by different founder-dependent, placentally-expressed paralogous genes. All susceptibility genes (*STOX1*, *lincHELLP*, *INO80B*) identified so far are master control genes that regulate an essential trophoblast differentiation pathway, but act at different entry points. Many genes remain to be identified. Here we demonstrate that a long non-coding RNA (lncRNA) within intron 3 of the *STOX2* gene on 4q35.1 acts as a permissive cis-acting regulator of alternative splicing of *STOX2*. When this lncRNA is mutated or absent, an alternative exon (3B) of *STOX2* is included. This introduces a stop codon resulting in the deletion of a highly conserved domain of 64 amino acids in the C-terminal of the STOX2 protein. A mutation present within a regulatory region within intron 1 of *STOX2* has the same effect after blocking with CRISPR technology: transcripts with exon 3B are upregulated. This proces appears related to transcriptional control by a chromatin-splicing adaptor complex as described for *FGFR2*. For *STOX2*, *CHD5*, coding for a chromodomain helicase DNA binding protein, qualifies as the chromatin modifier in this process.

For pre-eclampsia (new onset hypertension with proteinuria during pregnancy), the existence of multiple susceptibility loci (4q34, 2p13, 9p13, 2p25, 10q22, 12q23) in different founder populations (Australia, Iceland, Norway, Finland, Netherlands) is known for decades, but with limited success in the identification of the genes involved^1^,^2^,^3^,^4^,^5^,^6^.

We postulated that the synteny of the chromosomal regions on 10q22, 2p13 and 4q24-35 with genome wide linkage to pre-eclampsia indicates an evolutionary fundamental mechanism in mammals subject to conserved genetic pressure that evolved as a consequence of hemochorial placentation^4^,^5^,^6^. Genetically, this is reflected by different founder effects (Iceland, Netherlands, Australia, Finland) in syntenic chromosomal regions on different chromosomes (2p13, 10q22, 4q24-35) containing paralogous genes (*STOX1, INO80B*) causing the same disease (pre-eclampsia)^1^,^2^,^3^,^4^,^5^,^6^. Functionally, these regions harbor fetally inherited, placentally expressed susceptibility genes for pre-eclampsia that -when defective- cause trophoblast dysfunction in early pregnancy by disrupting a common pathway essential for placentation^1^,^5^. This pathway involves the transition from proliferative, non-invasive extravillous trophoblast cells to differentiated invasive cells and is central in the establishment of a connection between the maternal and fetal circulations during the first trimester^6^. When defective, a cascade of events follows that ultimately -months after the initial event- leads to maternal symptoms (Supplementary File 1).

Evidence for this model of founder-dependent segregation of paralogous genes for the familial, genetic forms of the same disease (early onset pre-eclampsia) is accumulating. The *EGR4-TET3-INO80B-DQX1-HK2-TACR1* region on 2p13 linked with pre-eclampsia in Iceland is paralogous to the *EGR2-TET1-STOX1-DDX50-HK1-TACR2* region on 10q22 linked with pre-eclampsia in Dutch families^4^. The *STOX1* gene located between *TET1-HK1* in the 10q22 region codes for a winged helix transcription factor and causes early-onset pre-eclampsia with intra-uterine growth restriction in Dutch families^1^. This has been confirmed experimentally; placental overexpression of *Stox1* in transgenic mice caused pre-eclampsia with all features of the human disease^7^. The *STOX2* paralog, *INO80B*, located between *TET3-HK2* in the 2p13 region contains a novel winged helix domain and has been identified as the pre-eclampsia susceptibility gene in Icelandic families^6^.

This founder-dependent segregation of paralogous pre-eclampsia genes located in syntenic loci likely involves the Finnish and Australian loci on chromosome 4 as well. The order and identity of the paralogous genes on 4q containing putative pre-eclampsia genes correspond with the syntenic loci on 10q22 and 2p13 containing confirmed pre-eclampsia genes (Fig. 1). In Finnish pre-eclampsia families, non-parametric linkage (NPL) analysis of 174 individuals from 15 families with 72 affected mothers showed genome wide linkage with chromosome 4 in 8 (53%) of these families. Two loci were identified on 4q, depending on the type of analysis: fetal or maternal^8^,^9^.

In the fetal (placental) model, the fetal genotype determines the maternal phenotype. A fetal gene aberrantly expressed in the placenta leads to extravillous trophoblast dysfunction in early pregnancy with inappropriate establishment of the fetal-maternal hemochorial connection (Supplementary File 1). In the maternal model, the maternal genotype parallels the maternal phenotype and follows conventional genetic rules. In the Finnish study, using maternal phenotypes (assuming a maternal effect gene operating in the mother), the highest peak was seen at 158.20 cM (4q32) near marker D4S413 (NPL = 3.13, p = 0.0009, LOD = 2.09). Using fetal phenotypes (assuming a fetal effect gene operating in the placenta), the highest peak was seen at 109.60 cM (4q24) near marker D4S1572 (NPL = 2.50, p = 0.006, LOD = 0.78)^8^,^9^. Region 1 (4q24) contains *TACR3* and *TET2*; region 2 (4q32) flanks the *DDX60*-*STOX2* region (Fig. 1). The latter region is near the 2.8 cM locus (D4S450-D4S610) linked (LOD 2.91) (4q34) with pre-eclampsia in Australian families^10^.

The syntenic locus model assumes that a recombination event of chromosome 4 caused separation of the chromosome 2 and chromosome 10 regions into two separate regions that underlie the two linkage peaks on 4q in Finnish families (Fig. 1). Guided by this syntenic model, we explored both 4q regions for the presence of a pre-eclampsia susceptibility gene in Finnish families.

## Results

### A fetal effect gene for pre-eclampsia is present on 4q near markers D4S1597-D4S415

We started by reanalyzing the identity-by-descent allele-sharing pattern in the pedigrees of two informative families (# 7 and 13) (Supplementary File 2). These families were selected for their confirmed linkage to 4q, the availability of DNA from multiple generations (n = 4) and the presence of affected sibpairs in both families. The original microsatellite marker data (n = 19) in and near the two regions with the highest lod scores (4q24, 4q32)^8^,^9^ were used. Both the fetal and maternal models were tested.

The pattern observed in the affected sibpairs indicated autosomal recessive inheritance of a fetal effect gene on 4q32-q34 (Supplementary File 3). The minimal critical region (MCR) as defined by the region with maximal identity-by-descent (highest number of shared markers) in the affected sibpairs of both families was located between or near markers D4S1597-D4S415. In other words, the region with the highest lod score previously identified by considering a *matern*al effect gene using *all* families fully qualified for a *fetal* effect gene in families with *linkage to 4q.*

### High-resolution marker analysis using SNPs from candidate genes narrows the minimal critical region to the *STOX2*-*DDX60* locus on 4q35.1

Given the syntenic model for pre-eclampsia along with the limited resolution of the markers used in the original linkage analysis, we argued that the second region (4q32-q34) could extend to 4q35 with inclusion of the *STOX2-DDX60* region, but required IBD analysis with markers with higher resolution. For this, an extended set of markers consisting of 73 SNPs and IN/DEL markers was obtained. These markers were identified by sequence analysis of the coding regions of six candidate genes. The genes selected (region 1: *CENPE, TACR3, CXXC4*, *TET2;* region 2*: CDKN2AIP, STOX2*) had been prioritized by the selection method of pathway-guided genome-wide meta-analysis^6^. This candidate gene approach is highly efficient as demonstrated previously^6^. It is based on the premise that the (dys)functional network in the extravillous trophoblast involves additional susceptibility genes for pre-eclampsia in different founder populations if these genes are: *i*. located in loci with confirmed genome-wide linkage (e.g. 4q24, 4q32), *ii*. transcribed in the cell central in the etiology (i.e. extravillous trophoblast) and *iii*. involved in the same pathway (i.e. trophoblast invasion linked with cell cycle exit)^6^. This pathway-guided approach allows cost-effective targeting of candidate genes for pre-eclampsia^6^. The segregation pattern seen with the additional markers identified two minimal critical regions: MCR1 in the *CENPE* gene of the *TET2-TACR3* region and MCR2 in the *STOX2* gene of the *STOX2-DDX60* region. Because the presence and sharing of the minor alleles was limited or completely absent in MCR1, while, in contrast, increased homozygosity for the minor allleles was seen in 3 out of 4 affected sibs in MCR2, we considered MCR2 the prime candidate (Supplementary File 4).

### The risk haplotype found in all affected mother-child combinations segregates with intron 3 of *STOX2* and contains transcript AK098131

**Figure 1:**
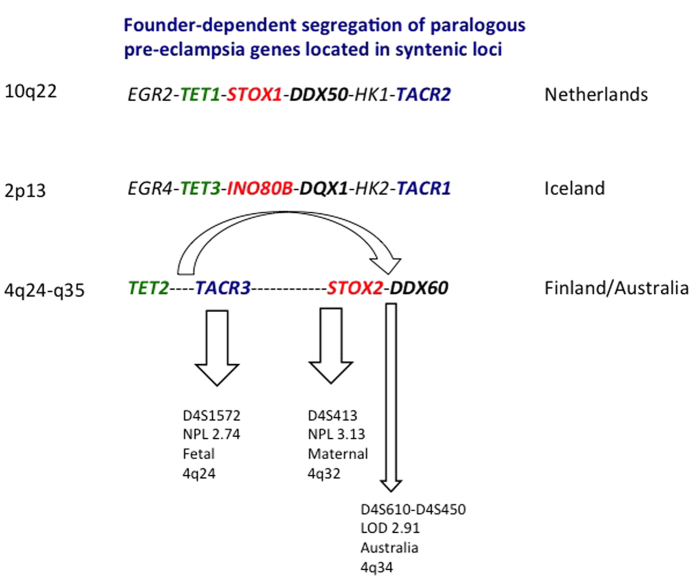
Founder-dependent segregation of paralogous pre-eclampsia genes located in syntenic loci. The order and identity of the paralogous genes in the syntenic loci on 10q22 and 2p13 causing pre-eclampsia in the Netherlands and Iceland indicate the presence of a paralog on 4q24-34 causing pre-eclampsia in Finland and Australia. For clarity, the centromere to telomere orientation of the 4q region has been reversed. The oblique arrow indicates the recombination event that took place in the 4q region. The syntenic locus model for the genes associated with pre-eclampsia in different populations (Iceland, Finland, Australia, Netherlands) assumes a two hit model during mammalian evolution in relation to placentation. The first event (duplication) increased the number of chromosomes with physical co-localization of paralogous genes (syntenic loci) from one to three chromosomes (10q22, 2p13 and q24-35). The second event (recombination) involved chromosome 4 only and led to a reversed telomeric-centromeric orientation of the 4q24-q35 region with reshuffling of the genes within this region. For clarity, not all genes in the loci presented are shown. Only the 6 informative sets of physically co-localized paralogous genes present on all three chromosomes and located within the regions linked with familial pre-eclampsia are shown.

To confirm this, we took advantage of the segregation pattern intrinsic to and unique for the placental model. Irrespective of the mode of inheritance (recessive, dominant, parent-of-origin effect), there is an absolute requirement that multiple risk alleles and their associated haplotypes need to be shared in all mother-child combinations when the mother had pre-eclampsia. For this, we performed a second round of sequencing of MCR2 to analyze the maternal segregation of the risk haplotype in all informative mother-child combinations (n = 16) in four generations (Supplementary File 2). MCR2 contains *STOX2*, the gene paralogous to *STOX1* (Fig. 1). The latter causes early-onset pre-eclampsia with intra-uterine growth restriction in Dutch families as confirmed in transgenic mice^1^,^7^. This analysis confirmed a hotspot with increased homozygosity for the minor alleles in the *STOX2* gene (Supplementary File 5). Importantly, in the majority (10/16) (62.5%), this included sharing of the minor alleles inherited from the unrelated fathers. The latter effectively rules out that the pattern observed was due to false-positivity caused by the family relatedness of the individuals analyzed. This hotspot centered at intron 3 of *STOX2*, confirmed the linkage with MCR2 in the previous analysis (Supplementary File 4), is defined by the minor alleles of eight SNPs in both families and contains transcript AK098131 within intron 3 of *STOX2* (Supplementary File 5).

### A non-coding RNA transcribed from intron 3 of *STOX2* affects alternative splicing of the host gene in placental cells

This raised the question: what is the nature and function of this transcript in relation to the *STOX2* transcription unit? Can mutations or variations be identified in one or both transcripts that could explain the disease either by association with the phenotype and/or by functional evidence?

The annotated transcript of *STOX2* (NM_020225) has four exons with an mRNA size of 10,458 bases and codes for a winged helix domain containing protein of 926 amino acids. Two additional transcripts are associated with *STOX2*, AK001394 (downstream of the last exon) and AK098131 (within intron 3 before the last exon).

By strand-and allele-specific RT-PCR, we proved that the downstream transcript AK001394, rather than being an independent transcript, is part of the *STOX2* transcript forming a large 3′-untranslated region (6243 bp, chr4:184938435-184944677) (GENCODE, ENST00000308497.4) and is expressed from both alleles (data not shown). This extended 3′-untranslated exon was included for sequence analysis.

By strand-specific RT-PCR, the intron 3-associated transcript, AK098131 was found to be expressed in extravillous trophoblast cells (SGHPL5), to consist of a single exon with no coding potential and to have a size of at least 4.7 kb (i.e. larger than AK098131) with transcription in the same direction (sense) as *STOX2* (Supplementary File 6). Using informative combinations of primer sets, including a divergent primer pair, we ruled out that detection of AK098131 was due to unspliced pre-mRNA, lariat intermediates, floating lariats, alternative transcripts or circular RNA (data not shown). cDNA synthesis based on oligodT- and 5′-cap isolation failed, suggesting that AK098131 is transcribed by RNA polymerase III with independent transcription of its host gene or involves the novel mechanism of polyadenylation-independent Pol II transcription termination^11^. In line with current nomenclature for noncoding RNAs, this non-coding lncRNA will be referred to as STOX2-IT3-lncRNA.

In a third sequencing round, we sequenced all exons of *STOX2* (NM_020225) and STOX2-IT3-lncRNA in all individuals from both families with DNA available (n = 26). By this, only a single mutation (CT insertion at chr4:184936601) of paternal origin was found in STOX2-IT3-lncRNA in two individuals (7293, 7301) of family 13. We argued that mutation analysis had remained incomplete when the majority of the mutations are located within genomic regulatory, non-exonic sequences that had not been not selected yet for sequencing. For example, if STOX2-IT3-lncRNA involves a non-coding RNA mediating regulation of epigenetic targets^12^,^13^,^14^,^15^, identification of the target region(s) would be essential with functional exploration of the interactions, if any, preceding further sequencing efforts. A second option could be, as seen for the *STOX1* and *INO80B* genes, that the context-dependent expression of polymorphic variations located in highly conserved regions of the protein or transcript and their interaction with a second gene (*Nodal, miR1324*) are needed for full penetrance of the disease^16^.

We therefore applied *in-vitro* RNAse H-mediated degradation of STOX2-IT3-lncRNA induced by chimeric antisense oligonucleotides in extravillous trophoblast cells (SGHPL5) followed by analysis at the RNA (targeted qRT-PCR, whole genome RNA sequencing) and protein levels (immunocytochemistry, Western blot) of the host gene, *STOX2* (Supplementary Files 7–12)^17^. The combined data showed that extravillous trophoblast cells express three major STOX2 transcripts (Supplementary File 7). These transcripts were identified by whole genome RNA sequencing, confirmed by isoform-specific RT-PCR and correspond to T275439, T275444 and T275446 of the miTranscriptome annotation^18^. They differ in their first exons, generate nuclear proteins of 95.5, 102.6 and 97.3 kDa, respectively and all contain the conserved C-terminal domain reactive with STOX2 antibody PA5-21063 (Supplementary Files 8 and 9). Experimental downregulation of the STOX2-IT3-lncRNA (Supplementary Files 10 and 11A) redirects alternative splicing of *STOX2* with upregulation of transcripts containing an additional third exon (3B) (Supplementary Files 11B-D and 12).

In other words, the default situation i.e. normal function of the STOX2-IT3-lncRNA, is to maintain expression of the full-length STOX2 protein. When the ncRNA is downregulated, a *STOX2* transcript with an alternative third exon 3B, normally present at low levels, becomes upregulated. This alternative exon introduces a stop codon resulting in the deletion of a highly conserved domain of 64 amino acids in the C-terminal of the STOX2 protein.

### STOX2-IT3-lncRNA affects genes associated with trophoblast differentiation and invasion

The above data indicated the presence of a regulatory loop between STOX2-IT3-lncRNA and *STOX2*. To identify additional (in)direct targets controlled by STOX2-IT3-lncRNA, we applied high-resolution genome wide cDNA sequencing of polyA^+^ mRNA obtained from the same series of *in-vitro* antisense experiments. Significant (q < 0.05) differential expression was found for 15 genes after STOX2-IT3-lncRNA downregulation (Table 1). Six (*CEBPA, GADD45G, DLL4, ARC, STK4*-*AS1*, *GNN*) showed log2(fold changes) of −2 > x > 2. CEBPA codes for a trophoblast lineage-specific bZIP transcription factor and is central in a trophectoderm core transcriptional regulatory circuitry^19^,^20^,^21^. CEBPA binding is heterodimer-dependent with CEBPA/CEBPB protein level ratios regulating cell cycle gene batteries^22^. GADD45G is a trophoblast-enriched (highly expressed in the placenta) gene that controls S and G2/M cell-cycle checkpoints^23^. DLL4 is a Notch-ligand with a negative effect on trophoblast invasion^24^. ARC is an activity-regulated cytoskeleton-associated protein^25^. *STK4-AS1* and *GNN* are noncoding RNAs with unknown function. By qRT-PCR, we confirmed the RNA-sequencing results using *GADD45G* and *CEBPA* (Supplementary File 13).

In addition, the RNA sequencing experiments showed that transcript T275448, one of the exon 3B-containing *STOX2* transcripts and reliably identified using annotation-guided transcriptome reconstruction with RABT assembly in the Tuxedo pipeline (Cufflinks), is the predominant transcript that becomes upregulated after STOX2-IT3-lncRNA downregulation.

### Intron 1 of STOX2 contains a second paternal mutation within a candidate superenhancer region

Given the involvement of CEBPA in the *STOX2* pathway, we looked for and sequenced the CEBPA/B binding sites within and upstream of the *STOX2* transcription unit to identify additional mutations. Members of the CEBP family together with GATA members are known for their importance in placental development and trophoblast-specific gene regulation^26^. This binding site guided approach identified a second paternal mutation (G>T) (chr4:184918288) in individuals 7291 and 7298 of family 13 within a binding site for CEBPB/MAFK. This mutation is located in a large region of intron 1 that qualified as a superenhancer (or second promoter) based on histone marks and transcription factor occupancy (Supplementary File 14).

### Non-coding RNA-directed splicing of STOX2 is affected in Finnish pre-eclamptic patients

Although limited to two confirmed mutations, both of paternal origin, and observed in family 13 only, these mutations should have an effect on *STOX2* function if *STOX2* is to be considered as a susceptibility gene for pre-eclampsia in Finnish families. We therefore tested the effects of both mutations on the mechanism of exon 3B inclusion. We simultaneously explored if this mechanism included transcriptional control by a chromatin-splicing adaptor complex as described for the *FGFR2* locus^14^. If such a mechanism operates for *STOX2*, an interaction between the lncRNA and a regulatory genomic region should be present. The additional argument for this decision was the observation that *CHD5*, coding for a chromodomain helicase DNA binding protein, was one of the genes with significant differential expression after STOX2-IT3-lncRNA downregulation (Table 1). Moreover, CHD5 can bind directly to H3K27me3, is predicted (STRING network, score 0.806) to interact with MORF4L1 (MRG15), while chromodomain proteins form chromatin-adaptor proteins^15^,^27^,^28^. Finally, GO analysis of 708 CHD5/H3K27me3 double-positive genes revealed that they are primarily enriched for transcriptional regulators involved in developmental signaling pathways of non-neuronal lineages^27^.

**Figure 2:**
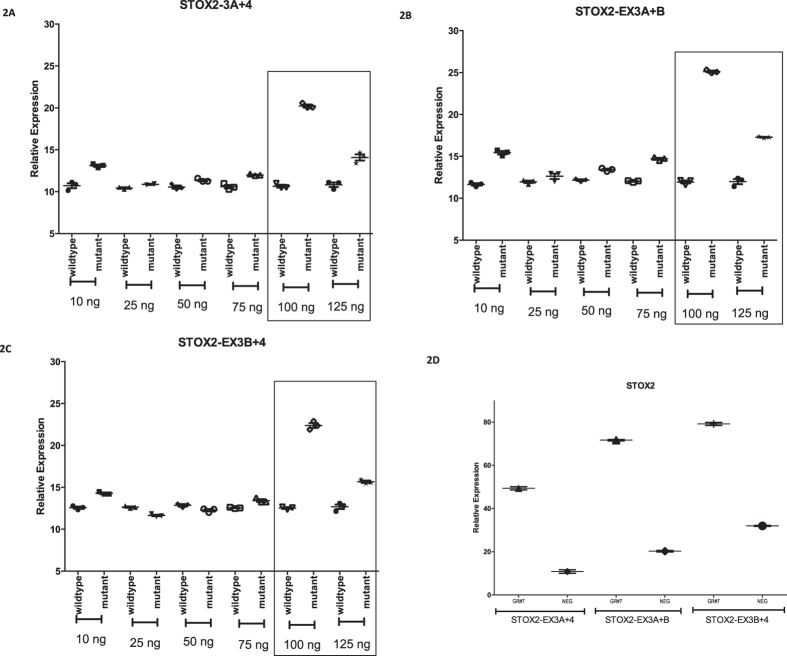
Non-coding RNA-induced gain-of-function of STOX2 in Finnish pre-eclamptic patients. Two mutations present in pre-eclamptic patients were tested for their effect on non-coding RNA-regulated alternative splicing of STOX2. The first mutation is present in the STOX2-IT3-lncRNA and was tested by CMV-promoter driven overexpression of wild-type and mutant constructs. At saturating levels (≥100 ng plasmid) (indicated by boxes), *overexpression* of mutant exogenous STOX2-IT2-lncRNA leads to a gain-of-function with an increase of all three STOX2 transcripts. Isoform-specific qRT-PCR assays were used to differentiate between transcripts without (**A**) or with the alternative exon 3B (**B,C**). Statistical analysis was done using GraphPad Prism 6. The second mutation is present in a genomic region of intron 1 qualifying as a superenhancer or second promoter and was tested by CRISPR assays (**D**). The guide RNA STOX2-gR#7 used for targeting the G>T mutation is marked as GR#7. Compared to negative controls, *underexpression* of normal endogenous STOX2-IT2-lncRNA leads to a gain-of-function with an increase of all three STOX2 transcripts.

We started by testing the paternal CT insertion (insCT) mutation expressed by the lncRNA. Both normal and mutated lncRNAs were overexpressed by transfection with CMV promoter-driven wild-type (659 bp) and mutant (661 bp) fragments of STOX2-IT3-lncRNA in extravillous trophoblast cells (SGHPL5) followed by quantitative transcript analysis of the host gene, *STOX2*. Transcripts were normalized using GAPDH and corrected for effector levels using AK098131. At the two highest doses tested, a gain-of-function was seen for the mutant InsCT construct with increased levels for all three STOX2 transcript variants, i.e. STOX2-EX3A+4, STOX2-EX3A+3B, STOX2-EX3B+4 (Fig. 2A–C).

Secondly, we tested the candidate superenhancer/promoter region within intron 1 with binding sites for CEBPB/MAFK and containing the second paternal mutation (G>T). For this, the CRISPR-Cas9 gene editing system was used as a method to block the genomic target site (Supplementary File 15)^29^,^30^. Out of three guide RNAs tested, the one targeting the mutation site (GR#7) was superior with respect to transfection efficiency, reproducibility and effect. Moreover, this guide RNA was complementary to the Crick strand and as such not reactive with STOX2 precursor RNA assuring that any effect seen operates exclusively at the transcriptional level. With increased levels of guide RNA as monitored by transfection efficiencies (% fluorescent cells), the level of STOX2-IT3-lncRNA, as quantified by qRT-PCR, decreased proportionally. In fact, at efficiency levels of 30% and above, STOX2-IT3-lncRNA became undetectable. This confirmed the existence of an interaction within the STOX2 transcription unit between the regulatory region within intron 1 and the expression of the lncRNA generated from intron 3. At the highest dosage of guide-RNA induced downregulation of STOX2-IT3-lncRNA, the same gain-of-function was observed as for the InsCT mutant lncRNA overexpression described above: upregulation was seen for all three *STOX2* transcript variants (Fig. 2D).

As a final control, given the above data, we rechecked the transcription annotation files generated with RABT assembly of the RNA-sequencing experiments to see if and how all these targeted experiments correlated with the whole-genome data. This not only showed a perfect correlation, but also allowed the following conclusion. The two prevailing transcripts, NM_020225 and T275446, are important for normal trophoblast differentiation. Under experimental conditions, designed to mimick the *in-vivo* situation operating in the placenta of children born from pre-eclamptic females, T275446 gains an additional exon 3B and becomes T275448 encoding a truncated STOX2 variant without the conserved C-terminal domain (Supplementary File 15).

## Discussion

Here we demonstrate that a long non-coding RNA within intron 3 of the *STOX2* gene on 4q35.1 acts as a permissive cis-acting regulator of alternative splicing of *STOX2* and is a candidate gene for pre-eclampsia in Finnish patients. The normal function of the intronic ncRNA, STOX2-IT3-lncRNA, is to maintain expression of the full length protein. When this intronic lncRNA is absent (experimental downregulation) or defective (pre-eclampsia patients), CHD5 is upregulated and a short alternative exon (3B) differentially used in multiple transcripts of STOX2. When this alternative exon is present, a conserved domain of 64 amino acids in the C-terminal region of the STOX2 protein becomes deleted. We postulate that phenotypically, as seen for other pre-eclampsia genes, the same essential process of trophoblast differentiation, where trophoblast cell cycle exit accompanies the transition from proliferative to invasive cells, appears affected by the *STOX2* gene^1^,^2^,^6^.

**Figure 3:**
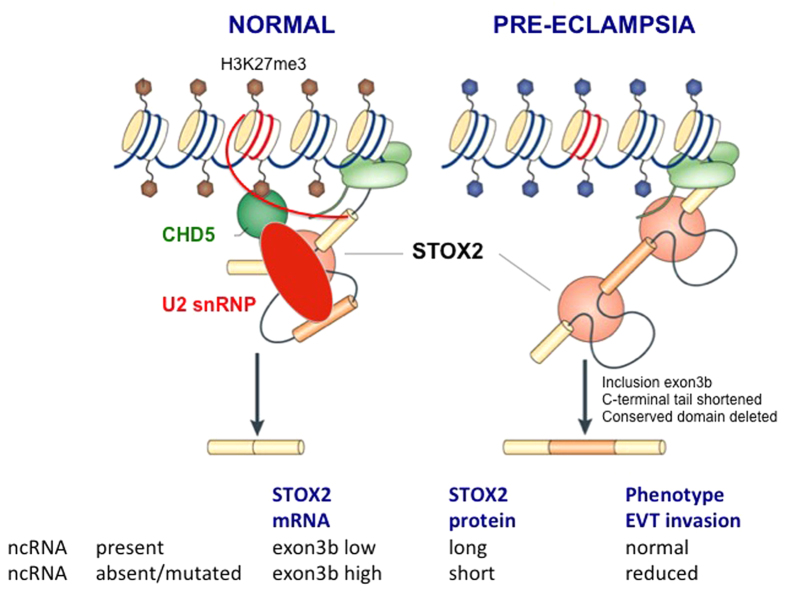
Hypothetical model for the proposed action in the early placenta of STOX2-IT3-lncRNA on alternative splicing of the STOX2 gene in normal and pre-eclamptic pregnancies. Under normal conditions, the lncRNA transcribed from intron 3 of STOX2 (curved red line) forms a chromatin-adaptor splicing complex with CHD5 and U2 snRNP that maintains expression of the full length STOX2 protein by an effect on alternative splicing. In patients with pre-eclampsia, this interaction is defective either by mutations in the lncRNA or in the genomic region interacting with this lncRNA. As a consequence, alternative splicing favors the inclusion of an additional short exon (3B) leading to the deletion of a conserved domain in the C-terminal of the STOX2 protein. This model parallels that of described for the *FGFR2* locus^15^. The different isoforms of the STOX2 protein and the nature of the conserved domain are described in Supplementary File 6.

Based on our mutation data and CRISPR experiments, we postulate that the mechanistic model of Gonzalez described for *FGFR2* can be applied to *STOX2* as well. In the Gonzalez model, the presence of a nuclear antisense lncRNA, generated from within the *FGFR2* locus recruits Polycomb-group proteins (EZH2, Suz12) and a histone demethylase (KDM2a) to create a chromatin environment that impairs binding of a repressive chromatin-splicing adaptor complex (MRG15-PTB). The net effect is inclusion of exon 3B of FGFR2^14^. In our model, the chromatin modifier is most likely CHD5 coding for a chromodomain helicase DNA-binding protein while the histone modification recognized is H3K27me3. DNA binding specificity is controlled by CEBPA and depending on the balance between CEBPA and MAFK hetero- and homodimers, transcription is activated or suppressed. The essential components for this cotranscriptional pre-mRNA splicing are CEBPA-H3K27me3-CHD5. For *STOX2*, the following scenario emerges: when the nuclear *sense* lncRNA generated from within the *STOX2* locus is *absent*, recruitment of the Polycomb-histone demethylase complex is prevented by CHD5 leading to impaired binding of a repressive chromatin-splicing adaptor complex (Fig. 3). The net effect is inclusion of exon 3B of STOX2. Both models imply involvement of polycomb-group proteins (EZH2 in both models) as well as enhancer binding proteins affecting RNA polymerase II activity (CEBPA in our model).

We conclude that two options remain to deliver final proof that *STOX2* causes pre-eclampsia in Finnish pre-eclamptic females.

The novel regulatory mechanism involving chromatin-splicing adaptor complexes can be used to succesfully guide the identification of the remaining mutations that could reside in the 40,496 bp fragment (chr4:184,882,511-184,932,935) within intron 1 of *STOX2* (Supplementary File 17). This is based upon the following arguments: i. The downstream part of this region qualifies as an intragenic (super)enhancer or second promoter, ii. is strategically positioned before exon 2 that is shared by all *STOX2* transcripts, iii. contains multiple binding sites for CEBPB and contains at least one mutation with an effect on *STOX2* splicing through interaction with the lncRNA of intron 3. This scenario does not rule out that other, more upstream regions are involved. These ‘faraway’ regions could be identified by powerful yet elaborate methods (MPRA screen) as recently described for the identification of the distant (12 kb upstream) trophoblast-specific enhancer that controls HLA-G expression at the maternal-fetal interface^26^.

The second scenario is that, as for *STOX1* and *INO80B*, the genomic variations that cause the disease are polymorphic variations rather than classical mutations, but only cause disease when expressed in a specific combinatorial context while the variations are located in highly conserved regions^1^,^16^. In this scenario, the eight variations present in STOX2-IT3-lncRNA and responsible for the hotspot that segregates with all affected mother-child combinations could qualify as such.

For both scenarios, the essential interactions are the ones that act *in-cis.* The experiments designed to test this should be likewise. In this respect, all the experiments we performed with inhibition of the endogenous target (lncRNA in intron 3 or genomic region in intron 1) (favoring inhibition of *in-cis* interactions) were consistent with each other and confirmed that STOX2-IT3-lncRA acts as a permissive cis-acting regulator of STOX2 alternative splicing.

In conclusion, the *STOX2* gene qualifies as the fourth susceptibility pre-eclampsia gene that affects the same essential process of extravillous trophoblast differentiation responsible for the primary defect in early-onset pre-eclampsia and related syndromes.

## Materials and Methods

### Mutation analysis

Genomic DNA was isolated from EDTA blood of 26 individuals from 4 generations of 2 Finnish families (family 7 and family 13) with demonstrated linkage to chromosome 4^8^,^9^. Study and experimental protocols approval and informed consents were as described^9^. Methods were carried out in accordance with the relevant guidelines. All experimental protocols were approved and accredited (NEN-EN-ISO-15189:2012) by the RvA-Unit Zorg (CCKL), Utrecht, the Netherlands. Written informed consent was obtained from all subjects. The pedigree structures with the individuals tested are shown in Supplementary File 2. The pedigree structures have been updated and corrected in comparison to the original pedigree structures^8^,^9^. The population tested shared the same clinical features with the other populations with familial pre-eclampsia. Two prominent clinical features are common i.e. early-onset (symptoms before week 34), and frequent (40–50%) association with intra-uterine growth-restriction. Genomic DNA was subjected to whole genome amplification (WGA) according to the manufacturers instructions (Qiagen) and checked for the absence of allele drop-out using a set of informative SNPs. Sequencing primers were designed using ExonPrimer and used for mutation analysis by Sanger sequencing. ExonPrimer was used with default settings including masking against hg18 (score 300) except that the GC clamp option was not used. Primer sequences are available upon request. Sequence files (.abl) were analyzed using 4Peaks, and aligned using Sequencher v5.0.1.

### Haplotype analysis

Sequence variants identified were verified for their chromosomal position (UCSC hg19), screened for their class, function, molecule type, heterozygosity and allele frequencies (dbSNP 142) and scored for maximal allele sharing between affected sibs. The minimal critical region was defined as the region with the greatest identity-by-descent allele-sharing (in affected sibs) or the solid spine of LD with identical flanking minor alleles. Variants were considered mutations when absent in 100 normal controls (200 chromosomes).

### RNA expression analysis

Strand-specific RT-PCR, either alone or combined with allele-specific RT-PCR using total RNA of extravillous trophoblast (SGHPL5) was done as described^1^,^2^.

### Functional *in vitro* analysis

*In vitro* inhibition studies in extravillous trophoblast cells (SGHPL-5) were done using antisense oligos (ASO). The oligo modifications were as described with chimeric antisense oligos containing nuclease resistant domains at both ends (2′-O-methoxyethyl ribonucleotides) and a central core of phosphorothioate-modified desoxynucleotides^17^. Upon complementary binding with target RNA, RNase H-mediated degradation of the double-stranded antisense oligo:RNA hybrid is induced. Target site selection was performed using the BLOCK-iT-RNAi program followed by BLAST analysis to check for specificity and to confirm the absence of SNPs in the sequences targeted. For AK098131 inhibition, ASO-AK (5′-mA* mA* mC* mA* mU* mG* G* C* A* C* A* T* G* C* T* T* T* rG* mA* mU* mC* mU* mA-3′ (23 nt, 36%GC) (100 nmol) (HPLC RNAse-free) complementary to chr4: 184933185-184933207 (hg19) was used. Using RNAplfold implemented in RegRNA 2.0, the region targeted qualifies as an accessible region. As a negative control, ASO-INTRON3 (5′-mA* mA* mU* mU* mU* mG* A* G* G* T* G* A* T* A* G* C* T* A* G* mC* mU* mA* mU* mC* mA-3′) (25 nt, 36%GC) (100 nmol) (HPLC RNAse-free) was used. This oligo is antisense to chr4:184937924-184937948 (hg19) and located within intron 3 but outside AK098131.

For transfection, near confluent SGHPL-5 cells were harvested, counted using the Countess cell counter and plated in 12 well plates with 200,000 cells per well in Iscove’s complete medium supplemented with 10% FBS and pen/strep. On day 2, cells (60-80% confluency) were transfected with 15, 30, 50 or 60 pmol of ASO oligonucleotides using FuGene HD in complete medium. All reactions were done in triplicate. The effects of AK098131 inhibition were tested in multiple ways. Differential RNA expression analysis was done by targeted transcript quantification as well by whole genome RNA sequencing. For both, total RNA was isolated 48 hours after transfection using RNABee and affinity isolation using Qiagen columns according to the manufacturers instructions. Transcript analysis was done by quantitative RT-PCR with the TaqMan RNA-to-Ct 1-step kit (Life Technologies). Transcripts targeted (and assays used) were AK098131 (Hs03870651_s1), STOX2 (Hs01391761_m1, Hs00858930_m1, Hs01391114_m1), GAPDH (Hs02758991_g1), CEBPA (Hs00269972-s1) and GADD45G (Hs02566147-s1). Quantitative analysis of T275448, T275446, T275429, T275439, T2275445, T275450 and T275451 (numbers correspond to miTranscriptome database) was done with PrimeTime qPCR assays (IDT) using Taqman probe-intron spanning primer combinations designed using PrimerQuest (IDT). All qRT-PCR reactions from at least 3 independent experiments were done in duplicate and performed for all concentrations of inhibitors used (15-30-50-60 pmol). For genome-wide RNA sequencing, total RNA was isolated from SGHPL5 cells subjected to conditions of optimal inhibition of AK098131 (50 pmol ASO-AK) or no inhibition (50 pmol ASO-INTRON3). Prior to cDNA library synthesis, downregulation of AK098131 was confirmed by qRT-PCR as above. PolyA+ RNA isolation and cDNA library synthesis were done using the TruSeq RNA sample preparation kit version 2 (Illumina). cBot clustering and paired-end (2× 125 bp) sequencing was done on a HiSeq2500 with version 4 chemistry. Total number of reads per sample was around 600 million with mean Q values > 35. For differential expression analysis, the Tuxedo pipeline was used (Tophat-2.0.14, Cufflinks-2.2.1) with annotation guidance using the Illumina UCSC hg19 iGenome.

### CRISPR-Cas mediated editing of STOX2

#### Target site design

A 100 bp genomic STOX2 fragment (chr4:184,918,238-184,918,337) with the nucleotide (chr4:184,918,288) of the second paternal mutation (G>T) positioned in the centre was used to identify guide RNA binding sites with NGG protospacer adjoining motifs (crispr.mit.edu)^29^. Three guide RNA target sites were selected. Guide RNA target sites and predicted double strand break sites are depicted in Supplementary File 15. The guide RNA STOX2-gR#7 targeting the G>T mutation was used in truncated form (18nt) to reduced undesired off-target effects^30^.

#### Cloning in GeneArt CRISPR nuclease OFP reporter vector

Cloning of guide RNA sequences into GeneArt CRISPR nuclease vector as described by the manufacturer (Life Technologies). Complementary oligonucleotides were annealed to create double stranded DNA for vector ligation: STOX2-gR#1a 5′-GCA TGT GCG TTT GGT TCA CGT TTT-3′ and STOX2-gR#1b 5′-GTG AAC CAA ACG CAC ATG CCG GTG-3′; STOX2-gR#2a 5′-GAC AGT AAA CTC GGC TGT GAG TTT T-3′ and STOX2-gR#2b 5′-TCA CAG CCG AGT TTA CTG TCC GGT G-3′; STOX2-gR#7c 5′-GCG CAC ATG CAC ACA TGC AGT TTT-3′ and STOX2-gR#7d 5′-TGC ATG TGT GCA TGT GCG CCG GTG-3′. Following ligation and transformation, correct constructs were identified by PCR and confirmed by sequencing.

#### Transfection

For transfection, SGHPL-5 cells were harvested at 70–90% confluency, counted using Countess cell counter and plated in 48 wells plates with 30,000 cells/well in 250 μl Iscove’s medium with 10% FBS and penicillin/streptomycin (complete medium). At day 1, to a sterile tube, the required amount of prewarmed medium (without FBS and without P/S) and DNA (from 100 ng/μl stock) were added and vortexed. FuGene HD was added directly, mixed (final volume 15 μl) and incubated for 15 min at RT. To each reagent/DNA mixture, 235 μl of complete medium was added, and mixed thoroughly, but gently followed by replacement of medium in well by 250 μl of the medium/FuGene/DNA mixture. After incubation of cells for 48 hours, cells were harvested and scored for efficiency by OFP analysis using fluoresence microscopy and scored for genome editing by DNA sequencing. qRT-PCR analysis was done as above. At a pre-defined effect size (fold change of 3) and a maximum CV of 25%, the number of independent replicates needed and used was 3. All transfection experiments were done in triplicate. All RT-PCR assays from at least 3 independent experiments were done in duplicate. Statistical analysis was done using GraphPad Prism version 6.0. Results are expressed as mean with SD.

#### Immunocytochemistry

For immunofluoresence staining, 35,000 SGHPL-5 cells were plated in IMDM growth medium containing 10% fetal bovine serum with pen/strep in a 8-well Nunc Lab-Tek II Chamber Slide system. Twenty-four hours post-seeding, the cells were transfected with 50 μM of AK098131 or INTRON 3 siRNA using FuGENE HD Transfection Reagent according to the manufacturers instructions (Promega). Forty-eight hours post-transfection, the culture medium is removed, washed and the transfected cell chambered slides were fixed with freshly prepared 4% (w/v) paraformaldehyde in 1 × PBS with 1% Triton X-100 for 10 minutes. The slides were washed in wash buffer (1 × PBS, 0.5% Triton X-100) three times; ten minutes each wash and then blocked in blocking buffer (1 × PBS, 10% normal goat serum, 0.1% blocking reagent (Roche), 0.5% Triton X-100) for 1 hour followed by incubation in blocking buffer with rabbit polyclonal anti-STOX2 antibody (Thermo scientific; PA5-21063; 1:700) at room temperature for 1 hour. Incubation was followed by washing in wash buffer three times; ten minutes each wash. The slides were then incubated in blocking buffer containing Alexa Fluor 568 goat anti-rabbit secondary antibody (Invitrogen; A11011; 1:300) for 30 minutes at room temperature. Slides were washed in wash buffer three times; ten minutes each wash, dehydrated in ethanol and mounted in Vectashield containing DAPI nuclear stain (Vector Laboratories). Visualization was performed on a Leica DM5000B microscope.

### Protein isolation, subcellular fractionation, and Western blotting

Protein lysates were obtained from the SGHPL5 cells after transient transfection with AK098131 and INTRON3 siRNA. For nuclear/cytoplasmic fractionation, cultured cells were fractionated into nuclear and cytoplasmic lysates using the PARIS kit from Ambion following the manufacturer’s instructions. Downregulation of STOX2 was assessed in duplicate by quantitative RT-PCR with the Taqman Gene Expression Assay (Hs01391761_m1, Applied Biosystems) on RNA (1 μl) after AK098131 and INTRON3 siRNA knockdown using the Taqman RNA-to-Ct Kit (Applied Biosystems). For western blot analysis of protein lysates, the gel was loaded with 10 μl protein ladder (10–250 kDa; Precision Plus Protein Standard (Bio-Rad)) as a protein size marker, followed by the protein samples (30ug) determined by the Bradford assay (Bio-Rad). The proteins were separated by SDS-polyacrylamide gel electrophoresis, and electroblotted onto a PVDF-membrane. Membranes were blocked with blocking buffer (1 × PBS, 5% (w/v) milk powder, 0.5% Tween 20) for 30 minutes at room temperature. After blocking, membranes were incubated with a rabbit polyclonal STOX2 antibody (Thermo Scientific; PA5-21063; 1:500) or a rabbit polyclonal acetylated histone H3 antibody (Upstate; 06-599; 1:1000) in blocking buffer, overnight at 4 °C on a rocker, then washed five times and followed with an HRP conjugated goat anti-rabbit polyclonal secondary antibody (Dako; P0448; 1:2000) in blocking buffer for 1 hour. This was followed by three washes for 5 minutes in wash buffer at room temperature. Bands were visualized using the ECL Western Blotting Detection System (GE Healthcare, UK) for 5 minutes prior to image acquisition by ChemiDoc MP Imager (Bio-Rad).

## Additional Information

**How to cite this article**: Oudejans, C. B.M. *et al.* Noncoding RNA-regulated gain-of-function of *STOX2* in Finnish pre-eclamptic families. *Sci. Rep.* **6**, 32129; doi: 10.1038/srep32129 (2016).

## Supplementary Material

Supplementary Information

## Figures and Tables

**Table 1:** Differentially-expressed genes after downregulation of STOX2-IT3-lncRNA.

#	gene	Differentially-expressed genes after downregulation of STOX2-IT3-lncRNA	locus (hg19)	Q1	Q2	log2(FC)	test_stat	p_value	q_value
1	CEBPA	CCAAT/enhancer binding protein (C/EBP), alpha (CEBPA	chr19:33790839-33793470	0.791784	0.0983437	−3.0092	−7.27434	5.00E-05	0.0448139
2	DLL4	Delta-like 4 (Drosophila) (DLL4)	chr15:41221530-41231258	1.43608	0.210301	−2.7716	−7.42592	5.00E-05	0.0448139
3	GADD45G	Growth arrest and DNA-damage-inducible, gamma (GADD45G)	chr9:92219926-92221469	5.43987	1.0088	−2.43093	−8.6197	5.00E-05	0.0448139
4	STK4-AS1	STK4 antisense RNA 1 (head to head) (STK4-AS1), non-coding RNA	chr20:43592439-43595099	1.00836	0.213612	−2.23894	−5.35714	5.00E-05	0.0448139
5	ARC	Activity-regulated cytoskeleton-associated protein (ARC)	chr8:143692404-143695833	2.00488	0.450934	−2.15253	−6.63099	5.00E-05	0.0448139
6	RASD1	RAS, dexamethasone-induced 1 (RASD1), transcript variant 1	chr17:17397752-17399709	1.92755	0.512219	−1.91194	−4.70234	5.00E-05	0.0448139
7	FAM222A	Family with sequence similarity 222, member A (FAM222A)	chr12:110152186-110211292	2.04448	0.612799	−1.73825	−6.32715	5.00E-05	0.0448139
8	RND1	Rho family GTPase 1 (RND1)	chr12:49250915-49259653	3.7851	1.4777	−1.35697	−4.54979	5.00E-05	0.0448139
9	GOLGA6L10	multiple genes	chr15:82632349-83195272	330.041	150.017	−1.13752	−5.6044	5.00E-05	0.0448139
10	RPS17	Ribosomal protein S17-like (RPS17L)	chr15:83205500-83209295	339.472	156.56	−1.11657	−5.58856	5.00E-05	0.0448139
11	MMP1	Matrix metallopeptidase 1 (MMP1), transcript variant 1	chr11:102654406-102714342	302.587	165.872	0.867278	−4.12499	5.00E-05	0.0448139
12	PYGM	Phosphorylase, glycogen, muscle (PYGM), transcript variant 1	chr11:64513860-64528187	1.09367	2.79179	1.35201	5.11369	5.00E-05	0.0448139
13	FAM83E	Family with sequence similarity 83, member E (FAM83E)	chr19:49103856-49116694	0.956191	2.96315	1.63176	5.63033	5.00E-05	0.0448139
14	CHD5	Chromodomain helicase DNA binding protein 5 (CHD5)	chr1:6161846-6240194	0.132269	0.410489	1.63386	4.44095	5.00E-05	0.0448139
15	GNN	Grp94 neighboring nucleotidase pseudogene (GNN), non-coding RNA	chr12:104237526-104323989	0.425877	1.99512	2.22797	7.16115	5.00E-05	0.0448139

Total RNA was isolated from SGHPL5 cells subjected to conditions of optimal inhibition of AK098131 (50 pmol ASO-AK) or no inhibition (50 pmol ASO-INTRON3). Prior to cDNA library synthesis, downregulation of AK098131 was confirmed by qRT-PCR. PolyA+ RNA isolation and cDNA library synthesis were done using the TruSeq RNA sample preparation kit version 2 (Illumina). cBot clustering and paired-end (2× 125 bp) sequencing was done on a HiSeq2500 with version 4 chemistry. Total number of reads per sample was around 600 million with mean Q values > 35. For differential expression analysis, the Tuxedo pipeline was used (Tophat-2.0.14, Cufflinks-2.2.1) with annotation guidance using the Illumina UCSC hg19 iGenome. The genes with significant differential expression are listed. Eleven genes are downregulated following specific inhibition against AK098131 (Q1 value = normal FPKM values, Q2 value = FPKM values after inhibition). Four genes are upregulated.
