# Supplementary material for: Noncoding RNA-regulated gain-of-function of STOX2 in Finnish pre-eclamptic families
Source: Sci Rep. 2016 Aug 24;6:32129. doi: 10.1038/srep32129 (PMC4995371; doi:10.1038/srep32129)
Supplement: Supplementary Information [file srep32129-s1.pdf]

Noncoding RNA-regulated gain-of-function of *STOX2* in Finnish pre-eclamptic families

Cees BM Oudejans[1*], Ankie Poutsma[1], Omar J Michel[1], Hari K Thulluru[1], Joyce Mulders[1], Henri J van de Vrugt[2], Erik A Sistermans[2], Marie van Dijk[1]

[1]Department of Clinical Chemistry, VU University Medical Center, Amsterdam, the Netherlands; [2]Department of Clinical Genetics, VU University Medical Center, Amsterdam, The Netherlands.

Legends to the Supplementary Files

Supplementary File 1. Placental model of genetic forms of early-onset pre-eclampsia. Placental gene defects in the extravillous trophoblast cause a vessel defect in the maternal spiral arteries during the establishment of a connection between the maternal and fetal circulations (1st trimester). Initially, the placenta tries to compensate the deprivation of nutrients by the release of vaso-active factors (PlGF, sFLT, sENG) by inducing TNF-ß mediated NOS-dependent vasodilation in the mother (2nd trimester). This compensation becomes limited when the help requested by the growing fetus becomes in conflict with the metabolic needs of the mother. At this stage, the compensatory mechanisms operating in the mother induce vessel damage and the syndrome becomes symptomatic with maternal hypertension and proteinuria (3rd trimester). The gene defects involved are founder-dependent with different, yet paralogous susceptiblity genes in different populations, and disrupt a common pathway essential for placentation. The colour figure (upper left) was taken from Moffett-King A. Natural killer cells and pregnancy. Nat Rev Immunol. 2002 Sep;2(9):656-63.

Supplementary File 2. Pedigree structures of two Finnish pre-eclamptic families (families 7 and 13) with confirmed linkage to chromosome 4 and used in the present study. Black circles indicate (pre)eclamptic women. White circles indicate unaffected pregnancies. The symbol in individual PEK.7302 indicates gestational hypertension. Grey circles or boxes indicate status unknown or uncertain. Red arrows indicate the children born from affected pregnancies. Irrespective of the mode of inheritance (recessive, dominant, parental effect), an absolute requirement for the placental model (fetal genotype induces the maternal phenotype) is that the risk allele and associated risk haplotype introduced in both families (1A: family 13; 1B: family 7) is shared between all individuals marked with an red asterisk (*).

Supplementary File 3. Reanalysis of the identity-by-descent allele-sharing pattern in the pedigrees of two informative families (7 and 13) with confirmed linkage to 4q using the panel of original microsatellite markers (n=16) in the two regions with the highest

lod scores (D4S1572 and D4S413). Both models were considered: a *maternal* effect gene operating in the mother and a *fetal* effect gene operating in the placenta. In the maternal model, both alleles need to be shared between the affected sisters (7171, 7174) of family 7 and one risk allele present in family 7 shared with the affected sister (7296) from family 13. In the fetal model, both alleles need to be shared between the children born from affected pregnancies (7303 and 7305 in family 13, 7176 and 7177 in family 7). In addition, sharing of only one allele (carrier) is allowed in the child born from a normal pregnancy (7178 in family 7). One region (red box) qualified as the region with the highest numbers of markers shared that fullfilled all of the above criteria and indicated autosomal recessive inheritance of a placentally-expressed gene located within locus 2 near markers D4S1597, D4S1539 and D4S415 (grey). In other words, the region with the highest lod score previously identified by considering a maternal effect gene using all families fully qualified for a placental effect gene when selected for families with linkage to 4q. Blue: non-affected fathers; orange: carrier mothers; red: children born from affected pregnancies; green: children born from normal pregnancies; yellow: regions with maximal allele-sharing; box: microsatellite markers with confirmed lod scores.

Supplementary File 4. Within the 2 loci linked with pre-eclampsia in Finland, 73 SNPs and IN/DEL markers were identified by sequence analysis of the coding regions of six candidate genes in these regions. The candidate genes (locus 1: *CENPE, TACR3, CXXC4, TET2;* locus 2*: CDKN2AIP, STOX2*) had been prioritized using the selection method of pathway-guided genome-wide meta-analysis. Two minimal critical regions (MCR) were found: MCR1 in the *CENPE* gene of the *TET2-TACR3* region and MCR2 in the *STOX2* gene of the *STOX2-DDX60* region. Minor alleles are indicated in grey. Regions identical by descent are marked in yellow. Position of microsatellite markers and associated lod score are marked by arrows. Individuals correspond to those in **Supplementary File 2.**

Supplementary File 5. Irrespective of the mode of inheritance (recessive, dominant, parent-of-origin effect), an absolute requirement for the placental model (fetal effect gene operating in the placenta) is that the risk allele and associated haplotype are

shared between all mother-child combinations when the pre-eclamptic mother carried this child (**Supplementary File 2**). The hotspot identified in this way corresponds to MCR 2 found in the previous analysis (**Supplementary File 4**). The risk haplotype was defined by the minor alleles of 8 SNPs and covered AK098131. Importantly, in the majority (10/16) (62.5%), this included sharing of the minor alleles inherited from the unrelated fathers. The latter effectively rules out that the pattern observed was due to false-positivity caused by the family relatedness of the individuals analyzed. Individuals correspond to those in **Supplementary File 2**. Orange: carrier mothers; red: children born from affected pregnancies.

Supplementary File 6. Within intron 3 (box in **A**) of STOX2, by strand-specific RT-PCR with the primer combinations indicated (**B**), the AK098131 transcript was confirmed to be expressed in extravillus trophoblast and transcribed in the same direction (sense) as STOX2 (**C**). It should be noted that this intron 3 transcript in the extravillous trophoblast is larger than AK098131 (2337 bp). By using a strand-specific RT-PCR with the forward primer of primer set 2 and the reverse primer of primer set 4 we confirmed that the STOX2-IT3-lncRNA is at least 4.7 kb in length. The position of exon 3B is indicated by an arrow.

Supplementary File 7. The transcriptional organisation of STOX2 as predicted by MiTranscriptome (**A**), GENCODE (**B**), and RefSeq (**C**) in comparison to the normal expression pattern in extravillous trophoblast cells (**D**).

Supplementary File 8. Proteins alignments of STOX2 isoforms encoded by transcripts T275446, T275439 and T274444 (MiTranscriptome database).

Supplementary File 9. Under normal conditions (intron 3), three protein bands within the 95-103 kd range and limited to the nuclear fraction were seen. After inhibition of AK098131 (STOX2-IT3-lncRNA), this signal is lost. The position of the 100 kd marker is indicated. The lower panel shows the reaction for the positive control: histone H3. In the panels of Supplementary Files 9, 10 and 11, INTRON3 refers to the negative control.

This oligo is antisense to chr4:184937924-184937948 (hg19) and targets a region located within intron 3, but outside STOX2-IT3-lncRNA. AK098131 refers to the oligo used for specific inhibition of STOX2-IT3-lncRNA and is complementary to chr4: 184933185-184933207 (hg19). Further details can be found in the Materials and Methods section.

Supplementary File 10. Under normal conditions (intron 3), brightly fluorescent nuclear signals representative of nuclear speckles were seen in SGHPL5 cells after immunostaining with a STOX2 antibody (PA5-21063) reactive with a 19 amino acid peptide near the carboxyterminal. After inhibition of AK098131 (STOX2-IT3-lncRNA), this signal is reduced. Red: STOX2; Blue: DAPI counterstaining.

Supplementary File 11. For RNase-H-mediated downregulation, extravillous trophoblast cells (SGHPL5) were transfected with chimeric antisense oligonucleotides against intron 3 (negative control) and against AK098131 (target) followed by q-RT-PCR analysis of AK098131 (target) and STOX2 (effector) transcripts 48 hours after transfection. Dose-dependent inhibition of AK098131 (**A**) was found to correlate with upregulation of the STOX2 transcripts containing the alternative exon (3B) (**C, D**). The false-negative reaction seen in B is caused by a discrepancy between primer versus probe reactivity of the Taqman assay used as explained in **Supplementary File 12.** The optimal dosage (50 ng) is indicated by boxes. Statistical analysis was done by two-way ANOVA using GraphPad Prism 6.

Supplementary File 12. Inspection by gelelectrophoresis of the RT-PCR fragments generated by assay Hs013911761_m1 (spanning exons 3 and 4 of STOX2) of the experiments described in **Supplementary File 11B** confirmed the presence of alternative transcripts. One of these (137 bp) corresponds to the transcript containing the short (49 bp) alternative exon (3B) and was confirmed by sequencing. This transcript reacts with the primers present in Taqman assay (Hs013911761_m1) but is unreactive with the Taqman probe. This explains the false-negative reaction seen in **Supplementary File 11B.**

Supplementary File 13. Confirmation by qRT-PCR of differential expression (downregulation) of CEBPA and GADD45G after dose-dependent inhibition of AK098131 (STOX2-IT3-lncRNA). Statistical analysis was done by two-way ANOVA using GraphPad Prism 6.

Supplementary File 14. The STOX2 transcription unit (chr4:184,882,511-184,932,935) was tested for STOX2-IT3-lncRNA DNA binding motifs and binding sites via Hoogsteen base pairing (**A**), visualized for the presence of sequence variants influencing human transcription factor occupancy in vivo (**B**), histone marks (**C**) and conserved transcription factor binding sites (**D**). The candidate (super)enhancer region is boxed. The paternal mutation tested in the CRISPR assay (**Supplementary File 15**) is indicated with a red arrow.

Supplementary File 15. The CRISPR assay was used as a tool to specifically block the genomic region in intron 1 of STOX2 following cloning of guide RNAs in the CRISPR nuclease OFP reporter vector, inserts confirmed by sequencing, and used for transfection of SGHPL5 cells with transfection efficiencies monitored by OFP positivity. Further details are explained in **Supplementary File 15** itself.

Supplementary File 16. Annotation files (.gtf) from the RNA sequencing experiments representative of normal and disease conditions were generated by Cufflinks with RABT assembly, and uploaded in UCSC browser (hg19) for visualization. Under normal conditions two transcripts prevail: NM_020225 and T275446. Under disease conditions, the latter becomes T275448 by the inclusion of an additional exon 3 (3b).

Supplementary File 17. Detailled genotypes of the *STOX2* region in families 7 and 13. Legends are as in **Supplementary File 3**. Intron 1 is marked in blue. The mutations tested are indicated in red. Other variations confirmed to be absent in normal controls (200 chromosomes tested), but not tested in functional assays are indicated in orange.

Suppl File 1

The placental genotype controls the maternal phenotype

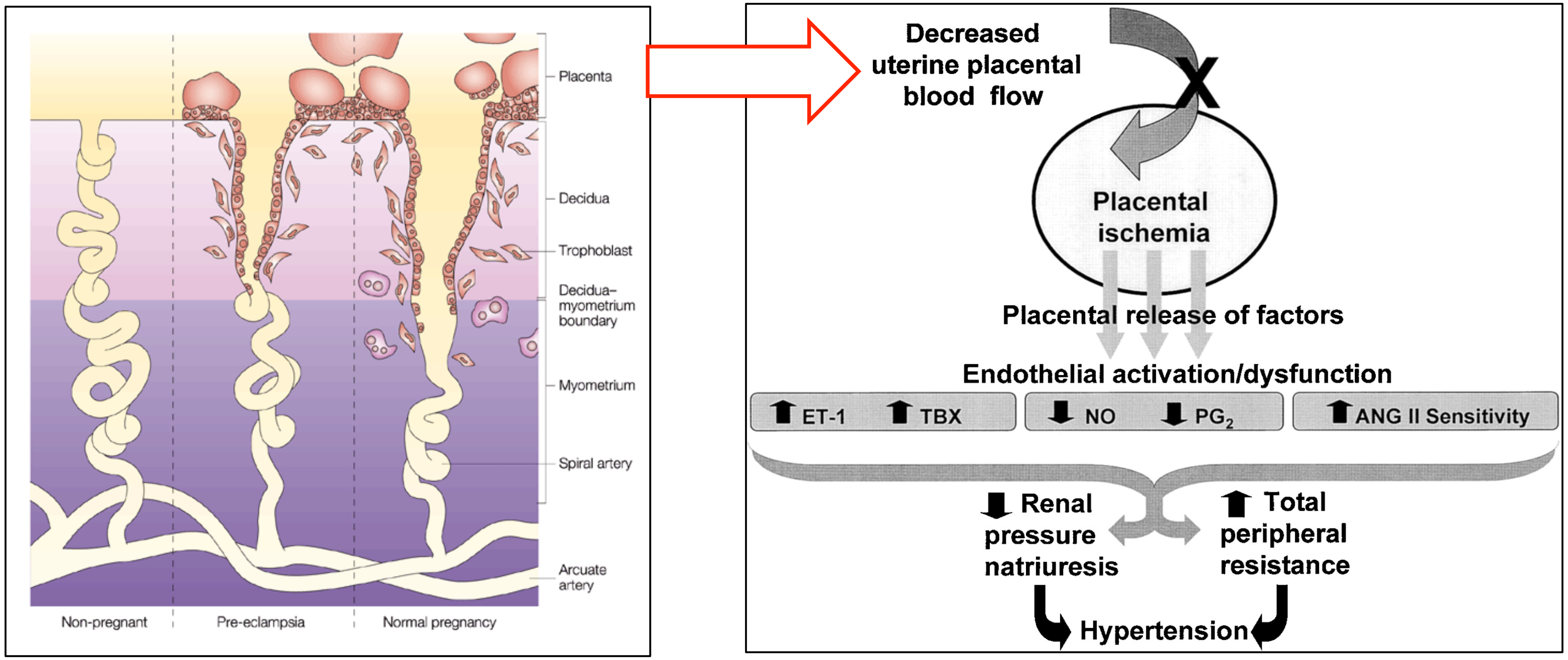

Trimester	1st	2nd	3rd
	Induction	Compensation	Decompensation
Fetal	Placental gene defect	PlGF, sFlt, sEng	
Maternal	Vessel defect	TNF-β NOS-dilatation	Vessel damage<br>Hypertension<br>Proteinuria<br>= Pre-eclampsia

Suppl File 2A

FAMILY 13

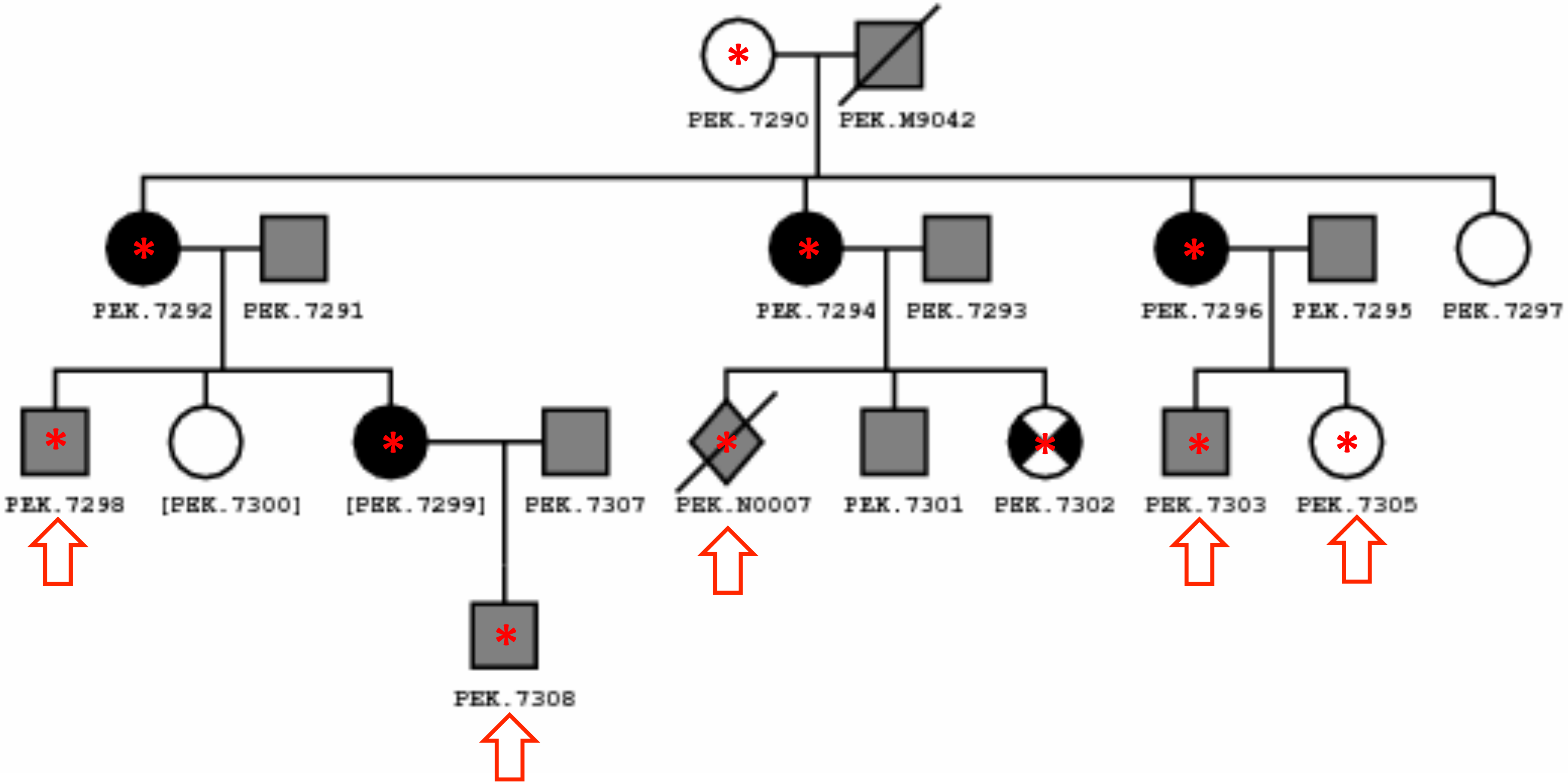

Suppl File 2B

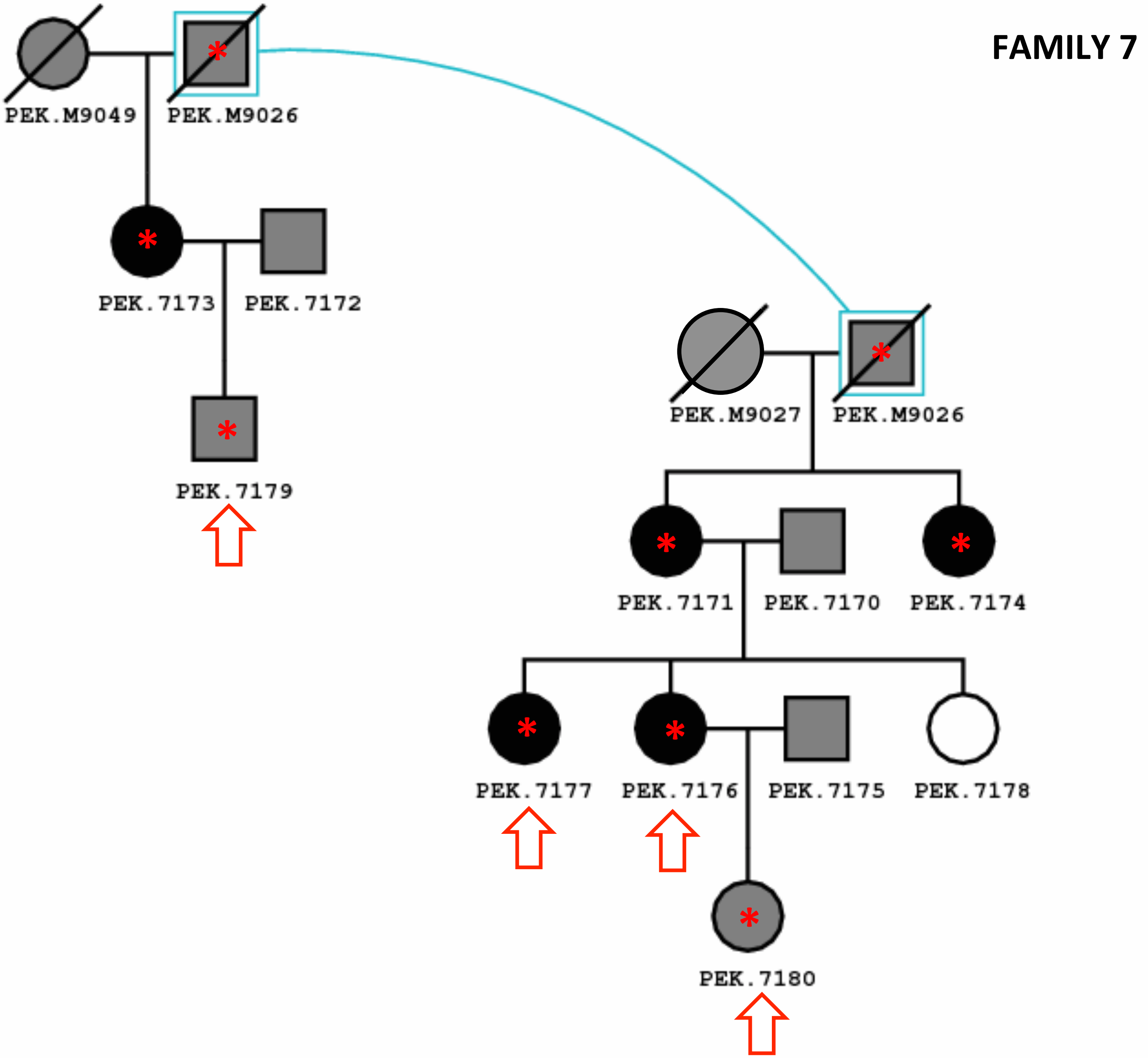

Suppl File 3

MATERNAL

#	Marker	Position	hg19	13		13		7		7		7	
				7295		7296		7170		7171		7174	
1	D4S412	4p16	chr4:3380692	3	6	6	4	4	6	3	4	3	4
2	D4S403	4p15	chr4:13750828	5	7	4	7	1	5	3	6	1	6
3	D4S419	4p15	chr4:18848760	2	5	2	2	1	2	2	6	2	2
4	D4S391	4p15	chr4:27612233	10	11	5	11	4	11	2	9	3	9
5	D4S405	4p14	chr4:40352512	3	9	5	5	3	6	7	7	5	8
6	D4S1592	4q12	chr4:57682157	1	4	8	8	4	6	6	7	4	5
7	D4S392	4q13	chr4:70523293	0	0	1	6	2	6	6	7	6	7
8	D4S2964	4q21	chr4:80775625	2	7	1	2	1	2	1	2	1	4
9	D4S1534	4q21	chr4:86308292	2	2	4	10	2	7	7	10	3	7
10	D4S1572	4q24	chr4:103769921	3	4	7	1	7	7	1	9	1	9
11	D4S1575	4q28	chr4:134790737	0	0	2	2	2	3	1	2	2	2
12	D4S1579	4q31	chr4:140730878	1	1	3	5	5	5	1	5	4	5
13	D4S424	4q31	chr4:142197646	2	2	3	7	7	7	6	7	7	8
14	D4S2962	4q31	chr4:150364337	4	5	2	6	2	5	4	4	4	4
15	D4S413	4q32	chr4:158353155	1	1	9	9	1	9	9	15	9	15
16	D4S3046	4q32	chr4:163390792	4	5	2	3	2	5	2	5	2	3
17	D4S1597	4q32	chr4:169843115	2	2	8	1	5	8	1	2	1	2
18	D4S1539	4q34	chr4:175688140	2	3	1	2	1	1	2	3	2	3
19	D4S415	4q34	chr4:178711101	1	9	1	10	8	9	10	13	10	13

FETAL

#	Marker	Position	hg19	13		13		7		7		7	
				7303		7305		7176		7177		7178	
1	D4S412	4p16	chr4:3380692	6	6	6	6	4	4	4	6	4	6
2	D4S403	4p15	chr4:13750828	7	7	4	7	1	6	5	6	5	6
3	D4S419	4p15	chr4:18848760	2	5	2	2	2	2	2	2	1	6
4	D4S391	4p15	chr4:27612233	10	11	5	11	4	9	4	9	2	4
5	D4S405	4p14	chr4:40352512	3	5	5	9	6	7	6	7	6	7
6	D4S1592	4q12	chr4:57682157	1	8	4	8	6	6	6	6	6	7
7	D4S392	4q13	chr4:70523293	1	5	1	1	2	7	2	7	2	6
8	D4S2964	4q21	chr4:80775625	1	7	1	2	1	2	1	2	1	1
9	D4S1534	4q21	chr4:86308292	2	10	2	10	2	10	7	10	7	7
10	D4S1572	4q24	chr4:103769921	4	7	4	1	7	1	7	1	7	9
11	D4S1575	4q28	chr4:134790737	2	2	2	2	1	3	1	3	2	3
12	D4S1579	4q31	chr4:140730878	1	5	1	5	1	5	1	5	5	5
13	D4S424	4q31	chr4:142197646	2	3	3	7	6	7	6	7	7	7
14	D4S2962	4q31	chr4:150364337	4	6	2	6	5	4	5	4	5	4
15	D4S413	4q32	chr4:158353155	1	9	1	9	9	9	9	9	9	15
16	D4S3046	4q32	chr4:163390792	3	4	2	4	2	5	2	5	5	5
17	D4S1597	4q32	chr4:169843115	2	8	2	8	1	5	1	5	2	5
18	D4S1539	4q34	chr4:175688140	1	3	1	3	3	1	3	1	2	1
19	D4S415	4q34	chr4:178711101	1	1	1	1	10	9	10	9	13	9

Suppl File 4

Locus 1

#	Gene	dbSNP 142	hg19	Ref	13		13		7		7	
					7303		7305		7176		7177	
1		D4S1572	chr4:103769921		4	7	4	1	7	1	7	1
2	CENPE	rs7686105	chr4:104061266		G	A			G	G	G	G
3	CENPE	rs1381657	chr4:104061993		C	G	C	G	C	C	C	C
4	CENPE	rs1381658	chr4:104062223		C	T	C	T	C	C	C	C
5	CENPE	rs2251322	chr4:104079424	C	C	T	C	T	C	C	C	C
6	CENPE	rs12504143	chr4:104101723	G	G	A	G	A	G	G	G	G
7	TACR3	rs2765	chr4:104510766	G	A		A		A	A	A	A
8	TACR3	rs3733632	chr4:104640935	A	A	G	A	G	A	G	A	A
9	CXXC4	rs3554	chr4:105390165	T	C	T	C	T	C	C	C	C
10	CXXC4	rs4475153	chr4:105392331	T	C	T	C	T	C	C	C	C
11	CXXC4	rs4282179	chr4:105393320	T	C	T	C	T	C	C	C	C
12	TET2	rs58201766	chr4:106155008	TAGATAGA	TAGATAGA	0	TAGATAGA	TAGATAGA	TAGATAGA	TAGATAGA	TAGATAGA	0
13	TET2	rs6843141	chr4:106155751	G	G	A	G	G	G	G	G	G
14	TET2	rs75056899	chr4:106158350	A	A	A	A	A	A	C	A	A
15	TET2	rs2454206	chr4:106196951	A	A	A	A	A	A	A	A	G
15	TET2	rs62621450	chr4:106197000	A	A	G	A	A	A	A	A	A
17	TET2	rs60786079	chr4:106197750	G	G	A	G	G	G	G	G	G
18	TET2		chr4:106197997	C	C	A	C	C	C	C	C	C
19	TET2	rs113581647	chr4:106198758	C	C	T	C	C	C	C	C	C

NPL 2.50

MCR1

TET2-TACR3 region

Locus 2

#	Gene	dbSNP 142	hg19	Ref	7303		7305		7176		7177	
20		D4S413	chr4:158353155		1	9	1	9	9	9	9	9
21	CDKN2AIP	rs11539203	chr4:184366145	C	C	C	C	C	C	T	T	T
22	STOX2		chr4:184826322	G	G	G	G	G	G	G	G	G
23	STOX2	rs36115739	chr4:184826381	A	T	A	T	A	T	A	T	T
24	STOX2	rs114230413	chr4:184827820	G	G	G	G	G	G	G	G	G
25	STOX2	SNP	chr4:184827881	G	G	G	G	G	G	G	G	G
26	STOX2	rs4600960	chr4:184839780	T	T	T	T	T	T	G	T	G
27	STOX2	rs5028430	chr4:184939798	T	T	T	T	T	T	G	T	G
28	STOX2	rs2309942	chr4:184839892	G	G	G	G	G	G	A	G	A
29	STOX2	rs28667509	chr4:184839844	G	G	A	G	A	G	G	G	G
30	STOX2	rs2309948	chr4:184881320	G	C	C	C	G	C	C	C	G
31	STOX2	rs116421761	chr4:184882307	G	G	G	G	G	G	C	G	C
32	STOX2	rs35046209	chr4:184909248	CC	CC	0	CC	0	CC	0	CC	0
33	STOX2	rs11731795	chr4:184910269	C	C	A	C	A	C	A	C	A
34	STOX2	rs3749534	chr4:184922669	G	G	A	G	A	G	A	G	A
35	STOX2	rs4861597	chr4:184931818	G	G	G	G	G	G	G	G	A
36	STOX2	rs4862282	chr4:184932609	G	A	A	A	A	A	A	A	A
37	STOX2	rs66511946	chr4:184932935	A	A	G	A	G	G	G	A	A
38	STOX2	rs6810667	chr4:184935603	C	C	C	C	C	C	C	C	T
39	STOX2	rs6836767	chr4:184935835	T	T	T	T	T	T	T	A	T
40	STOX2	rs11733294	chr4:184936136	C	T	T	T	T	T	T	C	T
41	STOX2	rs3811778	chr4:184936547	T	C	C	C	C	C	C	T	T
42	STOX2	rs3811777	chr4:184936604	A	A	A	A	A	A	A	G	G
43	STOX2	rs3811776	chr4:184936615	T	C	C	C	C	C	C	T	T
44	STOX2	rs3841107	chr4:184936677	TTTT	0	0	0	0	0	0	TTTT	TTTT
45	STOX2	rs3811775	chr4:184936790	G	A	A	A	A	A	A		
46	STOX2	rs2309956	chr4:184937312	G	A	A	A	A	A	A	G	A
47	STOX2	rs2871386	chr4:184937365	T	C	C	C	C	C	C	T	C
48	STOX2	rs10026246	chr4:184937370	C	C	C	C	C	C	C	C	T
49	STOX2	rs62339733	chr4:184937736	C	C	A	C	A	C	C	C	C
50	STOX2	rs6850441	chr4:184939570	G	C	C	C	C	C	C	C	C
51	STOX2	rs377270179	chr4:184939619	0	CCC	CCC	CCC	CCC	CCC	CCC	CCC	0
52	STOX2	rs6857152	chr4:184940696	A	G	G	G	G	G	G	G	G
53	STOX2	rs4862283	chr4:184940705	G	A	A	A	A	A	A	A	A
54	STOX2	rs4862284	chr4:184940790	T	T	T	T	T	T	T	T	T
55	STOX2	rs35661879	chr4:184941200	TAT	TAT	TAT	TAT	TAT	TAT	TAT	TAT	TAT
56	STOX2	rs375485832	chr4:184941206	T	T	T	T	T	T	T	T	T
57	STOX2	rs2309957	chr4:184942348	C	C	T	C	T	T	T	T	T
58	STOX2	rs2871387	chr4:184942404	G	C	G	C	G	C	C	C	C
59	STOX2	rs2309958	chr4:184942634	T	C	C	C	C	C	C	C	C
60	STOX2	rs1970895	chr4:184943106	G	G	G	G	G	G	G	G	G
61	STOX2	rs1970896	chr4:184943118	C	T	C	T	C	T	T	T	T
62	STOX2	rs3749531	chr4:184943274	T	G	T	G	T	G	G	G	G
63	STOX2	rs1133359	chr4:184943500	A	G	A	G	A	G	G	G	G
64	STOX2	rs56285832	chr4:184943975	C	C	T	C	T	C	C	C	C

NPL 3.13

MCR 2

STOX2-DDX60 region

Suppl File 5

Family 7

#	Gene	dbSNP 142	hg19	Ref	7		7		7		7		7		7		7	
					7173		7179		7171		7174		7177		7176		7180	
1	STOX2	rs62339673	chr4:184828533	C	A	C	A	C	C	C	C	C	A	C	A	C	A	C
2	STOX2	rs6836767	chr4:184935835	T	T	T	T	T	A	T	A	T	A	T	T	T	A	T
3	STOX2	rs11733294	chr4:184936136	C	T	T	T	T	C	T	C	T	C	T	T	T	C	T
4	STOX2	rs3811778	chr4:184936547	T	C	C	C	C	T	C	T	C	T	C	C	C	T	C
5	STOX2	rs3811776	chr4:184936615	T	C	C	C	C	T	C	T	C	T	C	C	C	T	C
6	STOX2	rs3841107	chr4:184936677	TTTT	0	0	0	0	TTTT	0	TTTT	0	TTTT	0	0	0	TTTT	0
7	STOX2	rs3811775	chr4:184936790	G	A	A	A	A	G	A	G	A			A	A	G	A
8	STOX2	rs2309956	chr4:184937312	G	A	A	A	A	G	A	G	A	G	A	A	A	G	A
9	STOX2	rs2871386	chr4:184937365	T	C	C	C	C	T	C	T	C	T	C	C	C	T	C

Family 13

#	Gene	dbSNP 142	hg19	Ref	13		13		13		13		13		13		13		13		13	
					7290		7292		7298		7308		7294		7302		7296		7303		7305	
1		rs4078181	chr4:184744873	G	A	T	A	T	A	T	A	T	A	T	A	T	A	T	T	T	A	T
2		rs34608496	chr4:184797917	C	C	T	C	T	C	T	C	T	C	T	C	T	C	T	T	T	C	T
3		rs13134353	chr4:184798026	T	T	G	T	G	T	G	T	G	T	G	T	G	T	G	G	G	T	G
4		rs7686859	chr4:184798422	C	A	C	A	C	A	C	A	C	A	C	A	C	A	C	C	C	A	C
5	STOX2	rs6552728	chr4:184917157	C	C	T	T	T	C	T	T	T	T	T	T	T	T	T	C	T	C	T
6	STOX2	rs6836767	chr4:184935835	T	A	T	T	T	A	T	T	T	T	T	T	T	T	T	T	T	T	T
7	STOX2	rs11733294	chr4:184936136	C	C	T	T	T	C	T	T	T	T	T	T	T	T	T	T	T	T	T
8	STOX2	rs3811778	chr4:184936547	T	T	C	C	C	T	C	C	C	C	C	C	C	C	C	C	C	C	C
9	STOX2	rs3811776	chr4:184936615	T	T	C	C	C	T	C	C	C	C	C	C	C	C	C	C	C	C	C
10	STOX2	rs3841107	chr4:184936677	TTTT	TTTT	0	0	0	TTTT	0	0	0	0	0	0	0	0	0	0	0	0	0
11	STOX2	rs3811775	chr4:184936790	G	G	A	A	A	G	A	A	A	A	A	A	A	A	A	A	A	A	A
12	STOX2	rs2309956	chr4:184937312	G	G	A	A	A	G	A	A	A	A	A	A	A	A	A	A	A	A	A
13	STOX2	rs2871386	chr4:184937365	T	T	C	C	C	T	C	C	C	C	C	C	C	C	C	C	C	C	C
14	STOX2	rs62339733	chr4:184937736	C	C	A	A	A	C	A	C	A	A	A	C	A	A	A	C	A	C	A

Suppl File 6

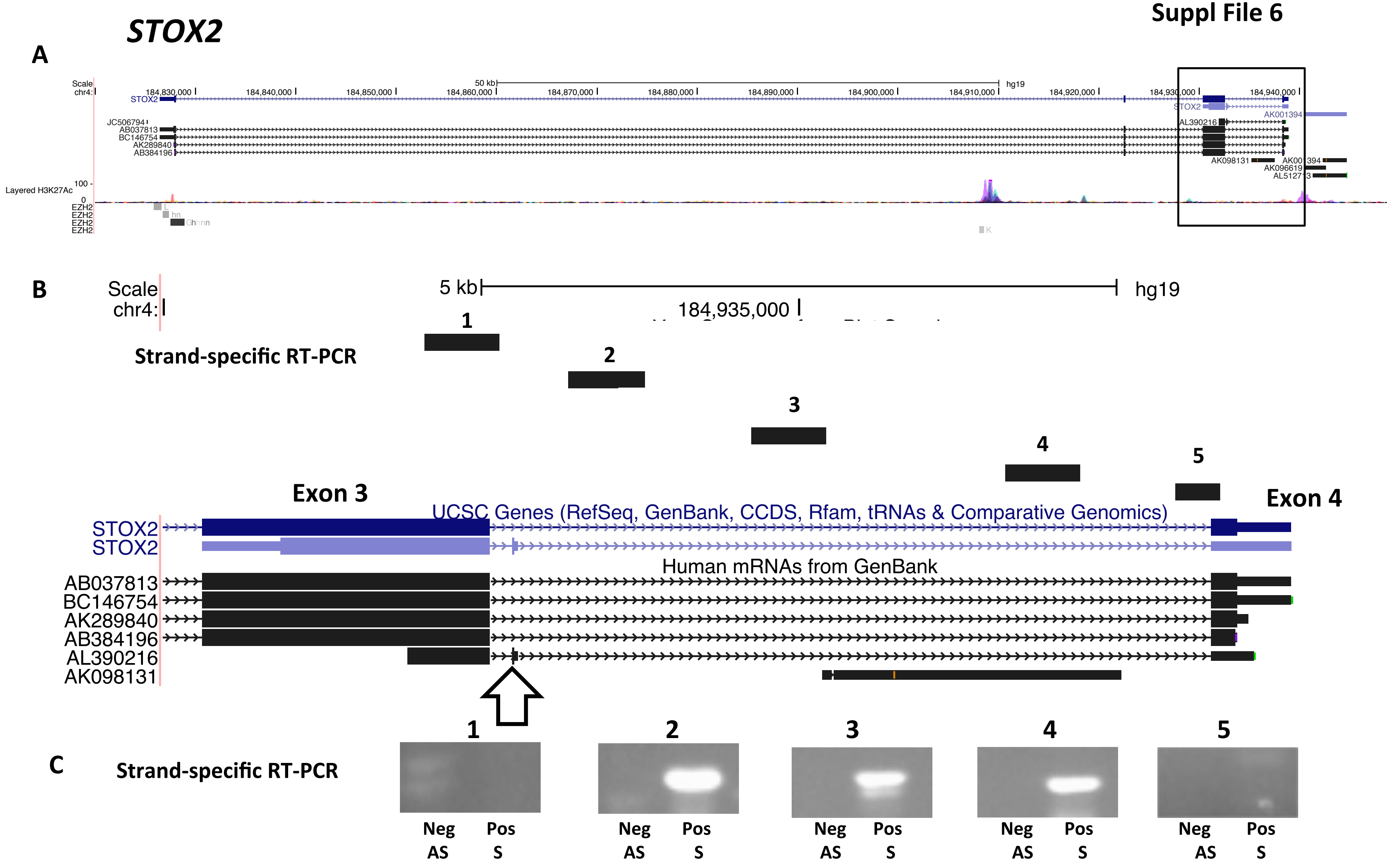
STOX2
A
Scale
chr4:
50 kb
hg19
184,830,000
184,840,000
184,850,000
184,860,000
184,870,000
184,880,000
184,890,000
184,900,000
184,910,000
184,920,000
184,930,000
184,940,000
STOX2
JC506794
AB037813
BC146754
AK289840
AB384196
AL390216
AK001394
AK098131
AK096619
Layered H3K27Ac
100 -
0
EZH2
B
Scale
chr4:
5 kb
hg19
184,935,000
1
2
3
4
5
Strand-specific RT-PCR
Exon 3
Exon 4
UCSC Genes (RefSeq, GenBank, CCDS, Rfam, tRNAs & Comparative Genomics)
STOX2
STOX2
Human mRNAs from GenBank
AB037813
BC146754
AK289840
AB384196
AL390216
AK098131
C
Strand-specific RT-PCR
1
2
3
4
5
Neg
Pos
AS
S

Suppl File 7

STOX2

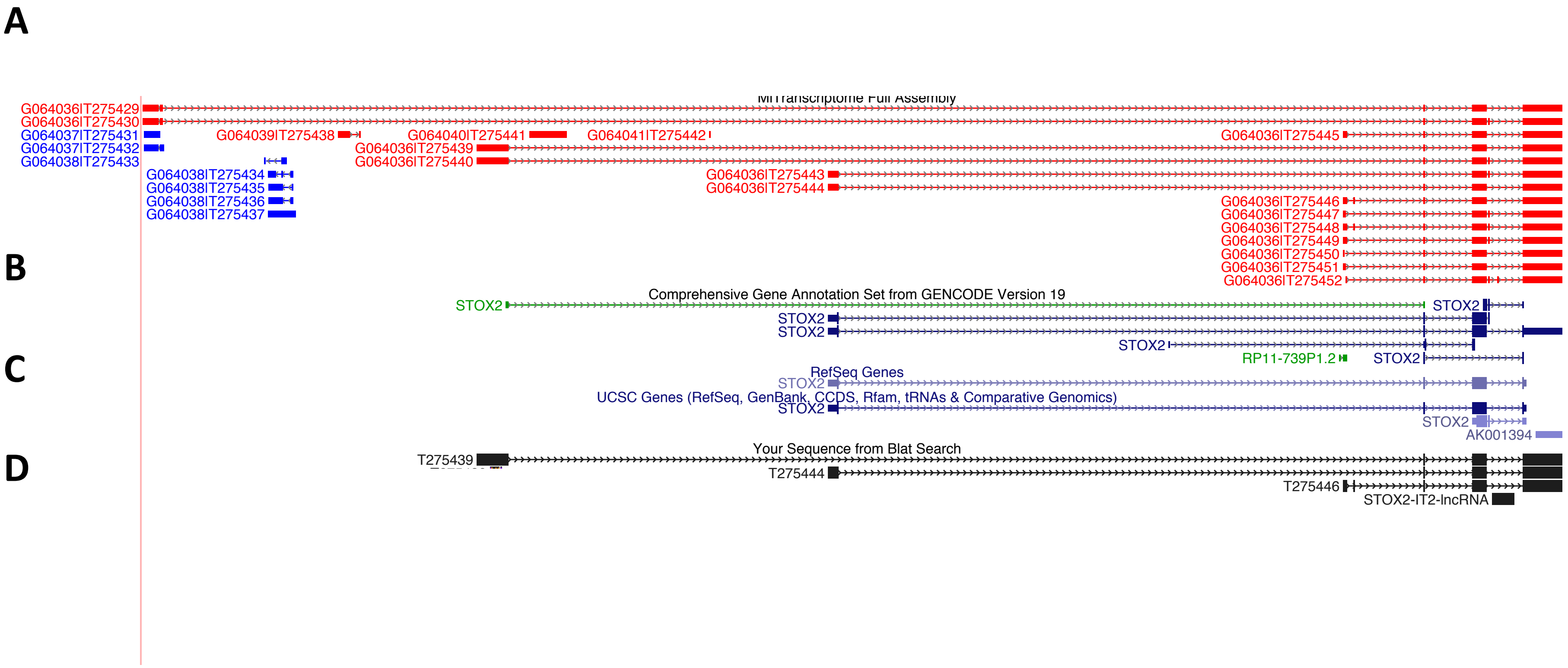

Supplementary File 8

```
T275446        ----------------------------------------------MFGQKKHKHGDVSP
T275439        ------------------------------------------------------------
T275444        MKKTRSTTLRRAWPSSDFSDRASDRMRSRSEKDYRLHKRFPAAFAPQASRGYMTSGDVSP


T275446        ISMSPISQSQFIPLGEILCLAISAMNSARKPVTQEALMEHLTTCFPGVPTPSQEILRHTL
T275439        --MSPISQSQFIPLGEILCLAISAMNSARKPVTQEALMEHLTTCFPGVPTPSQEILRHTL
T275444        ISMSPISQSQFIPLGEILCLAISAMNSARKPVTQEALMEHLTTCFPGVPTPSQEILRHTL
                 **********************************************************

T275446        NTLVRERKIYPTPDGYFIVTPQTYFITPSLIRTNSKWYHLDERIPDRSQCTSPQPGTITP
T275439        NTLVRERKIYPTPDGYFIVTPQTYFITPSLIRTNSKWYHLDERIPDRSQCTSPQPGTITP
T275444        NTLVRERKIYPTPDGYFIVTPQTYFITPSLIRTNSKWYHLDERIPDRSQCTSPQPGTITP
               ************************************************************

T275446        SASGCVRERTLPRNHCDSCHCCREDVHSTHAPTLQRKSAKDCKDPYCPPSLCQVPPTEKS
T275439        SASGCVRERTLPRNHCDSCHCCREDVHSTHAPTLQRKSAKDCKDPYCPPSLCQVPPTEKS
T275444        SASGCVRERTLPRNHCDSCHCCREDVHSTHAPTLQRKSAKDCKDPYCPPSLCQVPPTEKS
               ************************************************************

T275446        KSTVNFSYKTETLSKPKDSEKQSKKFGLKLFRLSFKKDKTKQLANFSAQFPPEEWPLRDE
T275439        KSTVNFSYKTETLSKPKDSEKQSKKFGLKLFRLSFKKDKTKQLANFSAQFPPEEWPLRDE
T275444        KSTVNFSYKTETLSKPKDSEKQSKKFGLKLFRLSFKKDKTKQLANFSAQFPPEEWPLRDE
               ************************************************************

T275446        DTPATIPREVEMEIIRRINPDLTVENVMRHTALMKKLEEEKAQRSKAGSSAHHSGRSKKS
T275439        DTPATIPREVEMEIIRRINPDLTVENVMRHTALMKKLEEEKAQRSKAGSSAHHSGRSKKS
T275444        DTPATIPREVEMEIIRRINPDLTVENVMRHTALMKKLEEEKAQRSKAGSSAHHSGRSKKS
               ************************************************************

T275446        RTHRKSHGKSRSHSKTRVSKGDPSDGSHLDIPAEREYDFCDPLTRVPREGCFIIEHKGDN
T275439        RTHRKSHGKSRSHSKTRVSKGDPSDGSHLDIPAEREYDFCDPLTRVPREGCFIIEHKGDN
T275444        RTHRKSHGKSRSHSKTRVSKGDPSDGSHLDIPAEREYDFCDPLTRVPREGCFIIEHKGDN
               ************************************************************

T275446        FIMHSNTNVLESHFPMTPEWDVSGELAKRRTEMPFPEPSRGSSHSKVHRSHSHTQDRRSR
T275439        FIMHSNTNVLESHFPMTPEWDVSGELAKRRTEMPFPEPSRGSSHSKVHRSHSHTQDRRSR
T275444        FIMHSNTNVLESHFPMTPEWDVSGELAKRRTEMPFPEPSRGSSHSKVHRSHSHTQDRRSR
               ************************************************************

T275446        NERSNKAKERSRSMDNSKGPLGASSLGTPEDLAEGCSQDDQTPSQSYIDDSTLRPAQTVS
T275439        NERSNKAKERSRSMDNSKGPLGASSLGTPEDLAEGCSQDDQTPSQSYIDDSTLRPAQTVS
T275444        NERSNKAKERSRSMDNSKGPLGASSLGTPEDLAEGCSQDDQTPSQSYIDDSTLRPAQTVS
               ************************************************************

T275446        LQRAHISSTSYKEVCIPEIVSGSKEPSSACSLLEPGKPPESLPSYGELNSCPTKTATDDY
T275439        LQRAHISSTSYKEVCIPEIVSGSKEPSSACSLLEPGKPPESLPSYGELNSCPTKTATDDY
T275444        LQRAHISSTSYKEVCIPEIVSGSKEPSSACSLLEPGKPPESLPSYGELNSCPTKTATDDY
               ************************************************************

T275446        FQCNTSSETVLTAPSPLGKNKEDHDTLTLAEGVKKLSPSDRQVPHSSREPVGHKEESPKG
T275439        FQCNTSSETVLTAPSPLGKNKEDHDTLTLAEGVKKLSPSDRQVPHSSREPVGHKEESPKG
T275444        FQCNTSSETVLTAPSPLGKNKEDHDTLTLAEGVKKLSPSDRQVPHSSREPVGHKEESPKG
               ************************************************************

T275446        PGGGPAASGGVAEGIANGRLVQHHGAEPSSLDKRKEIFSKDTLFKPLHSTLSVNSYHKSS
T275439        PGGGPAASGGVAEGIANGRLVQHHGAEPSSLDKRKEIFSKDTLFKPLHSTLSVNSYHKSS
T275444        PGGGPAASGGVAEGIANGRLVQHHGAEPSSLDKRKEIFSKDTLFKPLHSTLSVNSYHKSS
               ************************************************************

T275446        LSLLKSHPKTPADTLPGRCEKLEPSLGTSAAQAMPASQRQQESGGNQEASFDYYNVSDDD
T275439        LSLLKSHPKTPADTLPGRCEKLEPSLGTSAAQAMPASQRQQESGGNQEASFDYYNVSDDD
T275444        LSLLKSHPKTPADTLPGRCEKLEPSLGTSAAQAMPASQRQQESGGNQEASFDYYNVSDDD
               ************************************************************

T275446        DSEEGANKNTEEEKNREDVGTMQWLLEREKERDLQRKFEKNLTLLAPKETDSSSNQRATH
T275439        DSEEGANKNTEEEKNREDVGTMQWLLEREKERDLQRKFEKNLTLLAPKETDSSSNQRATH
T275444        DSEEGANKNTEEEKNREDVGTMQWLLEREKERDLQRKFEKNLTLLAPKETDSSSNQRATH
               ************************************************************

T275446        SARLDSMDSSSITVDSGFNSPRTRESLASNTSSIVESNRRQNPALSPAHGGAGPAFNFRA
T275439        SARLDSMDSSSITVDSGFNSPRTRESLASNTSSIVESNRRQNPALSPAHGGAGPAFNFRA
T275444        SARLDSMDSSSITVDSGFNSPRTRESLASNTSSIVESNRRQNPALSPAHGGAGPAFNFRA
               ************************************************************

T275446        SAEPPTNEAEKLQKPSNCLQASVTSV
T275439        SAEPPTNEAEKLQKPSNCLQASVTSV
T275444        SAEPPTNEAEKLQKPSNCLQASVTSV
               **************************
```

Suppl File 9

WESTERN BLOT STOX2

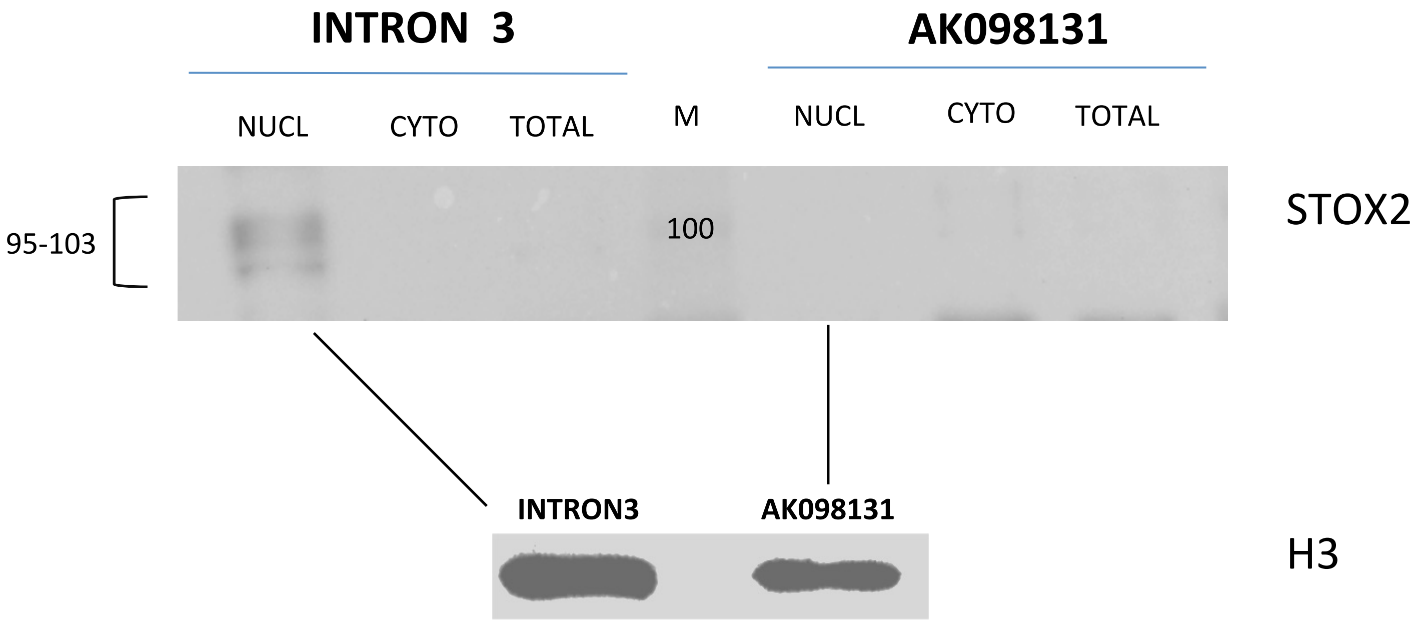

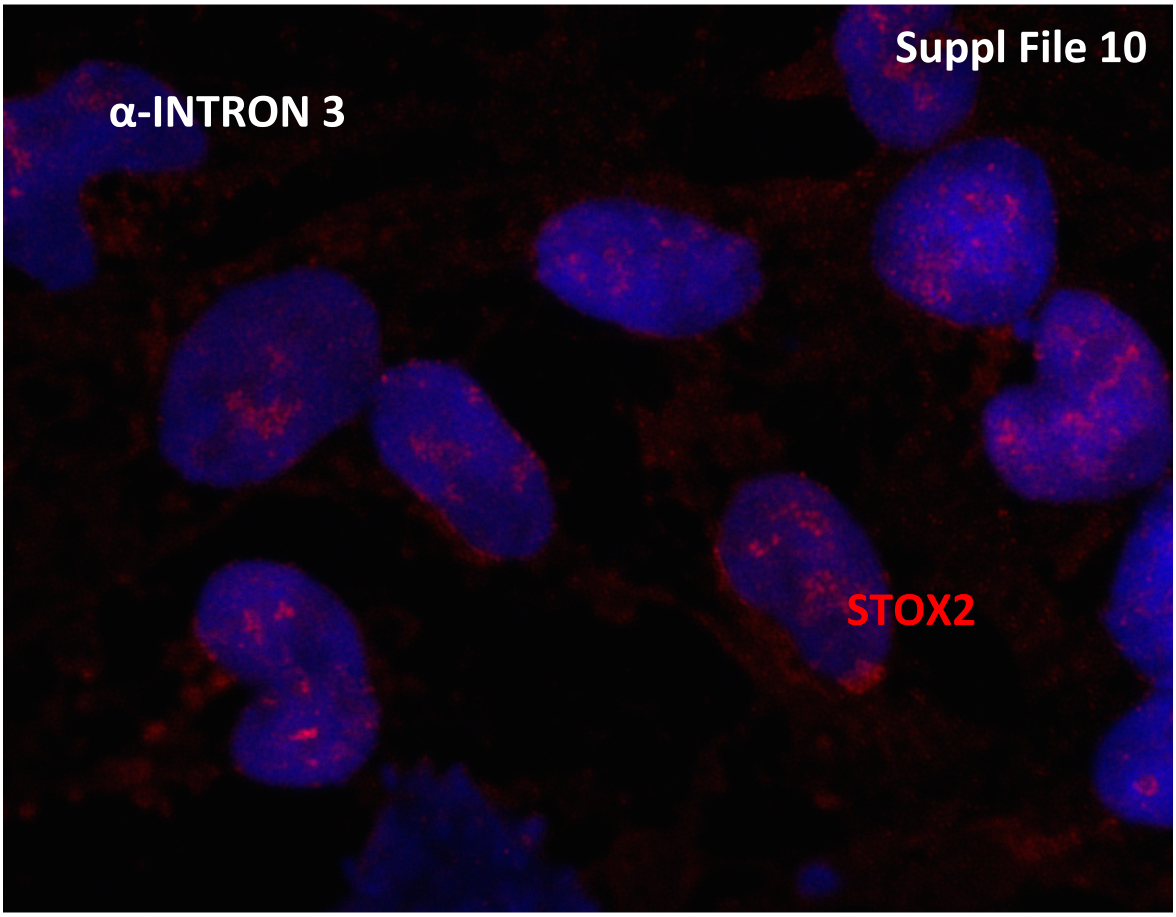
Suppl File 10
α-INTRON 3
STOX2

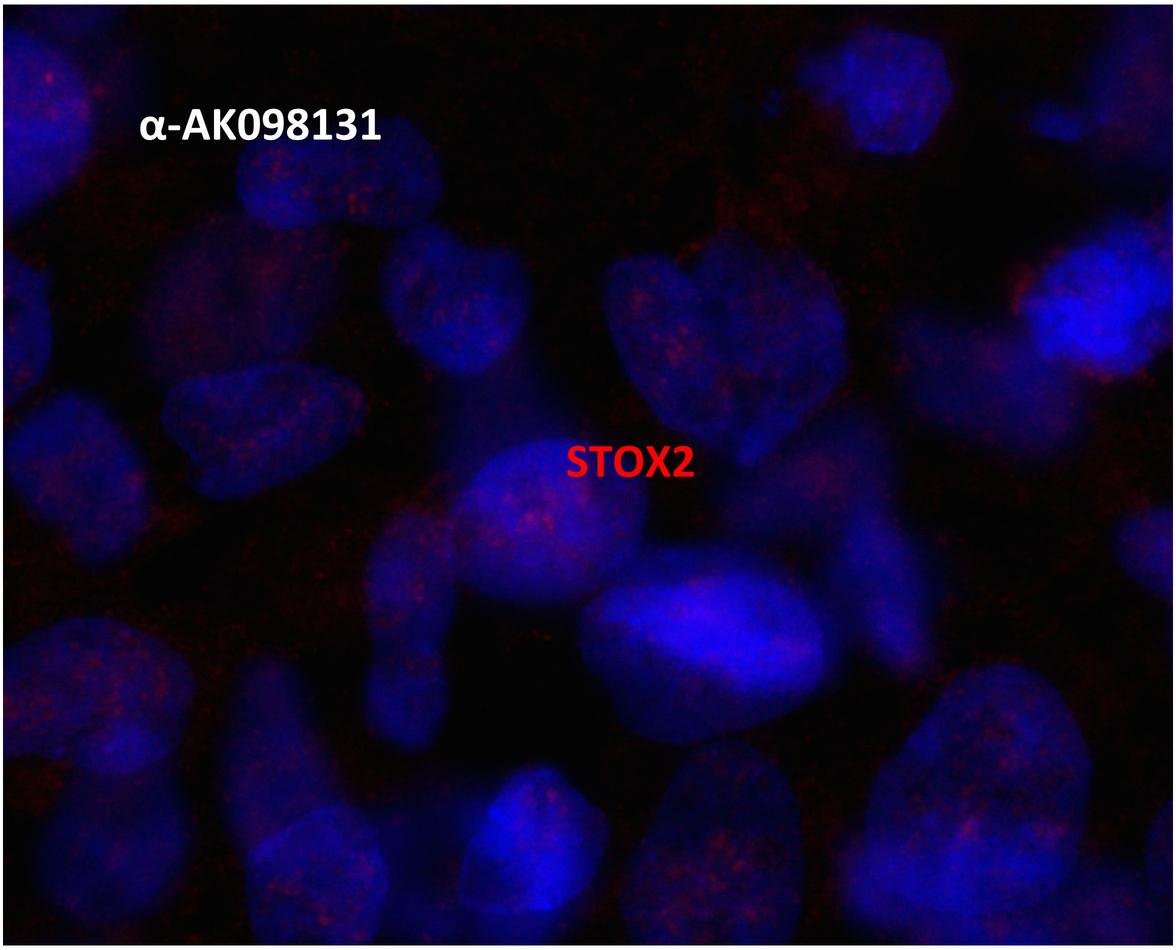
α-AK098131
STOX2

Suppl File 11

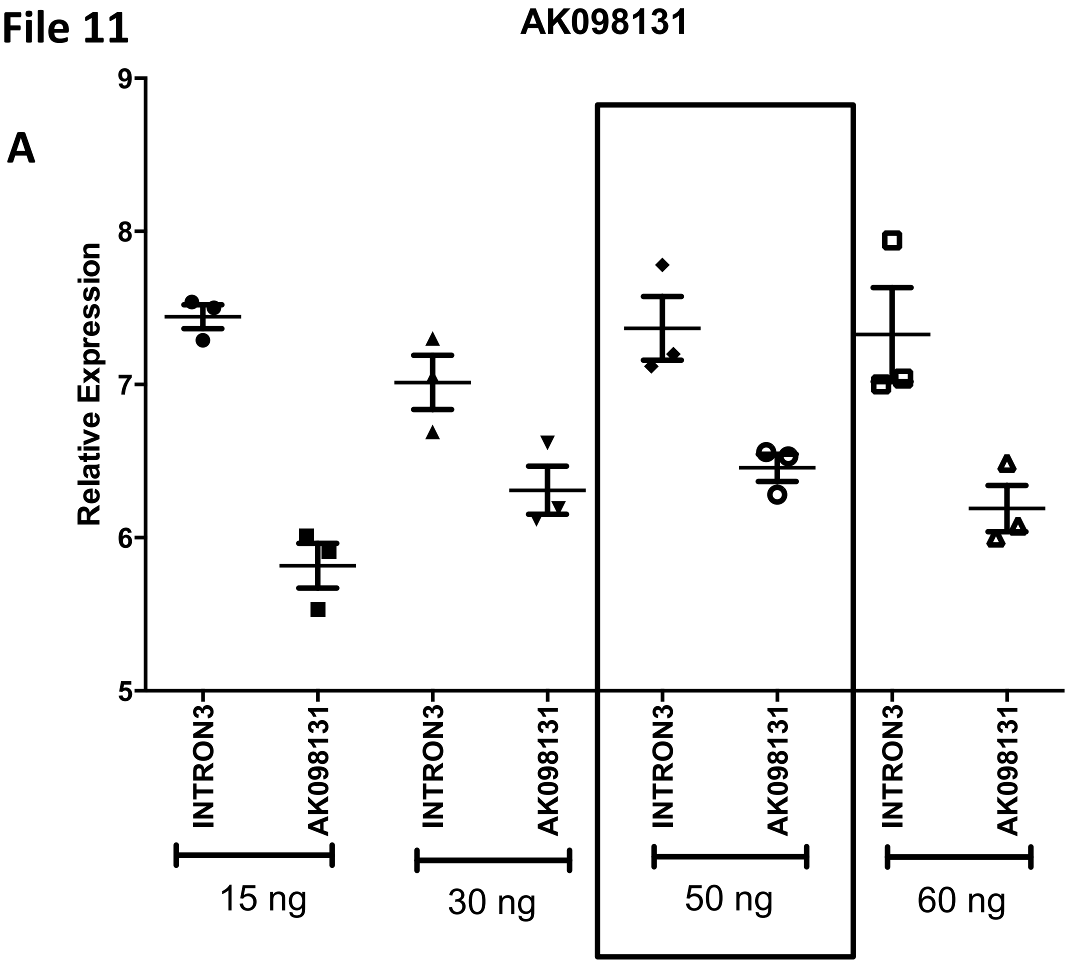

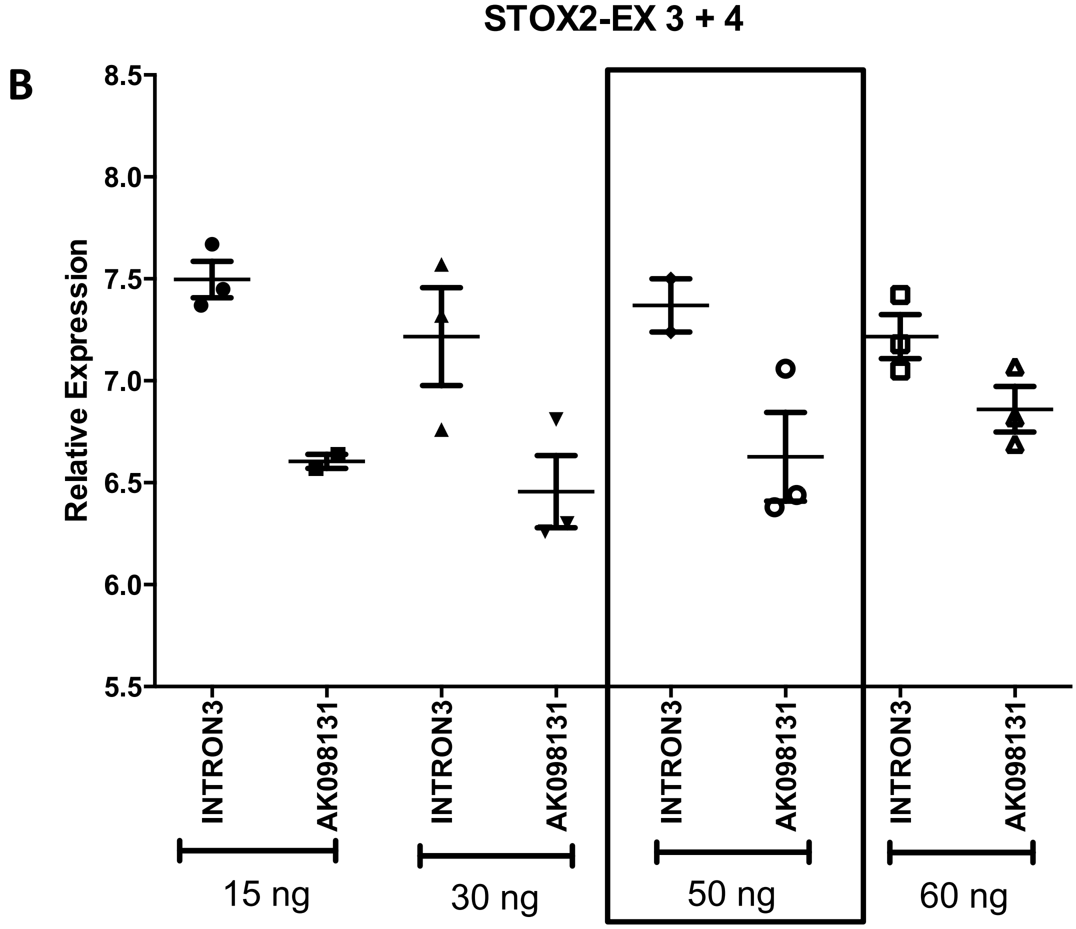

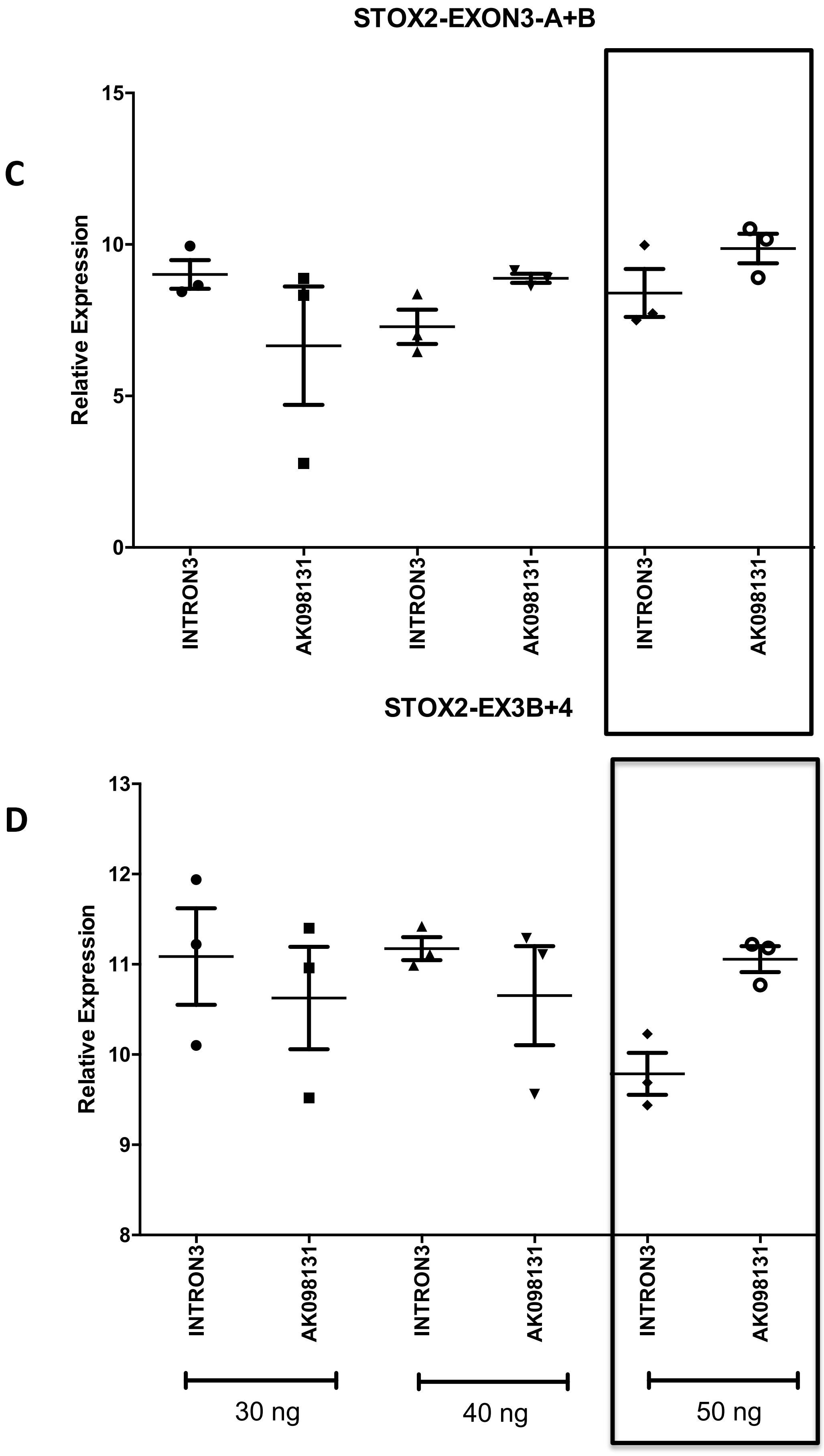
STOX2-EXON3-A+B
C
Relative Expression
15
10
5
0
INTRON3
AK098131
INTRON3
AK098131
INTRON3
AK098131
STOX2-EX3B+4
D
Relative Expression
13
12
11
10
9
8
INTRON3
AK098131
INTRON3
AK098131
INTRON3
AK098131
30 ng
40 ng
50 ng

Supple File 12

Hs1391761_m1

INTRON3

A

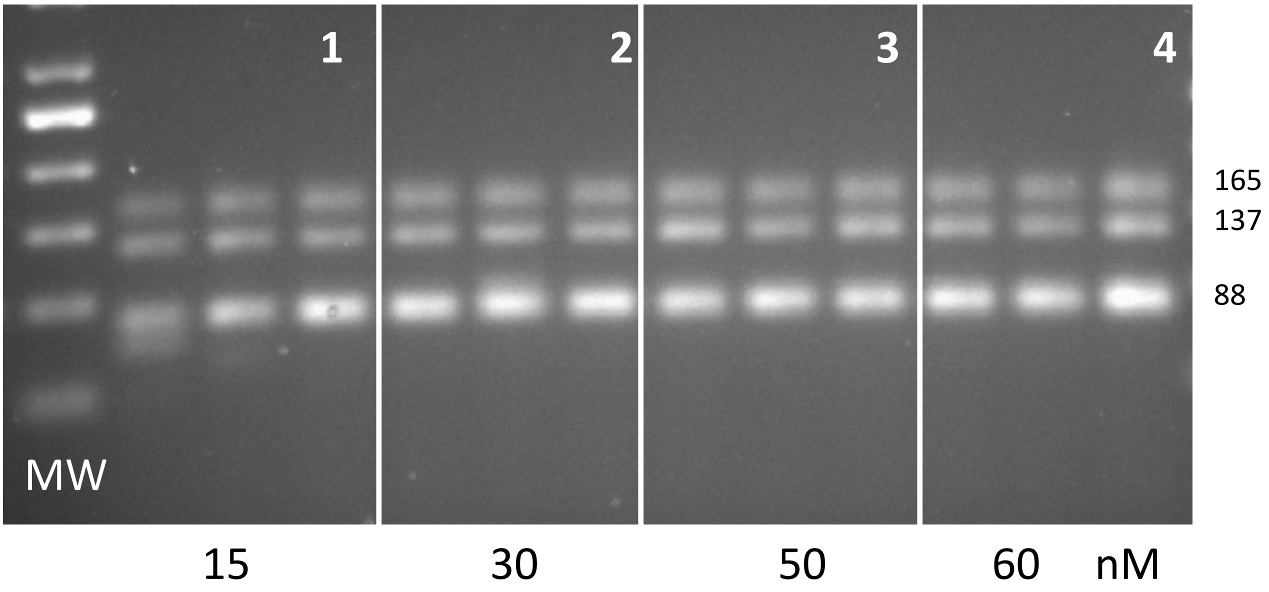

B

AK098131

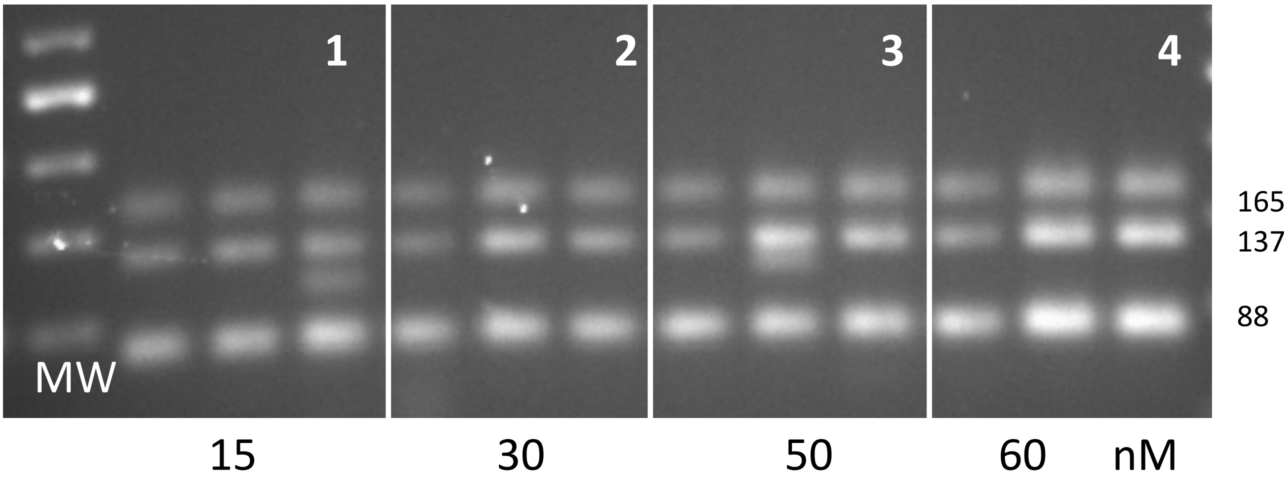

Suppl File 13

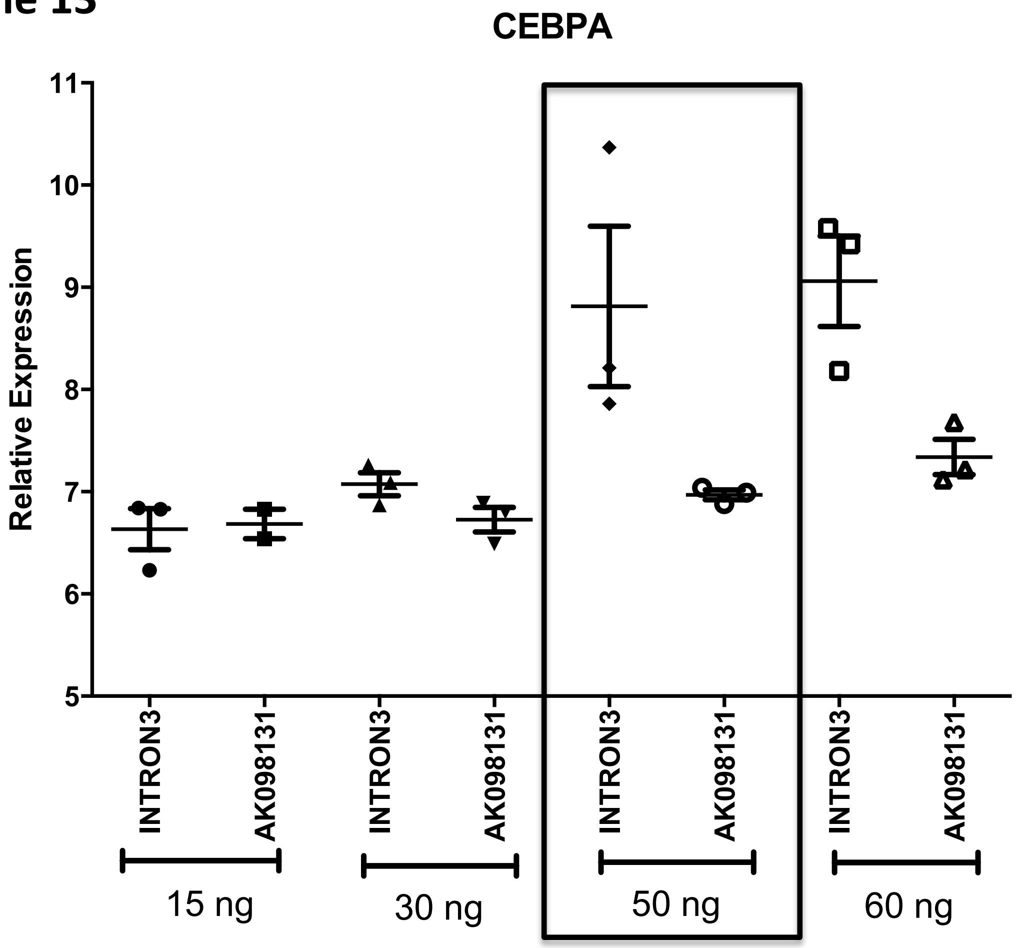

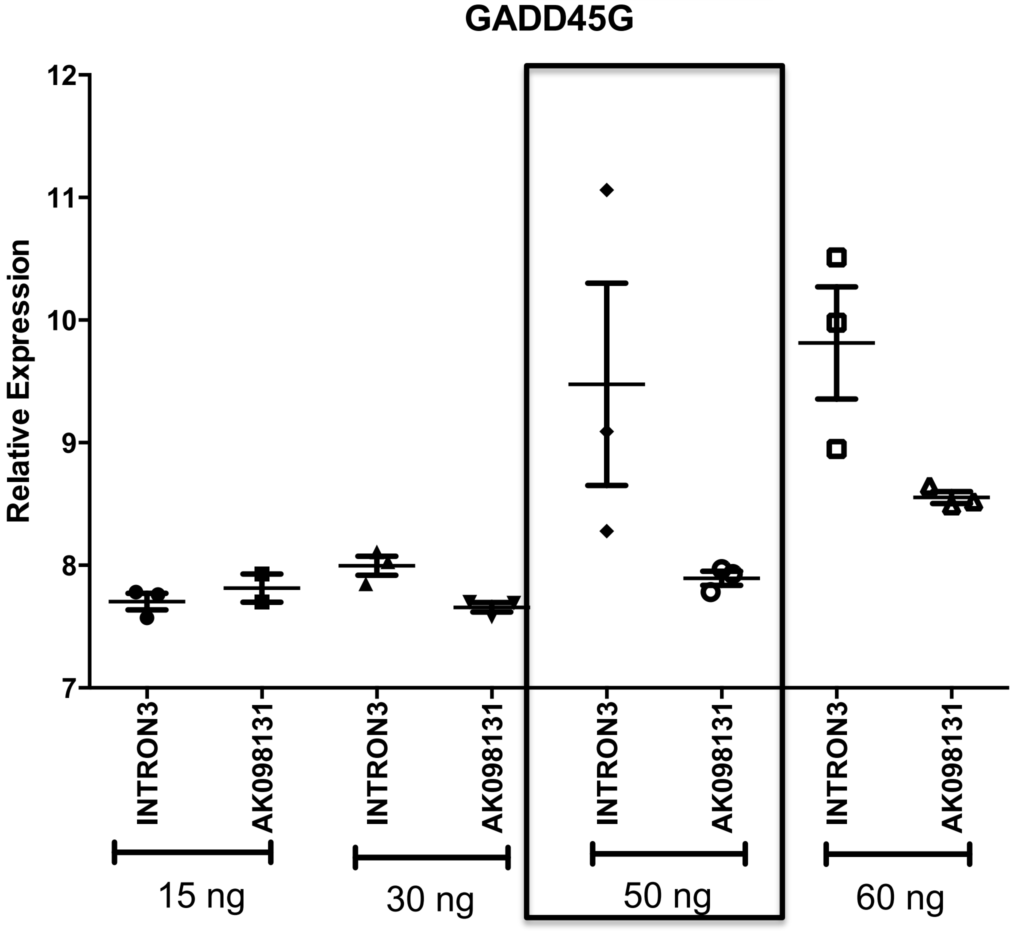

Suppl File 14

Chr4:184,882,511-184,932,935

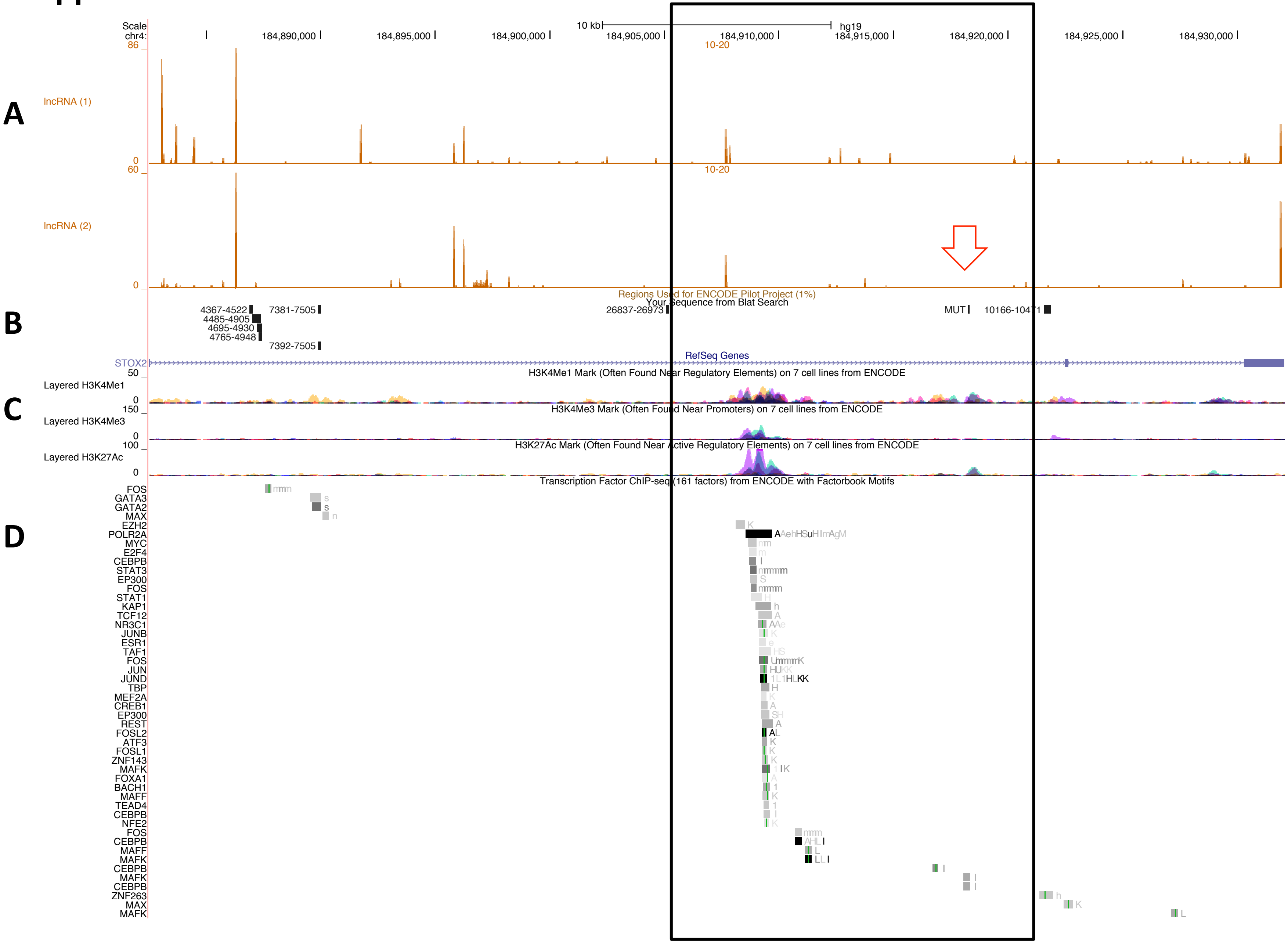

Supplementary File 15

STOX2 CRISPR PROTOCOL: cloning into GeneArt CRISPR nuclease OFP reporter vector

1. CRISPR sites in the STOX2 intragenic region with the second paternal mutation (G>T) located at chr4:184,918,288 (hg19)

gR#7:AACGCACATGCACACATGCA
gR#6: AAACGCACATGCACACATGC
gR#3:TAAACTCGGCTGTGATGGAA
gR#2:GACAGTAAACTCGGCTGTGA
gR#4:GGGAGAGAAGACAGTAAACT
AGTGGGTGAGACAGACAGTAAACCAGTG**A**ACCAAACGCACATGCACACAT**G**CAGGGAGAGAA**GACAGTAAACTCGGCTGTGA**TGGAATGGCAGTAGACCA
TCACCCACTCTGTCTGTCATTTGGT**CACTTGGTTTGCGTGTACGT**GTGTACGTCCCTCTCTTCTGTCATT**T**GAGCCGACACTACCTTACCGTCATCTGGT
gR#1:CACTTGGTTTGCGTGTACGT
gR#5:TTGCGTGTACGTGTGTACGT

Guide #	Score	Sequence	PAM
**Guide #1**	**82**	**TGCATGTGCGTTTGGTTCAC**	**TGG**
**Guide #2**	**81**	**GACAGTAAACTCGGCTGTGA**	**TGG**
Guide #3	74	TAAACTCGGCTGTGATGGAA	TGG
Guide #4	46	GGGAGAGAAGACAGTAAACT	CGG
Guide #5	42	TGCATGTGTGCATGTGCGTT	TGG
Guide #6	41	AAACGCACATGCACACATGC	AGG
**Guide #7**	**28**	**AACGCACATGCACACATGCA**	**GGG**

2. Complementary CRISPR target site oligonucleotides with overhang used for cloning

STOX2

STOX2-gR#1a 5'-**CATGTGCGTTTGGTTCAC**GTTTT-3'
STOX2-gR#1b 5'-**GTGAACCAAACGCACATG**CGGTG-3'

STOX2-gR#2a 5'-**ACAGTAAACTCGGCTGTGA**GTTTT-3'
STOX2-gR#2b 5'-**TCACAGCCGAGTTTACTGT**CGGTG-3'

STOX2-gR#7c 5'-**CGCACATGCACACATGCA**GTTTT-3'
STOX2-gR#7d 5'-**TGCATGTGTGCATGTGCG**CGGTG-3'

Positive control

p53-gRa 5'-CATTGTTCAATATCGTCCGgtttt-3'
p53-gRb 5'-CGGACGATATTGAACAATGcggtg-3'

Negative control

NC-gRa 5'-CCTCTTACCTCAGTTACAATgtttt-3'
NC-gRb 5'-ATTGTAACTGAGGTAAGAGGcggtg-3'

3. Cloning in GeneArt CRISPR Nuclease OFP reporter vector

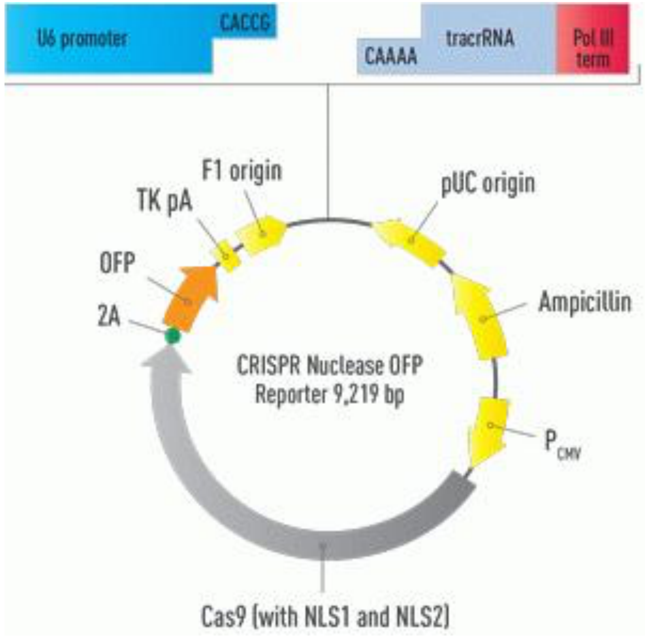

Oligonucleotide annealing, ligation and transformation of TOP10 E.coli cells was done according to the manufacturers instructions (GeneArt CRISPR Nuclease Vector kit) (Life Technologies).

4. Identification of insert-containing colonies by colony PCR

Screen 1-hour cultures of individual colonies by colony PCR using a common upstream primer (U6-forward 5’-ACT ATC ATA TGC TTA CCG TAA C-3’) in combination with the target site specific, second (lower) oligonucleotide. Select positive colonies (97 bp PCR fragment) (arrow in **picture**, marker is 50 bp ladder) and isolate plasmid DNA by affinity purification (QIAgen).

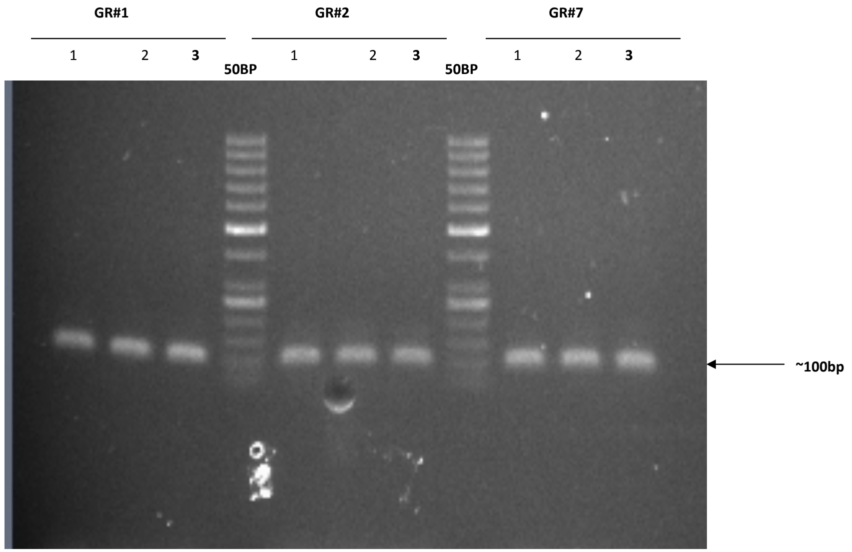

Colony PCR with U6 (**F**) and GR# (**R**) primers

5. Confirmation of inserts by DNA sequencing of plasmids using U6-forward primer

Target 1

STOX2-gR#1 5'-caccg**CATGTGCGTTTGGTTCAC**-3'

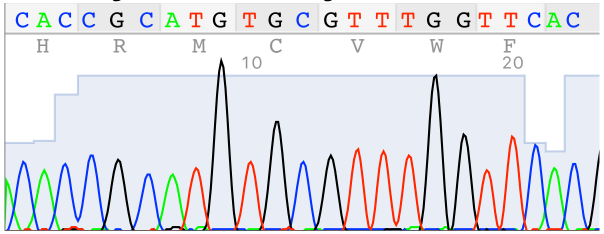

Target 2

STOX2-gR#2a 5'-caccg**ACAGTAAACTCGGCTGTGA**-3'

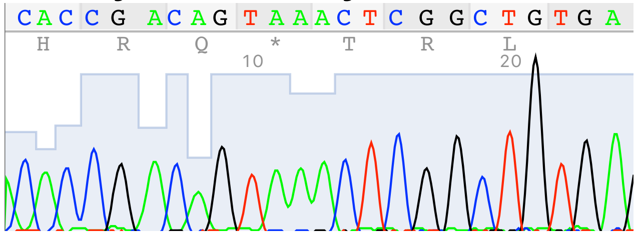

Target 3

STOX2-gR#7c 5'-caccg**CGCACATGCACACATGCA**-3'

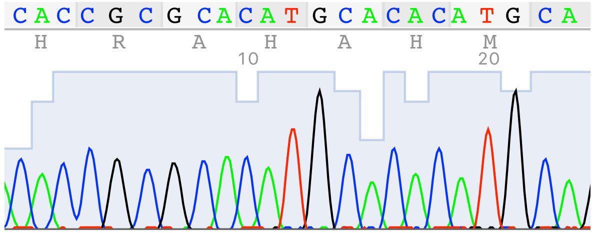

Positive control

p53-gRa 5’-CATTGTTCAATATCGTCCGgtttt-3’

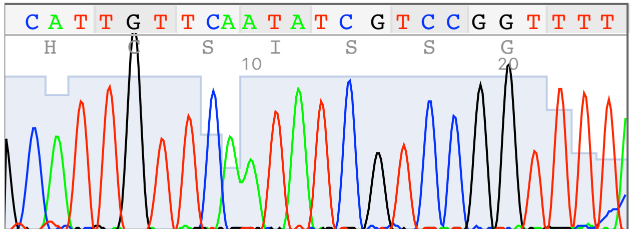

Negative control

NC-gRa 5’-CCTCTTACCTCAGTTACAATgtttt-3’

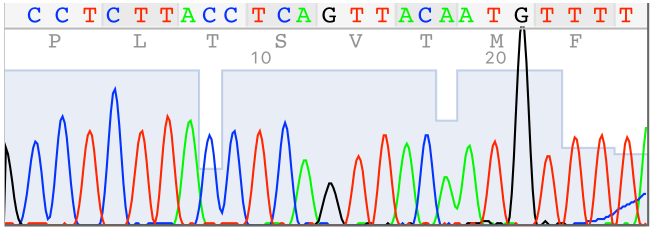

6. Transfection of SGHPL5 cells: optimalization and confirmation of intended effects

Plating

Day 1: Harvest SGHPL5 cells (70-90% confluency)
Count using Countess cell counter
Plate (48 wells): 30,000 cells/well in 250 μl complete medium (Iscove's with 10% FBS and penicillin/streptomycin)

Transfection

Day 2: Allow the Fugene HD reagent to reach room temperature.
Mix by inverting gently.

To a sterile tube, add the required amount of prewarmed medium (without FBS and without P/S). Add DNA (from 100 ng/μl stock) and vortex. Add Fugene HD directly and mix immediately (final volume 15 μl).

Incubate reagent/DNA mixture for 15 min at RT.

Add 235 μl of complete medium to each reagent/DNA mixture and mix thoroughly, but gently.

Replace medium in wells with 250 μl of the medium/Fugene/DNA mixture.

Fugene/DNA ratio used was 15 with 100-350 ng plasmid DNA per well. Amount of Fugene HD needed was calculated by the formula: ng plasmid/1000 x ratio = μl Fugene HD

Analysis

Day 4: Incubate cells for 48 hours. Harvest cells. Score efficiency by OFP analysis. Score genome editing by DNA sequencing.

Transfection efficiency with CRISPR construct #7

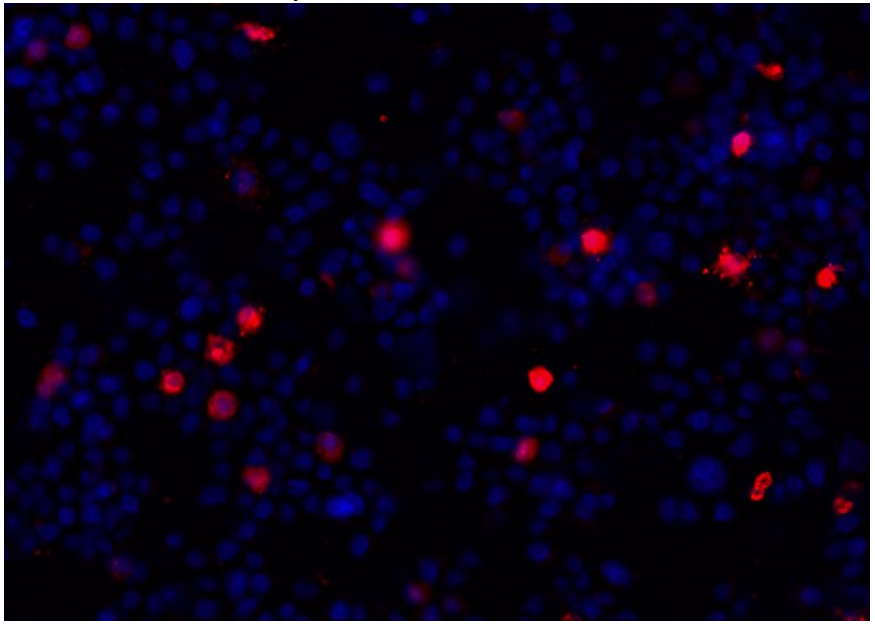

7. Readout: qRT-PCR of STOX2 transcripts

Suppl File 16

NORMAL

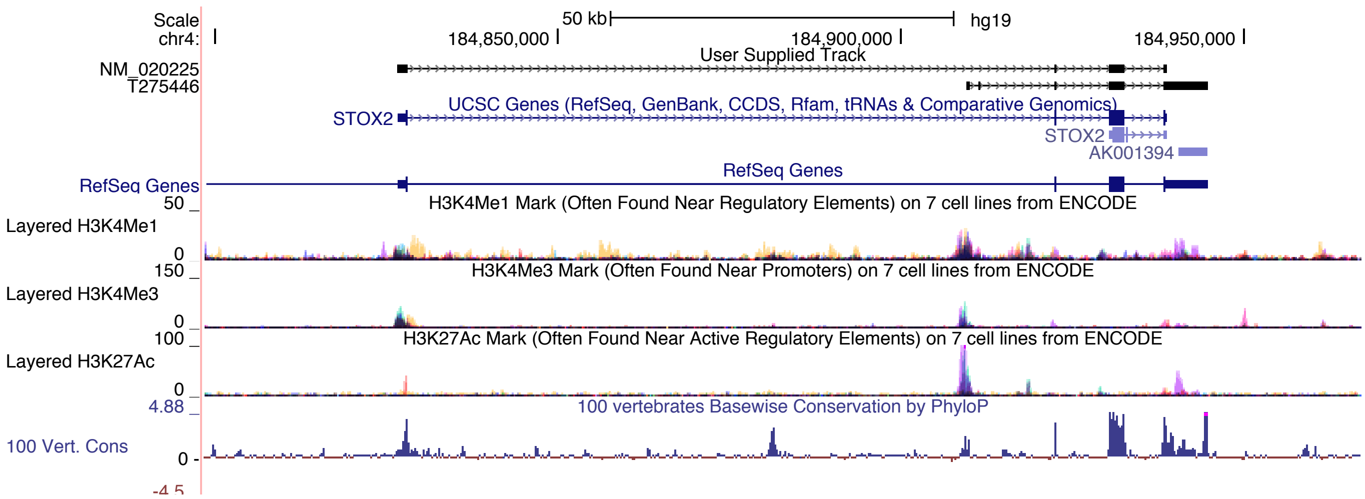

DISEASE

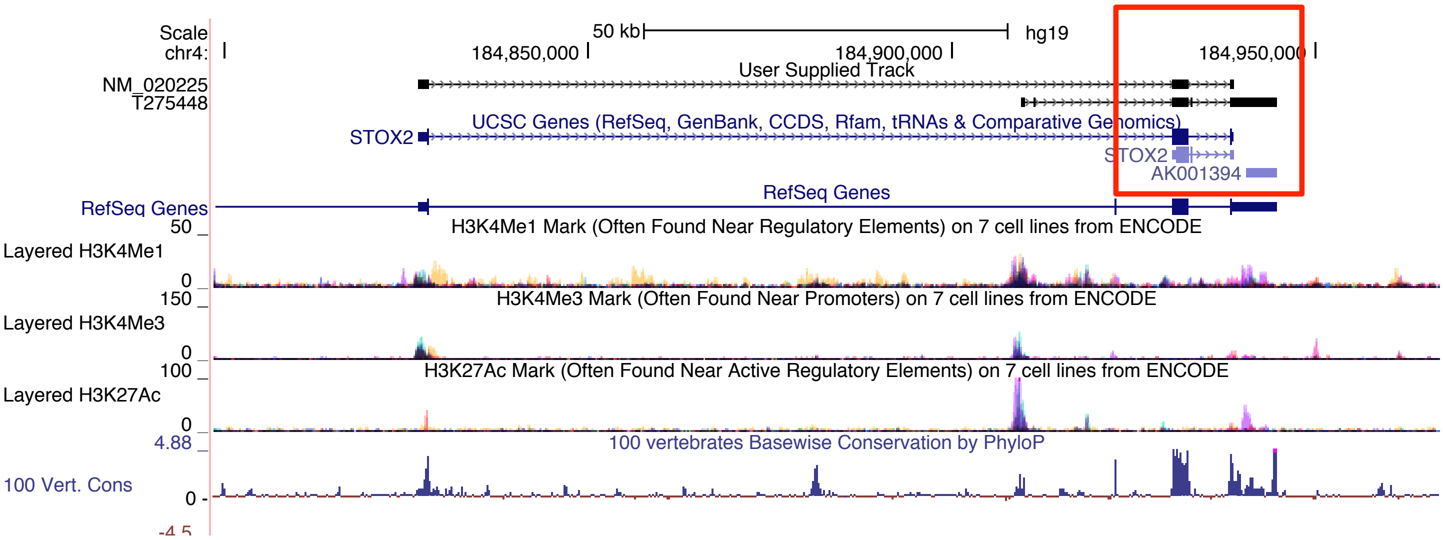

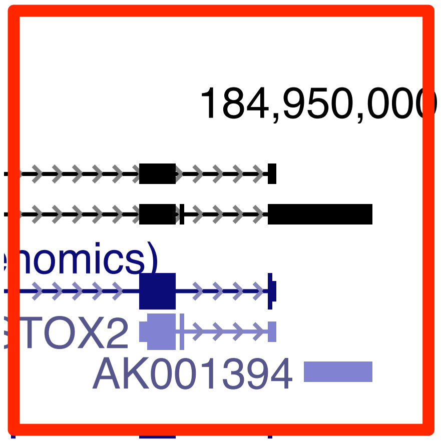

Suppl File 17A

Family 7

						7		7		7		7		7		7		7		7		7		7		7	
#	Gene	dbSNP 142	hg19	Ref	MAF	7173		7172		7179		7171		7170		7174		7177		7176		7175		7178		7180	
1			chr4:184705736	G		G	G			G	G	G	T	G	T	G	G					G	T	G	G	G	G
2			chr4:184705774	A		A	A			A	A	A	G	A	G	A	A					A	G	A	A	A	A
3			chr4:184705807	C		C	C			C	C	C	A	C	A	C	C					C	A	C	C	C	C
4		rs11356631	chr4:184705881	A	8.866	A	A			A	0	A	A	A	A	A	A					A	A			A	A
5		rs62342249	chr4:184716354	A	20.607	A	A	A	A	A	A	A	A	A	A	A	A	A	A	A	A	A	G	A	A	A	G
6		rs138260968	chr4:184716514	TATT	1.857	4	4	4	0	4	4	4	4	4	4	4	4	4	4	4	4	4	4	4	4	4	4
7		rs60604513	chr4:184716840	T	15.695	T	T	T	C	T	C	T	T	T	T	T	T	T	T	T	T	T	T	T	T	T	T
8		rs60839040	chr4:184717536	C	23.542	C	C	C	C	C	C	C	C	C	C			C	C	C	C	C	A	C	C	C	A
9		rs367688566	chr4:184717537	20		20	20	20	20	20	20	20	20	20	20	20	20	20	20	20	20	20	20	20	20	20	20
10		rs62342250	chr4:184718319	T	17.792			T	T	T	T					T	C	C	C	T	C	T	C			C	C
11		rs62342251	chr4:184718676	C	16.454	C	C	C	T	C	T	C	C	C	C			C	C	C	C	C	C	C	C	C	C
12		rs75942758	chr4:184719247	A	18.091	A	C	A	C	A	C	A	C	C	C	A	C	C	C	A	C	A	C	C	C	C	C
13		rs67103969	chr4:184719252	A	41.713	G	A	A	A	A	A	G	A	A	A	G	A	A	A	G	A	G	A	A	A	A	A
14		rs146992605	chr4:184719283	C	2.276	C	C	C	C	C	C	C	C	C	C	C	C	C	C	C	C	C	C	C	C	C	C
15		rs116049039	chr4:184719284	C	2.276	C	C	C	C	C	C	C	C	C	C	C	C	C	C	C	C	C	C	C	C	C	C
16		SNP	chr4:184719654	C		C	C	C	C	C	C	C	C	C	C			C	C	C	C	C	C	C	C	C	C
17		rs72693687	chr4:184719992	T	7.668	T	T	T	T	T	T	T	T	T	T	T	T	T	T	T	T	T	T	T	T	T	T
18		rs78839475	chr4:184719996	C	7.648	C	C	C	C	C	C	C	C	C	C	C	C	C	C	C	C	C	C	C	C	C	C
19		rs386683142	chr4:184720045	T		16	16	16	16	16	16	16	16	16	16	16	16	16	16	16	16	16	16	16	16	16	16
20		rs9990869	chr4:184720139	C	38.478	C	T	C	T	C	T	C	T	T	T	C	T	C	T	C	T	C	T	T	T	C	T
21		rs72693688	chr4:184720199	C	2.117	C	C	C	T	C	C	C	T	C	T	C	T	C	C	C	C	C	C	C	T	C	C
22		rs11722400	chr4:184727641	A	36.921	A	C	A	C	C	C	A	A	A	C			A	C	A	C	A	A	A	C	A	C
23		rs4078180	chr4:184744832	C	17.951	C	C			C	C	C	C	C	C	C	C			C	C			C	C	C	C
24		rs4078181	chr4:184744873	G	14.357	G	G			G	G	G	G	G	G	G	G			G	G			G	G	G	G
25			chr4:184749538	C		C	T	C	C	C	C	C	T	C	C	C	T	C	C	C	T	C	C	C	C	C	C
26			chr4:184749921	A		A	G	A	A	A	G	A	A	A	A	A	A	A	A	A	A	A	A	A	A	A	A
27		rs75766856	chr4:184749987	G	1.857																						
28		rs149453332	chr4:184753805	G	2.097	G	A	G	G	G	A	G	G	G	G	G	G	G	G	G	G	G	G	G	G	G	G
29		rs140928377	chr4:184770875	G	1.777	G	G	G	G	G	G	G	G	G	A	G	G			G	A	G	G	G	A	G	A
30		rs6813846	chr4:184770923	G	34.565	G	G	G	G	G	G	G	A	G	G	G	A			G	G	G	A	G	A	G	G
31		rs62340391	chr4:184773893	A	42.372	A	G	G	G	G	G	A	A			A	A					A	A	A	A	A	A
32		rs6840225	chr4:184774772	A	40.196	A	G	A	A	A	A	G	G	A	G	G	G	G	G	G	G	G	G	G	G	G	G
33		rs56348474	chr4:184776401	T	38.978	T	C	T	T	T	T	C	C	T	C	C	C	C	C	C	C	C	C	C	C	C	C
34		rs2033626	chr4:184776546	G		G	C	G	G	G	G	C	C	G	G	C	C	G	C	G	C	C	C	G	C	G	C
35		rs9997501	chr4:184776644	C	40.236	C	T	C	C	C	T	T	T	C	T	T	T	T	T	T	T	T	T	T	T	T	T
36		rs147162710	chr4:184776666	A	0.160	A	G	A	A	A	G	A	A	A	A	A	A	A	A	A	A	A	A	A	A	A	A
37		rs6552712	chr4:184779462	C	44.010	C	T			T	T	C	C	C	T	C	C	C	C	C	C	C	C	C	C	C	C
38		rs12499183	chr4:184780717	C	39.497	C	G	C	C	C	C	G	G	C	G	G	G	G	G	G	G	G	G	G	G	G	G
39		rs892838	chr4:184780272	T		T	G	T	T	T	T	G	G	T	G	G	G	G	G	G	G	G	G	G	G	G	G
40		rs6852989	chr4:184781947	G	8.846	T	T	T	T	T	T	T	T	T	T	T	T	T	T	T	T	T	T	T	T	T	T
41		rs11944432	chr4:184797519	T	34.105	A	A	A	T	A	T	A	A	A	T	A	A	A	T	A	T	A	T	A	T	A	T
42			chr4:184797521	G		G	G	G	G	G	G	G	G	G	G	G	G	G	G	G	G	G	G	G	G	G	G
43		rs78932922	chr4:184797708	G	0.679	G	G	G	G	G	G	G	G	G	G	G	G	G	G	G	G	G	G	G	G	G	G
44		rs34608496	chr4:184797917	C	28.894	C	C	C	T	C	T	C	C			C	C	C	T			C	T	C	T	C	T
45		rs13134353	chr4:184798026	T	28.994	T	T	T	G	T	G	T	T			T	T	T	G			T	G	T	G	T	G
46		rs7686859	chr4:184798422	C	49.341			C	A	A	C	A	A			A	A	A	C	A	C	A	C	A	C	A	C
47		rs6813664	chr4:184788700	A	34.156	G	G	G	A	G	A	G	G	G	A	G	G	G	A	G	A	G	A	G	A	G	A
48		rs4146058	chr4:184798737	G	33.127	A	A	A	G	A	G	A	A	A	G	A	A	A	G	A	G	A	G	A	G	A	G
49		rs2164301	chr4:184798865	G	29.213	G	G	G	A	G	A	G	G	G	A	G	G	G	A	G	A	G	A	G	A	G	A
50		rs7695698	chr4:184801157	T	22.005	T	T	T	G	T	G	T	T	T	G	T	T	T	G	T	G	T	G	T	G	T	G
51		SNP	chr4:184807252	G		G	TAG	G	G	G	TAG	G	G	G	G	G	G			G	G	G	G	G	G	G	G
52		rs372520827	chr4:184807256	G				G	C			G	G	G	C	G	G			G	C	G	C	G	C	G	C
53		rs376044001	chr4:184807257	A				A	T			A	A	A	T	A	A			A	T	A	T	A	T	A	T
54		rs71622933	chr4:184807283	A	50.000			A	C			A	A	A	C	A	A			A	C	A	C	A	C	A	C
55		rs12649564	chr4:184807335	C	50.000			A	C			A	A	A	C	A	A			A	C	A	C	A	C	A	C
56		rs12649564	chr4:184807396	C	42.857			C	T			C	C	C	T	C	C			C	T	C	T	C	T	C	T
57		SNP	chr4:184807491	C				A	C					A	C					A	C	A	C			A	C
58		SNP	chr4:184807500	A				T	C					T	C					T	C	T	C			T	C
59		rs398083846	chr4:184807508	G										G	0												
60		rs181100773	chr4:184807730	T	0.020	T	T	T	T	T	T	T	T	T	T	T	T			T	T	T	T	T	T	T	T
61		rs4990039	chr4:184811610	T	29.972	C	C	C	C	C	C	C	C			C	C	C	C	C	T	C	T	C	T	C	T
62		rs13130260	chr4:184824352	G	31.889	G	A	G	A	G	A	G	A	G	G	G	A	G	G	G	A	G	A	G	G	G	A
63		rs114894140	chr4:184824378	A	1.338	A	A	A	A	A	A	A	G	A	A	A	G	A	A	A	A	A	A	A	G	A	A
64		rs188804673	chr4:184824996	G	0.240	G	G	G	G	G	G	G	G	G	T	G	G	G	T	G	T	G	G	G	T	G	T
65		rs10017668	chr4:184825092	A	21.486	A	A	A	A	A	A	A	T	A	A	A	T	A	A	A	A	A	A			A	A
66		rs10005808	chr4:184825170	C	21.545	T	T	T	T	T	T	T	C	T	T	T	C	T	T	T	T	T	T			T	T
67	STOX2	rs17075136	chr4:184825696	A	29.513	A	G	A	G	A	G	A	G			A	G	A	A	A	G	A	G	A	A	A	G
68	STOX2	rs1560982	chr4:184826026	G	22.624	G	A			G	A	A	A			G	G	G	A	G	A			G	A	G	A
69	STOX2	rs36115739	chr4:184826381	A	48.403	A	T	A	T	A	T	A	A			A	A	A	T	A	T	A	T	A	T	A	T
70	STOX2	rs62339673	chr4:184828533	C	49.720	A	C	A	C	A	C	C	C			C	C	A	C	A	C	A	C	A	C	A	C
71	STOX2	rs7689460	chr4:184830322	T	35.643	T	G	G	G	T	G	G	G	T	G	G	G	T	G	T	G	T	G	T	G	T	G
72	STOX2	rs112673876	chr4:184830631	C	5.032	C	C	C	C	C	C	C	C	C	C	C	C	C	C	C	C	C	C	C	C	C	C
73	STOX2	rs12647459	chr4:184830682	T	35.982	T	G	G	G	T	G	G	G	T	G	G	G		G	T	G	T	G	T	G	T	G
74	STOX2	rs12644266	chr4:184830847	C	35.963	C	T	T	T	C	T	T	T	C	T	T	T	C	T	C	T	C	T	C	T	C	T
75	STOX2	rs113262003	chr4:184830913	0	35.783	0	4	4	4	0	4	4	4	0	4	4	4	0	4	0	4	0	4	0	4	0	4
76	STOX2	rs4862265	chr4:184831428	C	49.002	T	C	C	C	T	C	C	C	T	C	C	C	T	C	T	C			T	C	T	C
77	STOX2	rs4862266	chr4:184831454	C	42.732	T	C	C	C	T	C	T	C	T	C	T	C	T	T	T	C			T	T	T	C
78	STOX2	rs71622934	chr4:184831547	A	0.839	A	A	A	C	A	C	A	A	A	A	A	A	A	A	A	A			A	A	A	A
79	STOX2	rs2278475	chr4:184831715	A	23.892	G	A	A	A	G	A	A	A	G	A	A	A	G	A	G	A			G	A	G	A
80	STOX2	rs2278474	chr4:184831976	G	27.107	T	G	G	G	T	G	G	G	T	G	G	G	T	G	T	G	T	G	T	G	T	G
81	STOX2	rs113843232	chr4:184832443	C	1.358	C	C	C	C	C	C	C	T			C	T			C	C	C	C	C	T	C	C
82	STOX2	rs6552717	chr4:184832839	G	16.054	G	G	G	C	G	G	G	C	G	G	G	C	G	C	G	G	G	G	G	C	G	G
83	STOX2	rs28390196	chr4:184837323	T	33.546	T	C	T	C	T	T	C	C			C	C	T	C?	T	C	T	C	T	C	T	T
84	STOX2	rs13123798	chr4:184840588	G	47.903	G	G	C	C	G	C	G	G			G	G	G	C	G	C	G	C	G	C	C	C
85	STOX2	rs79173901	chr4:184840845	A	11.522	A	G	A	A	A	A	A	G	A	A	A	G	A	A	A	G	A	G	A	A	A	A
86	STOX2	rs6552718	chr4:184841089	G	47.045	A	G	A	G	A	G	A	A	G	G	A	A	A	G	A	G	A	A	A	G	A	G
87	STOX2	rs6552719	chr4:184841111	C	29.133	C	T	C	C	C	C	T	T	C	C	T	T	C	T	C	T	C	T	C	T	C	C
88	STOX2	rs11930663	chr4:184841784	T	11.502	T	T	T	T	T	T	T	T	T	T	T	T	T	T	T	T	T	T	T	T	T	T
89	STOX2	rs6818342	chr4:184841970	C	8.746	C	C	C	C	C	C	C	C	C	C	C	C	C	C	C	C	C	C	C	C	C	C
90	STOX2	rs6552720	chr4:184842016	C	19.988	C	C	C	C	C	C	C	C	C	C	C	C	C	C	C	C	C	T	C	C	C	T
91	STOX2	rs62339696	chr4:184842888	G	3.534	G	G	G	G	G	G	G	G	G	G	G	G	G	G			G	T	G	G	G	T
92	STOX2	rs144309617	chr4:184843229	T	0.599	T	T	T	T	T	T	T	T	T	T	T	T	T	T	T	T	T	T	T	T	T	T
93	STOX2	rs11735741	chr4:184843264	G	12.410	G	G	G	G	G	G	G	G	G	G	G	G	G	G	G	G	G	A	G	G	G	A
94	STOX2		chr4:184843281	G		G	G	G	G	G	G	G	G	G	G	G	G	G	G	G	G	G	A	G	G	G	A
95	STOX2	rs11735811	chr4:184843390	G	3.544	G	G	G	G	G	G	G	G	G	G	G	G	G	G	G	G	G	A	G	G	G	A
96	STOX2	rs187689954	chr4:184843593	G	6.530	G	G	G	G	G	G	G	G	G	G	G	G	G	G	G	G	G	A	G	G	G	A
97	STOX2	rs1009182	chr4:184845151	G		G	G	G	G	G	G	G	G	G	G	G	G			G	G	G	C	G	G	G	C
98	STOX2	rs145542601	chr4:184847485	TAAG		4	4	4	4	4	4	4	4	4	4	4	4	4	4	4	4	4	0	4	4	4	0
99	STOX2	rs17363081	chr4:184848958	G	14.237	G	G	G	A	G	A	G	A			G	A	G	A	G	G	G	G	G	A	G	G
100	STOX2	rs2309943	chr4:184849100	C	31.769	C	T	C	T	C	C	C	T	T	T	C	T	C	T	T	T	T	TT	C	T	T	T
101	STOX2	rs28667494	chr4:184850033	A	15.575	A	A	A	A	A	A	A	A	A	G	A	A	A	G	A	G			A	G	A	G
102	STOX2	rs6552721	chr4:184850894	A	25.020	A	A	A	A	A	A	A	A	A	A	A	A	A	A	A	A	A	A	A	A	A	A
103	STOX2	rs11722327	chr4:184851037	G	32.508	A	A	A	A	A	A	A	A	A	G	A	A	A	G	A	G	A	A	A	G	A	G
104	STOX2	rs6843949	chr4:184851058	C	7.049	C	C	C	C	C	C	C	C	C	T	C	C	C	T	C	T	C	C	C	T	C	T
105	STOX2	rs6821296	chr4:184851167	T	32.089	T	T	T	T	T	T	T	T	T	C	T	T	T	C	T	C	T	T	T	C	T	C
106	STOX2	rs6844718	chr4:184851233	G	30.332	G	G	G	G	G	G	G	G	G	A	G	G	G	A	G	A	G	G			G	A
107	STOX2	rs6844210	chr4:184851260	A	32.069	A	A	A	A	A	A	A	A	A	G	A	A	A	G	A	G	A	A			A	G
108	STOX2	rs6849976	chr4:184851713	A	32.548			A	A			A	A	A	G			A	G	A	G	A	A				
109	STOX2	rs28548141	chr4:184851808	C	31.030			C	C			C	C	C	A			C	A	C	A	C	C				
110	STOX2	rs4416517	chr4:184852306	G	25.080	G	G	G	G	G	G	G	G	G	A	G	G	G	A	G	A	G	G	G	A	G	A
111	STOX2	rs140012238	chr4:184852555	C	0.826	C	C	C	C	C	C	C	C	C	C	C	C	C	C	C	C	C	C	C	C	C	C
112	STOX2	rs6552722	chr4:184852627	A	44.229	A	C	A	A	A	A	A	C	A	C	A	C	A	C	C	C	A	A	A	C	A	C
113	STOX2	rs56125790	chr4:184852788	G	12.879	G	G	G	G	G	G	G	A	G	G	G	A	G	G	G	A	G	G	G	G	G	G
114	STOX2	rs6834333	chr4:184852809	T	49.780	C	C	C	C	C	C	C	T	C	T	C	T	C	T	T	T	C	C	C	T	C	T
115	STOX2	rs59778051	chr4:184854341	G	22.085	G	A	G	G	G	G	G	A	G	G	G	A	G	G	G	A	G	G	G	G	G	G
116	STOX2	rs10005362	chr4:184854611	G	15.295	G	G	G	G	G	G	G	G	G	A	G	G	G	A	G	A	G	G	G	A	G	A
117	STOX2	rs10005452	chr4:184854691	G	16.773	G	G	G	G	G	G	G	G	G	A			G	A	G	A	G	G			G	A
118	STOX2	rs35814015	chr4:184854724	T	48.383	C	C	C	C	C	C	C	T	C	T			C	T	T	T	C	C			C	T
119	STOX2	rs6828617	chr4:184855660	A	28.854	A	A	A	A	A	G	A	A	A	G	A	A	A	G	A	G	A	A	A	G	A	G
120	STOX2	rs1435438	chr4:184856061	A		A	A	A	A	A	A	A	A	A	G	A	A	A	G			A	A	A	G	A	G
121	STOX2	rs7673104	chr4:184856147	G	48.562	A	G	A	A	A	A	A	G	A	G	A	G	A	G	G	G	A	A	A	G		
122	STOX2	rs1435437	chr4:184856183	A		A	A	A	A	A	A	A	A	A	G	A	A	A	G	A	G	A	A	A	A		
123	STOX2	rs28427060	chr4:184857081	C	15.276	C	C	C	C	C	C	C	C	C	T	C	C	C	T	C	T	C	C	C	T	C	T
124	STOX2	rs28709244	chr4:184857329	T	27.316	T	T	T	T	T	T	T	T	T	C	T	T	T	C			T	T	T	C	T	C
125	STOX2	rs34489626	chr4:184857379	T	29.113	T	T	T	T	T	T	T	T	T	0	T	T	T	0			T	T	T	0	T	0
126	STOX2	rs2309944	chr4:184858078	T	37.380	T	C	T	T	T	T	C	C	T	T	C	C	T	C	T	C	T	T	T	C	T	T
127	STOX2	rs577329803	chr4:184873472	A	0.020	A	A	A	A	A	A	A	A	A	G	A	A	A	A	A	A	A	A	A	A	A	A
128	STOX2	rs111237279	chr4:184874118	A	10.024	A	A	A	A	A	A	A	A	A	A			A	A			A	A			A	A
129	STOX2	rs56111594	chr4:184874289	A	26.797	A	G	A	A	A	G	A	G	A	G			A	G			A	G			A	G
130	STOX2	rs55668413	chr4:184874343	A	31.270	T	A	T	T	T	A	T	T	T	A							T	A			T	A
131	STOX2	rs398093059	chr4:184874736	0		0	TT	0	0	0	TT	0	TT	0	TT	0	TT	0	TT	0	0	0	TT	0	TT	0	TT
132	STOX2	rs9990478	chr4:184874864	G	36.671	G	A	G	G	G	A	G	A	G	A	G	A	G	A	G	G	G	A	G	A	G	A
133	STOX2	rs2871385	chr4:184875120	C	40.715	C	A	C	C	C	A	C	A	C	A	C	A	C	A	C	C	C	A	C	A	C	A
134	STOX2	rs70959162	chr4:184875457	0		0	12	0	0	0	12	0	12	0	12	0	12	0	12	0	12	0	12	0	12	0	12
135	STOX2	rs2309954	chr4:184876270	A	23.682	A	G	A	A	A	G	A	G	A	G	A	G	A	G	A	A	A	G	A	G	A	G
136	STOX2	rs10213203	chr4:184876413	A	48.782	A	G	A	A	A	G	A	G	A	G	A	G	A	G	A	A	A	G	A	G	A	G
137	STOX2	rs77080998	chr4:184876690	G	1.358	G	G	G	G	G	G	G	G	G	G	G	G	G	G	G	G	G	G	G	G	G	G
138	STOX2	rs7672646	chr4:184877063	T	27.736			T	T																		
139	STOX2	rs66709966	chr4:184877118	AAGGC	27.716	5	0	5	5	5	0	5	0			5	0					5	0				
140	STOX2	rs9997017	chr4:184877141	C	23.922	C	T	C	C	C	T	C	T			C	T					C	T				
141	STOX2	rs7694569	chr4:184877142	C	27.676	C	G	C	C	C	G	C	G			C	G					C	G				
142	STOX2	rs367750917	chr4:184877596	ATCT		4	4	4	4	4	4	4	4	4	4	4	4	4	4	4	4	4	4	4	4	4	4
143	STOX2	rs397770516	chr4:184877636	ATCT		0	4	0	0	0	4	0	4	0	4	0	4	0	4	0	4	0	0	0	4	0	4
144	STOX2	rs6810800	chr4:184877945	C	28.914			C	C	C	T	C	T	C	T	C	T	C	T	C	C	C	T	C	T	C	T
145	STOX2	rs59920136	chr4:184878662	0		0	T	0	0	0	T	0	T	0	T	0	T	0	T	0	0	0	T	0	T	0	T
146	STOX2		chr4:184879007	C		C	C	C	C	C	C	C	C	C	C	C	C	C	C	C	C	C	C	C	C	C	C
147	STOX2	rs11937179	chr4:184879025	A	32.648	A	G	A	A	A	G	A	G	A	G	A	G	A	G	A	A	A	G	A	G	A	G
148	STOX2	rs11932973	chr4:184879091	T	23.442	T	C	T	T	T	C	T	C	T	C	T	C	T	C	T	T	T	C	T	C	T	C
149	STOX2	rs11946422	chr4:184879139	G	23.442	G	T	G	G	G	T	G	T	G	T	G	T	G	T	G	G	G	T	G	T	G	T
150	STOX2	rs10009132	chr4:184879281	A	27.456	A	G	A	A	A	G	A	G	A	G	A	G	A	G	A	A	A	G	A	G	A	G
151	STOX2	rs28683689	chr4:184879510	A	23.522	A	G	A	A	A	G	A	G	A	G	A	G	A	G	A	A	A	G	A	G	A	G
152	STOX2	rs11938281	chr4:184879552	A	24.561	A	C	A	A	A	C	A	C	A	C	A	C	A	C	A	A	A	C	A	C	A	C
153	STOX2	rs10001133	chr4:184879625	T	36.522	C	C	T	T	T	C	C	C	T	C	C	C	T	C	T	C	T	C	T	C	T	C
154	STOX2	rs67592505	chr4:184880496	T	10.943	T	C	T	T	T	C	T	C	T	C	T	C	T	C	C	C	T	T	T	C	T	C
155	STOX2	rs2309948	chr4:184881320	G	27.416			C	C			C	G			C	G	C	G	C	C	C	C	C	G		
156	STOX2	rs376458892	chr4:184882511	0		TGC	TGC	TGC	TGC	TGC	TGC	TGC	TGC	TGC	TGC	TGC	TGC	TGC	TGC	TGC	TGC	TGC	TGC	TGC	TGC	TGC	TGC
157	STOX2	rs9999145	chr4:184882782	G	30.851	G	A	G	G	G	A	G	A	G	A			G	A			G	G			G	G
158	STOX2	rs10014688	chr4:184884430	T	25.060	T	A	T	T	T	A	T	A	T	A	T	A	T	A	T	T	T	T	T	A	T	T
159	STOX2	rs57322041	chr4:184885664	0		0	0	0	0	0	0	0	0	0	0	0	0	0	0	0	0	0	0	0	0	0	0
160	STOX2	rs4861596	chr4:184886694	C	28.994	A	A			A	A	A	A	A	C	A	A			A	C	A	A	A	C	A	C
161	STOX2	rs59823756	chr4:184886845	G	4.812	G	G			G	G			G	G	G	G									G	G
162	STOX2	rs79228251	chr4:184887541	T	0.499	T	T	T	T	T	T	T	T	T	T	T	T	T	T	T	T	T	T	T	T	T	T
163	STOX2	rs6159576	chr4:184887678	C	0.907	C	C	C	C	C	C	C	C	C	C	C	C	C	C	C	C	C	C	C	C	C	C
164	STOX2	rs56869218	chr4:184887696	A	9.006	A	A	A	A	A	A	A	A	A	A	A	A	A	A	A	A	A	A	A	A	A	A
165	STOX2	rs73008328	chr4:184889808	C	2.476	C	C	C	C	C	C	C	C	C	C	C	C	C	C	C	C	C	C	C	C	C	C
166	STOX2	rs6835995	chr4:184890234	G	34.545	G	C	G	G	G	C	G	C	G	C	G	C	G	C	C	C	G	C	G	C	G	C
167	STOX2	rs73008329	chr4:184890289	G	2.236	G	G	G	G	G	G	G	G	G	G	G	G	G	G	G	G	G	G	G	G	G	G
168	STOX2	rs73008331	chr4:184890363	G	2.197	G	G			G	G	G	G	G	G	G	G	G	G	G	G	G	G	G	G	G	G
169	STOX2	rs11947559	chr4:184890393	G	4.513	G	G			G	G	G	G	G	G	G	G	G	G	G	G	G	G	G	G	G	G
170	STOX2	rs75284491	chr4:184891577	G	1.897	G	G	G	G	G	G	G	G	G	G	G	G	G	G	G	G	G	G	G	G	G	G
171	STOX2	rs73008339	chr4:184891609	T	4.653	T	T	T	T	T	T	T	T	T	T	T	T	T	T	T	T	T	T	T	T	T	T
172	STOX2	rs34759863	chr4:184891780	G	34.744	G	T	T	T	G	T	G	T	G	T	G	T	G	T	T	T	G	T	G	T	G	T
173	STOX2	rs73008340	chr4:184891949	G	4.673	G	G	G	G	G	G	G	G	G	G	G	G	G	G	G	G	G	G	G	G	G	G
174	STOX2	rs34475875	chr4:184892141	C	4.732	C	C	C	C	C	C	C	C	C	C	C	C	C	C	C	C	C	C	C	C	C	C
175	STOX2	rs115796794	chr4:184892180	T	1.897	T	T	T	T	T	T	T	T	T	T	T	T	T	T	T	T	T	T	T	T	T	T
176	STOX2	rs10020386	chr4:184892634	A	41.154	A	G	A	A	A	G	A	G	A	G	A	G	A	G	A	A	A	G	A	G	A	G
178	STOX2	SNP	chr4:184893833	A	4.540	A	A	A	A	A	A	A	A	A	A	A	A			A	A	A	A	A	A	A	A
179	STOX2	rs114468357	chr4:184895525	C	4.453	C	C	C	C	C	C	C	C	C	C	C	C	C	C	C	C	C	C	C	C	C	C
180	STOX2	rs55713442	chr4:184896587	C	10.823	C	C	C	C	C	C	C	C	C	C	C	C	C	C	C	C	C	C	C	C	C	C
181	STOX2	rs4241783	chr4:184899779	G	47.744	T	T	T	G	T	G							T	G	T	G			T	G	T	G
182	STOX2	rs35723722	chr4:184900053	T	18.490	T	T	T	A	T	A	T	T	T	A	T	T	T	A	T	A	T	A	T	A	T	A
183	STOX2	rs14146558	chr4:184900972	A	1.937	A	A	A	A	A	A	A	A	A	A	A	A	A	A	A	A	A	A	A	A	A	A
184	STOX2	rs10010202	chr4:184901873	G	8.147	G	G	G	G	G	G					G	G	G	G	G	G	G	G	G	G		
185	STOX2	rs2309949	chr4:184902533	G		A	A	A	G	A	G	A	A	A	G	A	A	A	G	A	G	A	G	A	G	A	G
186	STOX2	rs375398254	chr4:184905363	A		G	G			G	G	G	G	G	G			G	G	G	G					G	A
187	STOX2	rs4862279	chr4:184905482	C	22.264	C	A			C	A	C	A	C	A			C	A	C	C					C	A
188	STOX2	rs76111313	chr4:184906584	T	1.897	T	T	T	T	T	T	T	T	T	T	T	T	T	T	T	T	T	T	T	T	T	T
189	STOX2	rs28678779	chr4:184906606	C	8.127	C	C	C	C	C	C	C	C	C	C	C	C	C	C	C	C	C	C	C	C	C	C
190	STOX2	rs7685679	chr4:184906674	C	42.612	C	T	C	T	C	C	C	T	C	C	C	T	C	C	C	T	C	C	C	C	C	C
191	STOX2	rs28411264	chr4:184908447	A	6.250	A	A	A	A	A	A	A	A			A	A	A	A	A	A	A	A	A	A	A	A
192	STOX2	rs35046209	chr4:184909248	CC	21.286	CC	0	CC	0	CC	0	CC	CC			CC	CC	CC	0	CC	0	CC	0	CC	0	CC	0
193	STOX2	rs11731795	chr4:184910269	C	19.389	C	A	C	A	C	A	C	C			C	C	C	A	C	A	C	A	C	A	C	A
194	STOX2	rs13149152	chr4:184910896	T	20.407	T	T	T	A	T	A	T	T	T	A	T	T	T	A	T	A	T	A	T	A	T	A
195	STOX2	rs13122329	chr4:184910905	G	20.008	G	G	G	A	G	A	G	G	G	A	G	G	G	A	G	A	G	A	G	A	G	A
196	STOX2	rs4862280	chr4:184911938	A	25.699	G	G	G	G	G	G	G	G	G	G	G	G	G	G	G	G	G	G	G	G	G	G
197	STOX2	rs9312391	chr4:184913192	C	45.148	T	T	T	C	T	C	T	T	T	C	T	T	T	C	T	C	T	C	T	C	T	C
198	STOX2	rs9312320	chr4:184913291	G	24.720	G	T	G	G	G	T	G	T	G	T	G	T	G	T	G	G	G	T	G	T	G	T
199	STOX2	rs11735366	chr4:184913299	C	21.366	C	C	C	G	C	G	C	C	C	G	C	C	C	G	C	G	C	G	C	G	C	G
200	STOX2	rs11736295	chr4:184913507	G	21.326	G	G					G	G	G	A	G	G			G	A						
201	STOX2	rs146281849	chr4:184914457	A	0.120	A	A	A	A	A	A	A	A	A	A	A	A	A	A	A	A	A	A	A	A	A	A
202	STOX2	rs12651379	chr4:184914519	C	21.386	C	C	C	T	C	T	C	C	C	T	C	C	C	T	C	T	C	T	C	T	C	T
203	STOX2	rs28647598	chr4:184914536	C	44.808	G	G	G	C	G	C	G	G	G	C	G	G	G	C	G	C	G	C	G	C	G	C
204	STOX2	rs376210335	chr4:184914747	0		0	0	0	0	0	0	0	0	0	0	0	0	0	0	0	0	0	0	0	0	0	0
205	STOX2	rs73010232	chr4:184915818	C	20.867	C	T	C	T	C	C	C	T	C	C	C	T	C	C	C	T	C	C	C	C	C	C
206	STOX2	rs79225980	chr4:184916134	A	2.157	A	A	A	A	A	A	A	A	A	A	A	A			A	A	A	A	A	A	A	A
207	STOX2	rs6552726	chr4:184916993	C	44.010	G	G	G	C	G	C	G	G	G	C	G	G	G	C	G	C	G	C	G	C	G	C
208	STOX2	rs6552728	chr4:184917157	C	25.819	C	T	C	C	C	T	C	T	C	T	C	T	C	T	C	C	C	T	C	T	C	T
209	STOX2	rs76625198	chr4:184917179	G	0.639	G	G	G	G	G	G	G	G	G	G	G	G	G	G	G	G	G	A	G	G	G	G
210	STOX2	rs2309955	chr4:184917562	A	44.010	G	G	G	A	G	A	G	G	G	A	G	G	G	A	G	A	G	A	G	A	G	A
211	STOX2	rs12642003	chr4:184917791	G	21.645	G	G	G	A	G	A	G	G	G	A	G	G	G	A	G	A	G	A	G	A	G	A
212	STOX2		chr4:184918288	G		G	G	G	G	G	G	G	G	G	G	G	G	G	G	G	G	G	G	G	G	G	G
213	STOX2	rs17090673	chr4:184918852	G	21.306	G	G	G	A	G	A	G	G	G	A	G	G	G	A	G	A	G	A	G	A	G	A
214	STOX2	rs17090674	chr4:184919470	C	19.269	C	C	C	T	C	T	C	C	C	T	C	C	C	T	C	T	C	T	C	T	C	T
215	STOX2	rs10033149	chr4:184919684	C	42.133	T	C	C	C	T	C	T	C	T	C	T	C	T	C	C	C	T	C	T	C	T	C
216	STOX2	rs6835682	chr4:184920355	C	30.950	C	T	C	C	C	T	C	T	C	T	C	T	C	T	C	C	C	T	C	T	C	T
217	STOX2	rs131149751	chr4:184920382	A	19.349	A	A	A	C	A	C	A	A	A	C	A	A	A	C	A	C	A	C	A	C	A	C
218	STOX2	rs12498735	chr4:184921299	C	40.835	C	T	T	T	C	T	C	T	C	T	C	T	T	T	T	T	C	T	C	T	C	T
219	STOX2	rs17075203	chr4:184921471	G	2.157	G	G	G	G	G	G	G	G	G	G	G	G	G	G	G	G	G	G	G	G	G	G
220	STOX2	rs76113902	chr4:184921778	A	2.157	A	A	A	A	A	A	A	A	A	A	A	A	A	A	A	A	A	A	A	A	A	A
221	STOX2	rs72310239	chr4:184921915	TGT	41.314	TGT	0	0	0	TGT	0	TGT	0	TGT	0	TGT	0	TGT	0	0	0	TGT	0	TGT	0	TGT	0
222	STOX2	rs146806994	chr4:184921961	C	0.160	C	C	C	C	C	C	C	C	C	T	C	C			C	C	C	C	C	C	C	C
223	STOX2	rs7669647	chr4:184925597	A	38.518	G	A	G	A	G	G	G	A	G	G	G	A	G	G	G	A	G	G	G	G	G	G
224	STOX2	rs7694352	chr4:184925614	T	24.381	A	T	A	T	A	A	A	T	A	A	A	T	A	A	A	T	A	A	A	A	A	A
225	STOX2	rs7670377	chr4:184925740	G	35.563	G	G	G	A	G	A	G	G	G	A	G	G	G	A	G	A	G	A	G	A	G	A
226	STOX2	SNP	chr4:184925773	A		0	A	0	A	0	0	0	A	0	0	0	A	0	0	0	A	0	0	0	0	0	0
227	STOX2	rs6828396	chr4:184926387	C	37.700	T	C	C	C	T	T	T	C	T	T	T	C			T	C	T	T	T	T	T	T
228	STOX2	rs59029519	chr4:184926540?	0		G	0	0	0	G	G	G	0	G	G	G	0			0	0	G	G	G	G	G	G
229	STOX2	rs13147432	chr4:184926627	A	50.000					C	C			C	C			C	C	G		C	C	C	C	C	C
230	STOX2	rs67630164	chr4:184926673	TTTAAAA	36.122	0	7	0	7	0	0	0	7	0	0	0	7	0	0	0	7	0	0	0	0	0	0
231	STOX2	rs72697712	chr4:184928703	C	0.978	C	C	C	C	C	C	C	C	C	C	C	C	C	C	C	C	C	C	C	C	C	C
232	STOX2	rs72697714	chr4:184928929	T	1.018	T	T	T	T	T	T	T	T	T	T	T	T	T	T	T	T	T	T	T	T	T	T
233	STOX2	rs7655396	chr4:184928980	C	21.645	T	C	T	T	T	C	T	T	T	T	T	T	T	T	T	T	T	T	T	T	T	T
234	STOX2	rs114157769	chr4:184929455	T	2.296	T	T	T	T	T	T	T	T	T	T	T	T	T	T	T	T	T	T	T	T	T	T
235	STOX2	rs75082346	chr4:184929717	A	1.977	A	A	A	A	A	A	A	A	A	A	A	A	A	A	A	A	A	A	A	A	A	A
236	STOX2	rs28655984	chr4:184930023	G	19.529	G	G	G	G	G	G	G	G	G	G	G	G	G	G	G	G	G	G	G	G	G	G
237	STOX2	rs66511946	chr4:184932935	A	24.840	A	G	A	G	A	G	A	G			A	G	A	A	G	G	G	G	A	G	G	G
238	STOX2	rs6810667	chr4:184935603	C	41.254	C	C	C	T	C	C	C	T			C	T	C	T	C	C	C	T	C	T	C	T
239	STOX2	rs6836767	chr4:184935835	T	45.787	T	T	A	T	T	T	A	T			A	T	A	T	T	T	A	T	A	T	A	T
240	STOX2	rs112565268	chr4:184936024	T	2.596			T	T													T	T				
241	STOX2	rs11733294	chr4:184936136	C	42.772	T	T	C	T	T	T	C	T	C	T	C	T	C	T	T	T	C	T	C	T	C	T
242	STOX2	rs181346894	chr4:184936156	G	0.080	G	G	G	G	G	G	G	G	G	G	G	G	G	G	G	G	G	G	G	G	G	G
243	STOX2	rs3811778	chr4:184936547	T	21.336	C	C	T	C	C	C	T	C	T	C	T	C	T	C	C	C	T	C	T	C	T	C
244	STOX2		chr4:184936601	A		A	A	A	A	A	A	A	A	A	A	A	A	A	A	A	A	A	A	A	A	A	A
245	STOX2	rs3811777	chr4:184936604	A	21.895	A	A	A	G	A	A	A	G	A	G	A	G	A	G	A	A	A	G	A	G	A	G
246	STOX2	rs3811776	chr4:184936615	T	21.326	C	C	T	C	C	C	T	C	T	C	T	C	T	C	C	C	T	C	T	C	T	C
247	STOX2	rs3841107	chr4:184936677	TTTT		0	0	TTTT	0	0	0	TTTT	0	TTTT	0	TTTT	0	TTTT	0	0	0	TTTT	0	TTTT	0	TTTT	0
248	STOX2	rs3811775	chr4:184936790	G	21.296	A	A	G	A	A	A	G	A			G	A			A	A	G	A	G	A	G	A
249	STOX2	rs2309956	chr4:184937312	G	43.171	A	A	G	A	A	A	G	A			G	A	G	A	A	A	G	A	G	A	G	A
250	STOX2	rs2871386	chr4:184937365	T	42.712	C	C	T	C	C	C	T	C			T	C	T	C	C	C	T	C	T	C	T	C
251	STOX2	rs10026246	chr4:184937370	C	49.880	C	C	T	C	C	C	T	C			C	T	T	C	C	C	T	C	C	T	T	C
252	STOX2	rs62339733	chr4:184937736	C	13.538	C	A	C	C	C	A	C	C			C	C	C	C	C	C	C	C	C	C	C	C
253	STOX2	rs138948095	chr4:184938129	T	0.639	T	T	T	T	T	T	T	A			T	A			T	T	T	T	T	A	T	T

Suppl File 17B
Family 13

#	Gene	dbSNP 142	hg19	Ref	MAF	13		13		13		13		13		13		13		13		13		13		13		13		13		13	
						7290		7292		7291		7298		7307		7308		7294		7293		7301		7302		7296		7295		7303		7305	
1			chr4:184705736	G						G	T	G	G			G	G											G	G				
2			chr4:184705774	A						A	G	A	A			A	A											A	A				
3			chr4:184705807	C						C	A	C	C			C	C																
4		rs11356631	chr4:184705881	A	8.866					A	A	A	A			A	0																
5		rs62342249	chr4:184716354	A	20.607	A	A	A	A	A	A	A	A	A	A	A	G	A	A	A	G	A	A	A	G	A	A	A	A	A	A	A	A
6		rs138260968	chr4:184716514	TATT	1.857	4	4	4	0	4	4	4	4			4	4	4	0	4	4	0	0	4	4	4	0	4	4	4	0	4	4
7		rs60604513	chr4:184716840	T	15.695	T	C	T	T	T	C	C	C	T	T	T	C	T	C	T	T	T	T	T	T	T	T	T	C	T	C	T	C
8		rs60839040	chr4:184717536	C	23.542	C	C	C	C	C	C	C	C			C	A	C	C	C	A	C	C	C	A	C	C	C	C	C	C	C	C
9		rs367688566	chr4:184717537	20		20	20	20	20	20	20	20	20			20	0	20	20	20	20	20	20	20	20	20	20	20	20	20	20	20	20
10		rs62342250	chr4:184718319	T	17.792	T	T	T	T	T	C	T	T	T	T	T	T	T	T	T	C	T	C	T	T	T	T	T	T	T	T	T	T
11		rs62342251	chr4:184718676	C	16.454	C	T	C	T			T	T			C	T	C	T			C	C	C	T	C	T	C	T	C	C	C	T
12		rs75942758	chr4:184719247	A	18.091	A	A	A	C	A	C	A	A			A	A	A	C	A	C	C	C	A	A	A	C	A	A	A	C	A	A
13		rs67103969	chr4:184719252	A	41.713	G	A	A	A	A	A	A	A			G	A	A	A	G	A	A	A	G	A	A	A	G	A	G	A	G	A
14		rs146992605	chr4:184719283	C	2.276	C	C	C	C	C	C	C	C			C	A	C	C	C	C	C	C	C	C	C	C	C	A	C	A	C	A
15		rs116049039	chr4:184719284	C	2.276	C	C	C	C	C	C	C	C			C	A	C	C	C	C	C	C	C	C	C	C	C	A	C	A	C	A
16		SNP	chr4:184719654	C		C	C	C	C	C	C	C	C			C	T	C	C	C	C	C	C	C	C	C	C	C	C	C	C	C	C
17		rs72693687	chr4:184719992	T	7.668	T	T	T	T	T	T	T	T	T	C	T	T	T	T	T	T	T	T	T	T	T	T	T	C	T	C	T	C
18		rs78839475	chr4:184719996	C	7.648	C	C	C	C	C	C	C	C	C	C	C	C	C	C	C	C	C	C	C	C	C	C	C	G	C	G	C	G
19		SNP	chr4:184720045	T		16	16	16	16	16	16	16	16	16	25	16	25	16	16	16	25	16	16	16	25	16	16	25	25	16	25	16	25
20		rs9990869	chr4:184720139	C	38.478	C	C	C	T	C	T	C	C			C	C	C	T	C	T	T	T	C	C	T	T	C	T	T	T	C	T
21		rs72693688	chr4:184720199	C	2.117	C	C	C	C	C	T	C	C			C	C					C	T			C	C	C	C				
22		rs11722400	chr4:184727641	A	36.921	A	A	A	A	A	C					A	C	A	A	A	C	A	C	A	A	A	A	A	A	A	A	A	A
23		rs4078180	chr4:184744832	C	17.951			C	C			C	C			C	C											C	G			C	C
24		rs4078181	chr4:184744873	G	14.357			G	G			G	G			G	G											G	C			G	G
25			chr4:184749538	C		C	C	C	C	C	C	C	C			C	C	C	C	C	C	C	C	C	C	C	C	C	C	C	C	C	C
26			chr4:184749921	A		A	A	A	A	A	A	A	A			A	A	A	A	A	A	A	A	A	A	A	A	A	A	A	A	A	A
27		rs75766856	chr4:184749987	G	1.857	G	A									G	A																
28		rs149453332	chr4:184753805	G	2.097	G	G	G	G	G	G	G	G			G	G	G	G	G	A	G	A	G	G	G	G	G	G			G	G
29		rs140928377	chr4:184770875	G	1.777	G	G	G	A	G	G	G	G	G	G	G	G	G	A	G	G	G	A	G	G	G	A	G	G	G	A	G	G
30		rs6813846	chr4:184770923	G	34.565	G	G	G	G	G	G	G	G	G	G	G	G	G	G	G	A	G	A	G	G	G	G	A	A	G	A	G	A
31		rs62340391	chr4:184773893	A	42.372	A	G			G	G	A	A			G	G	A	G	A	G	A	A	A	G					A	A	A	G
32		rs6840225	chr4:184774772	A	40.196	A	G	A	G	A	A	A	A	A	A	A	G	A	G	A	G	G	G	A	A	A	G	A	G	A	G	A	A
33		rs56348474	chr4:184776401	T	38.978	T	C	T	C	T	T	T	T			T	C	T	C	T	C	C	C	T	T	T	C	T	C	T	C	T	T
34		rs2033626	chr4:184776546	G		G	C	G	G	G	G	G	G			G	C	G	G	G	C	G	C	G	G	G	G	C	C	G	C	G	C
35		rs9997501	chr4:184776644	C	40.236	C	T	C	T	C	C	C	C			C	T	C	T	C	T	T	T	C	C	C	T	C	T	C	T	C	C
36		rs147162710	chr4:184776666	A	0.160	A	A	A	A	A	A	A	A			A	A	A	A	A	A	A	A	A	A	A	A	A	A	A	A	A	A
37		rs6552712	chr4:184779462	C	44.010	C	T	C	T	T	T			C	T	C	T	C	T	C	T	C	C	T	T	C	T	C	C	C	C	C	T
38		rs12499183	chr4:184780717	C	39.497	C	G	C	G	C	C	C	C	C	G	C	G	C	G	C	G	G	G	C	C	C	G	C	G	C	G	C	C
39		rs892838	chr4:184780272	T				T	G	T	T	T	T	T	G	T	G	T	G	T	G	G	G	T	T	T	G	T	G	T	G	T	T
40		rs6852989	chr4:184781947	G	8.846	T	T	T	T	T	T	T	T	T	T	T	T	T	T	T	T	T	T	T	T	T	T			T	G	T	G
41		rs11944432	chr4:184797519	T	34.105	A	T	A	T	A	T	A	T	A	A	A	T	A	T	T	T	T	T	A	T	A	T	T	T	T	T	A	T
42			chr4:184797521	G		G	C	G	G	G	G	G	G	G	G	G	G	G	G	G	G	G	G	G	G			G	G	G	G	G	G
43		rs78932922	chr4:184797708	G	0.679	G	G	G	G	G	G	G	G	G	G	G	G	G	G	G	G	G	G	G	G			G	A	G	A	G	A
44		rs34608496	chr4:184797917	C	28.894	C	T	C	T	C	T	C	T			C	T	C	T	T	T	T	T	C	T	C	T			T	T	C	T
45		rs13134353	chr4:184798026	T	28.994	T	G	T	G	T	G	T	G			T	G	T	G	G	G	G	G	T	G	T	G			G	G	T	G
46		rs7686859	chr4:184798422	C	49.341	A	C	A	C	A	C	A	C			A	C	A	C	C	C	C	C	A	C	A	C			C	C	A	C
47		rs6813664	chr4:184788700	A	34.156	G	A	G	A	G	A	G	A			G	A	G	A	A	A	A	A	G	A	G	A	A	A	A	A	G	A
48		rs4146058	chr4:184798737	G	33.127	A	G	A	G	A	G	A	G			A	G	A	G	G	G	G	G	A	G	A	G	G	G	G	G	A	G
49		rs2164301	chr4:184798865	G	29.213	G	A	G	A	G	A	G	A			G	A	G	A	A	A	A	A	G	A	G	A	A	A	A	A	G	A
50		rs7695698	chr4:184801157	T	22.005	T	G	T	T	T	G	T	G	G	G	T	G	T	G	G	G			T	G	T	G	T	G	T	G	T	T
51		SNP	chr4:184807252	G		G	G	G	G	G	G	G	G	G	G	G	G	G	G	G	G	G	G			G	G	G	G	G	G	G	G
52		rs372520827	chr4:184807256	G		G	C	G	C	G	C	G	C	G	G	G	G	G	C	C	C	C	C			G	C	C	C	C	C	G	C
53		rs376044001	chr4:184807257	A		A	T	A	T	A	T	A	T	A	A	A	A	A	T	T	T	T	T			A	T	T	T	T	T	A	T
54		rs71622933	chr4:184807283	A	50.000	A	C	A	C	A	C	A	C	A	A	A	A	A	C	C	C	C	C			A	C	C	C	C	C	A	C
55		rs12649564	chr4:184807335	C	50.000	A	C	A	C	A	C	A	C	A	A	A	A	A	C	C	C	C	C			A	C	C	C	C	C	A	C
56		rs12649564	chr4:184807396	C	42.857	C	T	C	T	C	T	C	T	C	C	C	C	C	T	T	T	T	T			C	T	T	T	T	T	C	T
57		SNP	chr4:184807491	C		A	C	A	C	A	C	A	C	A	A	A	A	A	C	C	C	C	C			A	C	C	C	C	C	A	C
58		SNP	chr4:184807500	A		T	C	T	C	T	C	T	C	T	T	T	T	T	C	C	C	C	C			T	C	C	C	C	C	T	C
59		rs398083846	chr4:184807508	G																													
60		rs181100773	chr4:184807730	T	0.020	T	T	T	T	T	T	T	T	T	T	T	T	T	T	T	T	T	T			T	T	T	G	T	G	T	G
61		rs4990039	chr4:184811610	T	29.972	C	T	C	T	C	C	C	C			C	C	C	T	C	T	T	T	C	C	C	T			T	T	C	T
62		rs13130260	chr4:184824352	G	31.889	G	G	G	G	G	A	G	A	G	G	G	G	G	G	G	A	G	G	G	A	G	G	G	A	G	G	G	G
63		rs114894140	chr4:184824378	A	1.338	A	A	A	A	A	A	A	A	A	A	A	A	A	A	A	A	A	A	A	A	A	A	A	A	A	A	A	A
64		rs188804673	chr4:184824996	G	0.240	G	G			G	G	G	G	G	G	G	G	G	G	G	G	G	G	G	G	G	G	G	G	G	G	G	G
65		rs10017668	chr4:184825092	A	21.486	A	A	A	A	A	A	A	A			A	A	A	A	A	A	A	A	A	A	A	A	A	T	A	T	A	T
66		rs10005808	chr4:184825170	C	21.545	T	T	T	T	T	T	T	T			T	T	T	T	T	T	T	T	T	T	T	T	T	C	T	C	T	C
67	STOX2	rs17075136	chr4:184825696	A	29.513	A	A	A	A	A	G	A	G	A	A	A	A	A	A	A	G	A	A	A	G	A	A			A	A	A	A
68	STOX2	rs1560982	chr4:184826026	G	22.624			A	A			G	A	G	A	G	A									A	A			G	A	G	A
69	STOX2	rs36115739	chr4:184826381	A	48.403	T	T	T	T	A	T	A	T			T	T	T	T	T	T	T	T	A	T	T	T			A	T	A	T
70	STOX2	rs62339673	chr4:184828533	C	49.720	A	A	A	A	A	C	A	C			A	A	A	A	A	C	A	A	A	C	A	A			A	C	A	C
71	STOX2	rs7689460	chr4:184830322	T	35.643	T	T	T	T	G	G	T	G			T	T	T	T	T	G	T	T	T	G	T	T	T	G			T	T
72	STOX2	rs112673876	chr4:184830631	C	5.032	C	C	C	C	C	C	C	C			C	C	C	C	C	C	C	C	C	C	C	C	C	G			C	G
73	STOX2	rs12647459	chr4:184830682	T	35.982	T	T	T	T	G	G	T	G			T	T	T	T	T	G	T	T	T	G	T	T	T	G			T	T
74	STOX2	rs12644266	chr4:184830847	C	35.963	C	C	C	C	T	T	C	T			C	C	C	C	C	T	C	C	C	T	C	C	C	T	C	C	C	C
75	STOX2	rs113262003	chr4:184830913	0	35.783	0	0	0	0	4	4	0	4			0	0	0	0	0	4	0	0	0	4	0	0	0	4	0	0	0	0
76	STOX2	rs4862265	chr4:184831428	C	49.002	T	T	T	T	C	C	T	C			T	T	T	T	T	C	T	T	T	C	T	T					T	C
77	STOX2	rs4862266	chr4:184831454	C	42.732	T	T	T	T	C	C	T	C			T	T	T	T	T	C	T	T	T	C	T	T					T	C
78	STOX2	rs71622934	chr4:184831547	A	0.839	A	A	A	A	A	C	A	C			A	A	A	A	A	C	A	A	A	C	A	A					A	A
79	STOX2	rs2278475	chr4:184831715	A	23.892	G	G	G	G	A	A	G	A			G	G	G	G	G	A	G	G	G	A	G	G					G	A
80	STOX2	rs2278474	chr4:184831976	G	27.107	T	T	T	T	G	G	T	G			T	T	T	T	T	G	T	T	T	G	T	T	T	G	T	G	T	G
81	STOX2	rs113843232	chr4:184832443	C	1.358			C	C	C	C	C	C			C	C	C	C	C	C	C	C			C	C	C	C	C	C	C	C
82	STOX2	rs6552717	chr4:184832839	G	16.054	G	G	G	G	G	G	G	G			G	G	G	G	G	G	G	G	G	G	G	G	G	C	G	C	G	C
83	STOX2	rs28390196	chr4:184837323	T	33.546	T	T	T	T	T	C	T	T			T	T	T	T	T	C	T	C	T	T	T	T			T	T	T	T
84	STOX2	rs13123798	chr4:184840588	G	47.903	C	C	C	C	C	C	C	C	G?	C	C	C	C	C	G	C	G	C	C	C	C	C			G	C	G	C
85	STOX2	rs79173901	chr4:184840845	A	11.522	A	A	A	A	A	A	A	A			A	A	A	A	A	G	A	G	A	A	A	A	A	A	A	A	A	A
86	STOX2	rs6552718	chr4:184841089	G	47.045	G	G	G	G	G	A	G	A			G	A	G	G	G	A	G	A	G	G	G	G	G	G	A	G	A	G
87	STOX2	rs6552719	chr4:184841111	C	29.133	C	C	C	C	C	C	C	C			C	C	C	C	C	T	C	T	C	C	C	C	C	C	C	C	C	C
88	STOX2	rs11930663	chr4:184841784	T	11.502	T	T	T	T	T	T	T	T			T	T	T	T	T	T	T	T	T	T	T	T	T	G	T	G	T	G
89	STOX2	rs6818342	chr4:184841970	C	8.746	C	C	C	C	C	C	C	C			C	C	C	C	C	C	C	C	C	C	C	C	C	T	C	T	C	T
90	STOX2	rs6552720	chr4:184842016	C	19.988	C	C	C	C	C	C	C	C			C	C	C	C	C	C	C	C	C	C	C	C	C	T	C	T	C	T
91	STOX2	rs62339696	chr4:184842888	G	3.534	G	G	G	G	G	G	G	G			G	G	G	G	G	G	G	G	G	G	G	G	G	G	G	G	G	G
92	STOX2	rs144309617	chr4:184843229	T	0.599	T	T	T	T	T	T	T	T			T	G	T	T	T	T	T	T	T	T	T	T	T	T	T	T	T	T
93	STOX2	rs11735741	chr4:184843264	G	12.410	G	G	G	G	G	G	G	G			G	G	G	G	G	G	G	G	G	G	G	G	G	G	G	G	G	G
94	STOX2		chr4:184843281	G		G	G	G	G	G	G	G	G			G	G	G	G	G	G	G	G	G	G	G	G	G	G	G	G	G	G
95	STOX2	rs11735811	chr4:184843390	G	3.544	G	G	G	G	G	G	G	G			G	G	G	G	G	G	G	G	G	G	G	G	G	G	G	G	G	G
96	STOX2	rs187689954	chr4:184843593	G	6.530					G	G	G	G			G	G	G	G	G	G	G	G	G	G	G	G	G	G	G	G	G	G
97	STOX2	rs1009182	chr4:184845151	G		G	G			G	G	G	G			G	G	G	G					G	G	G	G	G	G	G	G		
98	STOX2	rs145542601	chr4:184847485	TAAG	13.079	4	4	4	4	4	4	4	4			4	4	4	4	4	4	4	4	4	4	4	4	4	4	4	4	4	4
99	STOX2	rs17363081	chr4:184848958	G	14.237	G	G	G	G	G	A	G	A	G	A	G	A	G	G	G	G	G	G	G	G	G	G			G	G	G	G
100	STOX2	rs2309943	chr4:184849100	C	31.769	T	T	C	T	C	T	C	T			C	T	C	T	T	T	C	T	T	T	C	T	T	T	C	T	T	T
101	STOX2	rs28667494	chr4:184850033	A	15.575			A	A	A	A	A	A			A	A	A	A	A	A	A	A	A	A	A	A	A	A	A	A	A	A
102	STOX2	rs6552721	chr4:184850894	A	25.020	A	A	A	A	A	A	A	A	A	G	A	G	A	A	A	A	A	A	A	A	A	A	A	G	A	A	A	A
103	STOX2	rs11722327	chr4:184851037	G	32.508	A	A	A	A	A	A	A	A	A	A	A	A	A	A	A	A	A	A	A	A	A	A	A	G	A	A	A	A
104	STOX2	rs6843949	chr4:184851058	C	7.049	C	C	C	C	C	C	C	C	C	C	C	C	C	C	C	C	C	C	C	C	C	C	C	C	C	C	C	C
105	STOX2	rs6821296	chr4:184851167	T	32.089	T	T	T	T	T	T	T	T	T	T	T	T	T	T	T	T	T	T	T	T	T	T	T	C	T	T	T	T
106	STOX2	rs6844718	chr4:184851233	G	30.332	G	G	G	G	G	G	G	G			G	G	G	G	G	G	G	G	G	G	G	G					G	G
107	STOX2	rs6844210	chr4:184851260	A	32.069	A	A	A	A	A	A	A	A			A	A	A	A	A	A	A	A	A	A	A	A					A	A
108	STOX2	rs6849976	chr4:184851713	A	32.548	A	A			A	A	A	A			A	A			A	A	A	A	A	A	A	A			A	A	A	A
109	STOX2	rs28548141	chr4:184851808	C	31.030	C	C			C	C	C	C			C	C							C	C	C	C			C	C	C	C
110	STOX2	rs4416517	chr4:184852306	G	25.080	G	G	G	G	G	G	G	G			G	G	G	G	G	G	G	G	G	G	G	G	G	A	G	G	G	G
111	STOX2	rs140012238	chr4:184852555	C	0.826	C	C	C	C	C	C	C	C	T	T	C	T	C	C	C	C	C	C	C	C	C	C	C	C	C	C	C	C
112	STOX2	rs6552722	chr4:184852627	A	44.229	A	A	A	C	A	A	A	A	A	A	A	A	A	C	A	A	A	C	A	A	A	A	A	C	A	C	A	A
113	STOX2	rs56125790	chr4:184852788	G	12.879	G	G	G	A	G	G	G	G			G	G	G	A	G	G	G	A			A	A	G	G	G	A	G	A
114	STOX2	rs6834333	chr4:184852809	T	49.780	C	C	C	T	C	C	C	C			C	C	C	T	C	C	C	T			T	T	C	T	C	T	C	C
115	STOX2	rs59778051	chr4:184854341	G	22.085	G	G	G	A	G	G	G	G			G	G	G	A	G	G	G	A	G	G	A	A	G	G				
116	STOX2	rs10005362	chr4:184854611	G	15.295	G	G	G	G	G	G	G	G			G	G	G	G	G	G	G	G	G	G	G	G	G	A	G	G	G	G
117	STOX2	rs10005452	chr4:184854691	G	16.773	G	G	G	G	G	G	G	G			G	G	G	G	G	G	G	G	G	G	G	G	G	A	G	G	G	G
118	STOX2	rs35814015	chr4:184854724	T	48.383	C	C	C	C	C	C	C	C			C	C	C	T	C	C	C	T	C	C	T	T	C	T	C	T	C	C
119	STOX2	rs6828617	chr4:184855660	A	28.854	A	A	A	A	A	A	A	A			A	A	A	A	A	A	A	A	A	A	A	A	A	G	A	A	A	A
120	STOX2	rs1435438	chr4:184856061	A						A	A	A	A			A	A	A	A	A	A	A	A	A	A	A	A	A	G	A	A	A	A
121	STOX2	rs7673104	chr4:184856147	G	48.562	A	A	A	G	A	A	A	A			A	A	A	G	A	A	A	G	A	A	G	G	A	G	A	G	A	A
122	STOX2	rs1435437	chr4:184856183	A		A	A	A	A	A	A	A	A			A	A	A	A	A	A	A	A	A	A	A	A	A	A	A	A	A	A
123	STOX2	rs28427060	chr4:184857081	C	15.276	C	C	C	C	C	C	C	C			C	C	C	C	C	C	C	C	C	C	C	C	C	T	C	C	C	C
124	STOX2	rs28709244	chr4:184857329	T	27.316	T	T	T	T	T	T	T	T			T	T	T	T	T	T	T	T	T	T	T	T	T	C	T	T	T	T
125	STOX2	rs34489626	chr4:184857379	T	29.113	T	T	T	T	T	T	T	T			T	T	T	T	T	T	T	T			T	T	T	0	T	T	T	
126	STOX2	rs2309944	chr4:184858078	T	37.380	T	T	T	C	T	T	T	T	T	T			T	C	T	T			T	T	C	C	T	T	T	C	T	T
127	STOX2	rs577329803	chr4:184873472	A	0.020	A	A	A	A	A	A	A	A			A	A	A	A	A	A	A	A	A	A	A	A	A	A	A	A	A	A
128	STOX2	rs111237279	chr4:184874118	A	10.024	A	G	A	A	A	A	A	A			A	A			A	A	A	A	A	A	A	A	A	A	A	A	A	A
129	STOX2	rs56111594	chr4:184874289	A	26.797	A	G	A	G	A	G	A	G			G	G			A	G	A	A	G	G	A	G	A	G	A	A	A	G
130	STOX2	rs55668413	chr4:184874343	A	31.270	T	A	T	A	T	A	T	A			A	A					T	T	A	A	T	A	T	A	T	T	T	A
131	STOX2	rs398093059	chr4:184874736	0		0	TT	0	TT	0	TT	0	TT			0	TT	0	TT	0	TT	0	0	0	TT	0	TT	0	TT	0	TT	0	TT
132	STOX2	rs9990478	chr4:184874864	G	36.671	G	A	G	G	G	A	G	A			A	A	G	A			G	G	A	A	G	A	G	A	G	G	G	A
133	STOX2	rs2871385	chr4:184875120	C	40.715	C	A	C	C	C	A	C	A			A	A	C	A			C	C	A	A	C	A	C	A	C	C	C	A
134	STOX2	rs70959162	chr4:184875457	0		0	12	0	12	0	12	0	12			12	12	0	12	0	12	0	0	12	12	0	12	0	12	0	0	0	12
135	STOX2	rs2309954	chr4:184876270	A	23.682	A	G	A	G	A	G	A	G			G	G	A	G	A	G	A	A	G	G	G	G	A	G	A	A	A	G
136	STOX2	rs10213203	chr4:184876413	A	48.782	G	G	A	G	A	G	A	G			G	G	A	G	A	G	A	A	G	G	A	G	A	G	A	A	A	G
137	STOX2	rs77080998	chr4:184876690	G	1.358	G	G	G	G	G	G	G	G			G	A	G	G	G	G	G	G	G	G	G	G	G	G	G	G	G	G
138	STOX2	rs7672646	chr4:184877063	T	27.736											G	G					T	T	G	G					T	T		
139	STOX2	rs66709966	chr4:184877118	AAGGC	27.716	5	0	5	0	5	0	5	0	5	5	0	0	5	0	5	0	5	5	0	0	5	0	5	0	5	5	5	0
140	STOX2	rs9997017	chr4:184877141	C	23.922	C	T	C	T	C	T	C	T	C	C	T	T	C	T	C	T	C	C	T	T	C	T	C	T	C	C	C	T
141	STOX2	rs7694569	chr4:184877142	C	27.676	C	G	C	G	C	G	C	G	C	C	G	G	C	G	C	G	C	C	G	G	C	G	C	G	C	G	C	G
142	STOX2	rs367750917	chr4:184877596	ATCT		4	4	4	4	4	4	4	4			0	0	4	4	4	4	4	4	0	0	4	4	4	4	4	4	4	4
143	STOX2	rs397770516	chr4:184877636	ATCT		0	4	0	4	0	4	0	4			0	0	0	4	0	4	0	0	0	0					0	4		
144	STOX2	rs6810800	chr4:184877945	C	28.914	C	T	T	T	C	T	C	T			T	T	C	T	C	T	C	C	T	T	C	T	C	T	C	C	C	T
145	STOX2	rs59920136	chr4:184878662	0		0	T	0	T	0	T	0	T			T	T	0	T	0	T	0	0	T	T	0	T	0	T	0	0	0	T
146	STOX2		chr4:184879007	C		C	C	C	T	C	C	C	C			C	C	C	T	C	C	C	T	C	C	C	T	C	C	C	T	C	C
147	STOX2	rs11937179	chr4:184879025	A	32.648	A	G	A	G	A	G	A	G			G	G	A	G	A	G	A	A	G	G	A	G	A	G	A	A	A	G
148	STOX2	rs11932973	chr4:184879091	T	23.442	T	C	T	C	T	C	T	C			C	C	T	C	T	C	T	T	C	C	T	C	T	C	T	T	T	C
149	STOX2	rs11946422	chr4:184879139	G	23.442	G	T	G	T	G	T	G	T			T	T	G	T	G	T	G	G	T	T	G	T	G	T	G	G	G	T
150	STOX2	rs10009132	chr4:184879281	A	27.456	A	G	A	G	A	G	A	G			G	G	A	G	A	G	A	A	G	G	A	G	A	G	A	A	A	G
151	STOX2	rs28683689	chr4:184879510	A	23.522	A	G	A	G	A	G	A	G			G	G	A	G	A	G	A	A	G	G	A	G	A	G	A	A	A	G
152	STOX2	rs11938281	chr4:184879552	A	24.561	A	C	C	C	A	C	A	C			C	C	A	C	A	C	A	A	C	C	A	C	A	C	A	A	A	C
153	STOX2	rs10001133	chr4:184879625	T	36.522	T	C	T	C	T	C	T	C			C	C	T	C	T	C	T	T	C	C	T	C	T	C	T	T	T	C
154	STOX2	rs67592505	chr4:184880496	T	10.943	T	T	T	T	T	T	T	T			T	T	T	T	T	T	T	T	T	T	T	T	T	T	T	T	T	T
155	STOX2	rs2309948	chr4:184881320	G	27.416	G	G			C	G	C	G	C	G			C	G	C	G	C	C	G	G	C	G			C	C	C	G
156	STOX2	rs376458892	chr4:184882511	0		TGC	0	TGC	0	TGC	TGC	TGC	0	TGC	TGC	TGC	0	TGC	0	TGC	TGC	TGC	TGC	TGC	0	TGC	0	TGC	TGC	TGC	TGC	TGC	0
157	STOX2	rs9999145	chr4:184882782	G	30.851	A	A	G	A	G	A							G	A	G	A	G	G	A	A			G	A	G	G		
158	STOX2	rs10014688	chr4:184884430	T	25.060	T	A	T	A	T	A	T	A			A	A	T	A	T	A	T	T	A	A	T	A	T	A	T	T	T	A
159	STOX2	rs57322041	chr4:184885664	0		0	ATTG	0	0	0	0	0	0			0	0	0	0	0	0	0	0	0	0	0	0	0	0	0	0	0	0
160	STOX2	rs4861596	chr4:184886694	C	28.994	A	A	A	A	A	A	A	A			A	A	A	A	A	A	A	A	A	A	A	A	A	A	A	A	A	A
161	STOX2	rs59823756	chr4:184886845	G	4.812	G	T	G	G	G	G	G	G			G	G	G	G			G	G	G	G	G	G	G	G	G	G	G	G
162	STOX2	rs79228251	chr4:184887541	T	0.499	T	C	T	T	T	T	T	T			T	T	T	T	T	T	T	T	T	T	T	T	T	T	T	T	T	T
163	STOX2	rs6159576	chr4:184887678	C	0.907	C	T	C	C	C	C	C	C			C	C	C	C	C	C	C	C	C	C	C	C	C	C	C	C	C	C
164	STOX2	rs56869218	chr4:184887696	A	9.006	A	G	A	A	A	A	A	A			A	A	A	A	A	A	A	A	A	A	A	A	A	A	A	A	A	A
165	STOX2	rs73008328	chr4:184889808	C	2.476	C	G	C	C	C	C	C	C			C	C	C	C	C	C	C	C	C	C	C	C	C	C	C	C	C	C
166	STOX2	rs6835995	chr4:184890234	G	34.545	G	G	G	C	G	C	G	C			G	G	G	C	G	C	C	C			G	C	C	C	C	C	G	C
167	STOX2	rs73008329	chr4:184890289	G	2.236	G	A	G	G	G	G	G	G			G	G	G	G	G	G	G	G			G	G	G	G	G	G	G	G
168	STOX2	rs73008331	chr4:184890363	G	2.197	G	A	G	G	G	G	G	G			G	G	G	G	G	G	G	G			G	G	G	G	G	G	G	G
169	STOX2	rs11947559	chr4:184890393	G	4.513	G	T	G	G	G	G	G	G			G	G	G	G	G	G	G	G	G	G	G	G	G	G	G	G	G	G
170	STOX2	rs75284491	chr4:184891577	G	1.897	G	A	G	G	G	G	G	G			G	G	G	G	G	G	G	G	G	G	G	G	G	G	G	G	G	G
171	STOX2	rs73008339	chr4:184891609	T	4.653	T	C	T	T	T	T	T	T			T	T	T	T	T	T	T	T	T	T	T	T	T	T	T	T	T	T
172	STOX2	rs34759863	chr4:184891780	G	34.744	G	G	G	T	G	T	G	T			G	G	G	T	G	T	T	T	G	G	G	T	T	T	T	T	G	T
173	STOX2	rs73008340	chr4:184891949	G	4.673	G	A	G	G	G	G	G	G			G	G	G	G	G	G	G	G	G	G	G	G	G	G	G	G	G	G
174	STOX2	rs34475875	chr4:184892141	C	4.732	C	T	C	C	C	C	C	C			C	C	C	C	C	C	C	C	C	C	C	C	C	C	C	C	C	C
175	STOX2	rs115796794	chr4:184892180	T	1.897	T	C	T	T	T	T	T	T			T	T	T	T	T	T	T	T	T	T	T	T	T	T	T	T	T	T
176	STOX2	rs10020386	chr4:184892634	A	41.154	G	G	A	G	A	G	A	G			G	G	A	G	A	G	A	A	G	G	A	G	G	G	A	G	A	G
178	STOX2	SNP	chr4:184893833	A	4.540	A	A	A	A	A	A	A	A			A	0	A	A	A	A	A	A	A	A	A	A	A	A	A	A	A	A
179	STOX2	rs114468357	chr4:184895525	C	4.453	C	T	C	C	C	C	C	C			C	C	C	C	C	C	C	C	C	C	C	C	C	C	C	C	C	C
180	STOX2	rs55713442	chr4:184896587	C	10.823	C	G	C	C	C	C	C	C			C	C	C	C	C	C	C	C	C	C	C	C	C	C	C	C	C	C
181	STOX2	rs4241783	chr4:184899779	G	47.744	T	G			T	T					T	T	T	T	T	G	T	G	T	T			G	G	T	G	T	G
182	STOX2	rs35723722	chr4:184900053	T	18.490	T	T	T	T	T	T	T	T			T	T	T	T	T	A	T	A	T	T	T	T	A	A	T	A	T	A
183	STOX2	rs14146558	chr4:184900972	A	1.937	A	C	A	A	A	A	A	A			A	A	A	A			A	A			A	A	A	A	A	A	A	A
184	STOX2	rs10010202	chr4:184901873	G	8.147	G	T	G	G	G	G	G	G			G	G	G	G	G	G			G	G	G	G	G	G	G	G	G	G
185	STOX2	rs2309949	chr4:184902533	G		A	G	A	A	A	A					A	A	A	A	A	G	A	G	A	A								
186	STOX2	rs375398254	chr4:184905363	A								G	G															G	G			G	G
187	STOX2	rs4862279	chr4:184905482	C	22.264							C	A															C	C			C	A
188	STOX2	rs76111313	chr4:184906584	T	1.897	T	G	T	T	T	T	T	T			T	T	T	T	T	T	T	T	T	T	T	T	T	T	T	T	T	T
189	STOX2	rs28678779	chr4:184906606	C	8.127	C	T	C	C	C	C	C	C			C	C	C	C	C	C	C	C	C	C	C	C	C	C	C	C	C	C
190	STOX2	rs7685679	chr4:184906674	C	42.612	C	C	C	T	C	T	C	T			C	C	C	T	C	C	C	T	C	C	C	T	C	C			C	C
191	STOX2	rs28411264	chr4:184908447	A	6.250	A	G	A	A	A	A	A	A			A	A	A	A	A	A	A	A	A	A	A	A			A	A	A	A
192	STOX2	rs35046209	chr4:184909248	CC	21.286	CC	CC	CC	CC	CC	CC	CC	CC	0	0	CC ?	CC ?	CC	CC	CC	CC	CC	CC	CC	CC	CC	CC	0	0	CC	0	CC	0
193	STOX2	rs11731795	chr4:184910269	C	19.389	C	C	C	C	C	C	C	C			C	C	C	C	C	C	C	C	C	C	C	C			C	A	C	A
194	STOX2	rs13149152	chr4:184910896	T	20.407	T	T	T	T	T	T	T	T	T	T	T	T	T	T	T	T	T	T	T	T					T	A	T	A
195	STOX2	rs13122329	chr4:184910905	G	20.008	G	G	G	G	G	G	G	G	G	G	G	G	G	G	G	G	G	G	G	G					G	A	G	A
196	STOX2	rs4862280	chr4:184911938	A	25.699	G	A	G	G	G	G	G	G	G	G	G	G	G	G	G	G	G	G	G	G	G	G	G	G	G	G	G	G
197	STOX2	rs9312391	chr4:184913192	C	45.148	T	C	T	T	T	T	T	T			T	T	T	T	T	T	T	T	T	T	T	T	C	C	T	C	T	C
198	STOX2	rs9312320	chr4:184913291	G	24.720	G	T	T	T	G	T	G	T			T	T	T	T	T	T	T	T	T	T	T	T	G	G	G	T	G	T
199	STOX2	rs11735366	chr4:184913299	C	21.366	C	G	C	C	C	C	C	C			C	C	C	C	C	C	C	C	C	C	C	C	G	G	C	G	C	G
200	STOX2	rs11736295	chr4:184913507	G	21.326			G	G							G	G	G	G					G	G	G	G			G	A	G	A
201	STOX2	rs146281849	chr4:184914457	A	0.120	A	A	A	A	A	A	A	A			A	A	A	A	A	G	A	A	A	G	A	A	A	A	A	A	A	A
202	STOX2	rs12651379	chr4:184914519	C	21.386	C	T	C	C	C	C	C	C			C	C	C	C	C	C	C	C	C	C	C	C	T	T	C	T	C	T
203	STOX2	rs28647598	chr4:184914536	G	44.808	G	C	G	G	G	G	G	G			G	G	G	G	G	G	G	G	G	G	G	G	C	C	G	C	G	C
204	STOX2	rs376210335	chr4:184914747	0		0	AT	0	0	0	0	0	0			0	0	0	0	0	0	0	0	0	0	0	0	0	0	0	0	0	0
205	STOX2	rs73010232	chr4:184915818	C	20.867	C	C	C	C	C	T	C	T			C	C	C	C	C	C	C	C	C	C	C	C	C	C	C	C	C	C
206	STOX2	rs79225980	chr4:184916134	A	2.157	A	G	A	A	A	A	A	A			A	A	A	A	A	A	A	A	A	A	A	A	A	A	A	A	A	A
207	STOX2	rs6552726	chr4:184916993	C	44.010	G	C	G	G	G	G	G	G	G	G	G	G	G	G	G	G	G	G	G	G	G	G	C	C	G	C	G	C
208	STOX2	rs6552728	chr4:184917157	C	25.819	C	T	T	T	C	T	C	T	T	T	T	T	T	T	T	T	T	T	T	T	T	T	C	C	C	T	C	T
209	STOX2		chr4:184917179	G	0.639	G	G	G	G	G	G	G	G	G	G	G	G	G	G	G	G	G	G	G	G	G	G	G	G	G	G	G	G
210	STOX2	rs2309955	chr4:184917562	A	44.010	G	A	G	G	G	G	G	G	G	A	G	G	G	G	G	G	G	G	G	G	G	G	A	A	G	A	G	A
211	STOX2	rs12642003	chr4:184917791	G	21.645	G	A	G	G	G	G	G	G	G	A	G	G	G	G	G	G	G	G	G	G	G	G	A	A	G	A	G	A
212	STOX2		chr4:184918288	G		G	G	G	G	G	T	G	T	G	G	G	G	G	G	G	G	G	G	G	G	G	G	G	G	G	G	G	G
213	STOX2	rs17090673	chr4:184918852	G	21.306	G	A	G	G	G	G	G	G	G	G?	G	G	G	G	G	G	G	G	G	G	G	G	A	A	G	A	G	A
214	STOX2	rs17090674	chr4:184919470	C	19.269	C	C	C	C	C	C	C	C			C	C	C	C	C	C	C	C	C	C	C	C	T	T	C	T	C	T
215	STOX2	rs10033149	chr4:184919684	C	42.133	T	C	T	T	T	C	T	C			T	T	T	T	T	T	T	T	T	T	T	T	C	C	T	C	T	C
216	STOX2	rs6835682	chr4:184920355	C	30.950	C	T	T	T	C	T	C	T			T	T	T	T	T	T	T	T	T	T	T	T	C	C	C	T	C	T
217	STOX2	rs131149751	chr4:184920382	A	19.349	A	A	A	A	A	A	A	A			A	A	A	A	A	A	A	A	A	A	A	A	C	C	A	C	A	C
218	STOX2	rs12498735	chr4:184921299	C	40.835	C	C	C	C	C	T	C	T			C	C	C	C			C	C	C	C	C	C	T	T	C	T	C	T
219	STOX2	rs17075203	chr4:184921471	G	2.157	G	C	G	G	G	G	G	G			G	G	G	G	G	G	G	G	G	G	G	G	G	G	G	G	G	G
220	STOX2	rs76113902	chr4:184921778	A	2.157	A	T	A	A	A	A	A	A			A	A	A	A	A	A	A	A	A	A	A	A	A	A	A	A	A	A
221	STOX2	rs72310239	chr4:184921915	TGT	41.314	TGT	TGT	TGT	TGT	TGT	0	TGT	0			TGT	TGT	TGT	TGT	TGT	TGT	TGT	TGT	TGT	TGT	TGT	TGT	0	0	TGT	0	TGT	0
222	STOX2	rs146806994	chr4:184921961	C	0.160	C	C	C	C	C	C	C	C			C	C	C	C	C	C	C	C	C	C	C	C			C	C	C	C
223	STOX2	rs7669647	chr4:184925597	A	38.518	G	A	G	G	G	A	G	A			G	A	G	G	G	G	G	G	G	G	G	G	G	G	G	G	G	G
224	STOX2	rs7694352	chr4:184925614	T	24.381	A	T	A	A	A	T	A	T			A	A	A	A	A	A	A	A	A	A	A	A	A	A	A	A	A	A
225	STOX2	rs7670377	chr4:184925740	G	35.563	G	G	G	G	G	G	G	G			G	G	G	G	G	G	G	G	G	G	G	G	A	A	G	A	G	A
226	STOX2	SNP	chr4:184925773	A		0	A	0	0	0	A	0	A			0	0	0	0	0	0	0	0	0	0	0	0	0	0	0	0	0	0
227	STOX2	rs6828396	chr4:184926387	C	37.700	T	C	T	T	T	C	T	C			T	T	T	T	T	T	T	T	T	T	T	T			T	T	T	T
228	STOX2	rs59029519	chr4:184926540?	0		G	G	G	G	G	0	G	0			G	G	G	G	G	G	G	G	G	G	G	G			G	G	G	G
229	STOX2	rs13147432	chr4:184926627	A	50.000	C	C	C	C							C	C	C	C	C	C	C	C	C	C	C	C	C	C	C	C	C	C
230	STOX2	rs67630164	chr4:184926673	TTTAAAA	36.122	0	0	0	0	0	7	0	7			0	0	0	0	0	0	0	0	0	0	0	0	0	0	0	0	0	0
231	STOX2	rs72697712	chr4:184928703	C	0.978	C	C	C	C	C	C	C	C			C	T	C	C	C	C	C	C	C	C	C	C	C	C	C	C	C	C
232	STOX2	rs72697714	chr4:184928929	T	1.018	T	T	T	T	T	T	T	T			T	C	T	T	T	T	T	T	T	T	T	T	T	T	T	T	T	T
233	STOX2	rs7655396	chr4:184928980	C	21.645	T	T	T	T	T	T	T	T			T	C	C	C	C	C	C	C	C	C	C	C	C	C	C	C	C	C
234	STOX2	rs114157769	chr4:184929455	T	2.296	T	A	T	T	T	T	T	T			T	T	T	T	T	T	T	T	T	T	T	T			T	T	T	T
235	STOX2	rs75082346	chr4:184929717	A	1.977	A	G	A	A	A	A	A	A			A	A	A	A	A	A	A	A	A	A	A	A	A	A	A	A	A	A
236	STOX2	rs28655984	chr4:184930023	G	19.529	G	A	G	G	G	G	G	G			G	G	G	G	G	G	G	G	G	G	G	G	G	G	G	G	G	G
237	STOX2	rs66511946	chr4:184932935	A	24.840	A	A	A	A	A	A	A	A			A	G									A	A			A	G	A	G
238	STOX2	rs6810667	chr4:184935603	C	41.254	C	C	C	C	T	T	C	T			C	C									C	C			C	C	C	C
239	STOX2	rs6836767	chr4:184935835	T	45.787	A	T	T	T	A	A	A	T			T	T	T	T	T	T	T	T	T	T	T	T			T	T	T	T
240	STOX2	rs112565268	chr4:184936024	T	2.596	T	C			T	T							T	T	T	T	T	T	T	T								
241	STOX2	rs11733294	chr4:184936136	C	42.772	C	T	T	T	C	C	C	T	T	T	T	T	T	T	T	T	T	T	T	T	T	T	T	T	T	T	T	T
242	STOX2	rs181346894	chr4:184936156	G	0.080	G	G	G	G	G	A	G	A	G	G	G	G	G	G	G	G	G	G	G	G	G	G	G	G	G	G	G	G
243	STOX2	rs3811778	chr4:184936547	T	21.336	T	C	C	C	T	T	T	C	C	C	C	C	C	C	C	C	C	C	C	C	C	C	C	C	C	C	C	C
244	STOX2		chr4:184936601	A		A	A	A	A	A	A	A	A	A	A	A	A	A	A	A	CTA	A	CTA	A	A	A	A	A	A	A	A	A	A
245	STOX2	rs3811777	chr4:184936604	A	21.895	A	A	A	A	G	G	A	G	A	A	A	A	A	A	A	A	A	A	A	A	A	A	A	A	A	A	A	A
246	STOX2	rs3811776	chr4:184936615	T	21.326	T	C	C	C	T	T	T	C	C	C	C	C	C	C	C	C	C	C	C	C	C	C	C	C	C	C	C	C
247	STOX2	rs3841107	chr4:184936677	TTTT		TTTT	0	0	0	TTTT	TTTT	TTTT	0	0	0	0	0	0	0					0	0	0	0	0	0	0	0	0	0
248	STOX2	rs3811775	chr4:184936790	G	21.296	G	A	A	A	G	G	G	A			A	A	A	A	A	A	A	A	A	A	A	A			A	A	A	A
249	STOX2	rs2309956	chr4:184937312	G	43.171	G	A	A	A	G	G	G	A			A	A	A	A	A	A	A	A	A	A	A	A			A	A	A	A
250	STOX2	rs2871386	chr4:184937365	T	42.712	T	C	C	C	T	T	T	C			C	C	C	C	C	C	C	C	C	C	C	C			C	C	C	C
251	STOX2	rs10026246	chr4:184937370	C	49.880	T	C	C	C	T	T	T	C			C	C	C	C	C	C	C	C	C	C	C	C			C	C	C	C
252	STOX2	rs62339733	chr4:184937736	C	13.538	C	A	A	A	C	C	C	A			C	A	A	A	C	A	A	A	C	A	A	A			C	A	C	A
253	STOX2	rs138948095	chr4:184938129	T	0.639	T	T	T	T	T	A	T	T			T	T	T	T	T	T	T	T	T	T	T	T			T	T	T	T
